# Olfactory Hallucinations without Clinical Motor Activity: A Comparison of Unirhinal with Birhinal Phantosmia

**DOI:** 10.3390/brainsci3041483

**Published:** 2013-11-15

**Authors:** Robert I. Henkin, Samuel J. Potolicchio, Lucien M. Levy

**Affiliations:** 1 Center for Molecular Nutrition and Sensory Disorders, The Taste and Smell Clinic, 5125 MacArthur Blvd, NW, Suite 20, Washington, DC 20016, USA; 2 Department of Neurology, The George Washington University Medical Center, 2150 Pennsylvania Avenue, NW, 7th Floor, Washington, DC 20037, USA; E-Mail: spotolicchio@mfa.gwu.edu; 3 Department of Radiology, The George Washington University Medical Center, 900 23rd Street, NW, Washington, DC 20037, USA; E-Mail: llevy@mfa.gwu.edu

**Keywords:** phantosmia, hallucination, aura, olfaction, taste, phantageusia, epilepsy, Valsalva, GABA, brain plasticity

## Abstract

Olfactory hallucinations without subsequent myoclonic activity have not been well characterized or understood. Herein we describe, in a retrospective study, two major forms of olfactory hallucinations labeled phantosmias: one, unirhinal, the other, birhinal. To describe these disorders we performed several procedures to elucidate similarities and differences between these processes. From 1272, patients evaluated for taste and smell dysfunction at The Taste and Smell Clinic, Washington, DC with clinical history, neurological and otolaryngological examinations, evaluations of taste and smell function, EEG and neuroradiological studies 40 exhibited cyclic unirhinal phantosmia (CUP) usually without hyposmia whereas 88 exhibited non-cyclic birhinal phantosmia with associated symptomology (BPAS) with hyposmia. Patients with CUP developed phantosmia spontaneously or after laughing, coughing or shouting initially with spontaneous inhibition and subsequently with Valsalva maneuvers, sleep or nasal water inhalation; they had frequent EEG changes usually ipsilateral sharp waves. Patients with BPAS developed phantosmia secondary to several clinical events usually after hyposmia onset with few EEG changes; their phantosmia could not be initiated or inhibited by any physiological maneuver. CUP is uncommonly encountered and represents a newly defined clinical syndrome. BPAS is commonly encountered, has been observed previously but has not been clearly defined. Mechanisms responsible for phantosmia in each group were related to decreased gamma-aminobutyric acid (GABA) activity in specific brain regions. Treatment which activated brain GABA inhibited phantosmia in both groups.

## 1. Introduction

Perception of visual and auditory hallucinations in the absence of any apparent stimulus has been recognized since antiquity but similar perceptions of olfactory hallucinations may not have been recorded until 131 A.D. when Aretaeus, the Cappodocian wrote “a heavy smell sometimes preceded the accession of a paroxysm” [1]. Association of an odor preceding a subsequent seizure-like event was emphasized with his statement “sometimes the smell of the gagate stone makes them fall down” [1]. The odor preceding the seizure was considered an aura, a term introduced by Pelop, the master of Galeni to describe literally “a breath of air” [2]. The term was then associated with the Latin translation as odor or breeze, which was subsequently applied to all sensations preceding a seizure, and recognized as a spontaneous sensation without a specific initiating stimulus. Auras clinically associated with a subsequent seizure were considered to represent manifestations of early stages of build up of abnormal, synchronous electrical activity [3].

Historically, all sensory phenomena preceding a seizure were classified under the general term hallucination although the modus operandi for each sensory modality might differ and the mechanism(s) which initiated the so-called hallucinatory activity related to a multiplicity of antecedent causes. The word hallucinate derives from the verb to dream and hallucination defined as a dream-like state with perception of objects with no reality [4] clinically described as a disorder of the nervous system; this term may have been first introduced clinically in 1837 in Esquirol’s textbook [5] and, hence, it acquired a pathological implication usually related to an altered mental state. The term apparition, derived from the verb appearance [4], defined simply as an unusual appearance [4], is usually applied to visual perception of a non-existent ghostly figure without any clinical implication. The term phantom has been applied to these same phenomena defined as something apparent to the sense, e.g., a ghost or spectre, but without substantial existence or initiating stimulus [4]. These terms have been commonly used to describe transient visual or auditory phenomena, which are usually frightening or threatening although their physiology was not clearly specified. The terms aura, dreamy state and hallucination, describing the same fixed sensation have, on occasion, been used and considered interchangeable, as with Jackson’s localization of a dreamy state prior to a seizure related to a temporal lobe lesion [6,7,8]. 

There is considerable confusion related to diversity of auras [9,10,11,12,13] and to what an aura represents. On the one hand aura has been formally defined as “that portion of the seizure which occurs before consciousness is lost and for which memory is retained afterwards” [14,15,16]. This suggests aura is part of the seizure complex [17,18,19,20], is associated anatomically with the mechanism initiating the seizure (tumor, mesial temporal sclerosis, *etc*.) and has value in localizing [13,21,22,23,24,25,26] or lateralizing [14,16,17] seizure onset. Attempts have been made to classify aura on a systematic basis (e.g., simple and complex [27]). Some investigators suggested aura was the whole seizure [16] whereas others suggested that it only preceded the seizure [10]. On the other hand, several investigators questioned the localizing value of aura [28,29,30,31] and some considered it an unnecessary component of epilepsy, albeit auras of taste and smell were considered reliable indicators of temporal lobe lesions [27]. With stereoelectroencephalography, particularly in amygdala, temporal or olfactory bulb regions, olfactory hallucinations were reported with electrical brain stimulation [21,24,32] whereas gustatory hallucinations were reported with stimulation in hippocampus and amygdala regions [32,33,34]. We have extended these concepts by use of techniques of functional magnetic resonance imaging (fMRI) of brain [35] and by use of metabolic studies using magnetic resonance spectroscopy (MRS) [36] to describe the hallucinatory activities of these patients. By use of these techniques we have been able to develop both neurological and metabolic concepts upon which this hallucinatory activity is based.

A pathophysiological dissociation of seizure and aura was first proposed by Jackson who suggested auras might arise connected to but not in the seizure focus [37,38,39]. Sperling *et al*. using intracranial electrodes found limbic EEG correlates of auras lacking in patients who had specific limbic seizure localization [31]. Penfield described “experiential auras” elicited by electrical stimulation of temporal neocortex, not limbic cortex [21]. Gloor *et al*. stated aura represented activation of a neuronal network [24,40,41]. Auras have been associated with simple [42] and complex [30] partial seizures and considered to arise from specific brain regions; resection of this region (defined by subdural EEG recordings) resulted in cessation or significant reduction of these seizures in 60% of patients in one study [42]. However, as noted, the region from which EEG discharges arose may not be the only region in which functional alteration produced clinical features of simple partial seizures [11,32,42,43,44,45,46]. Using brain fMRI we have shown localization of phantosmia (*i.e.*, an olfactory hallucination in the absence of any external odor stimulus [35]) in frontal, orbitofrontal and cingulate cortex and for phantageusia (*i.e.*, a gustatory hallucination in the absence of any oral stimulus) in insular cortex [35].

Auras have persisted even after removal of pathological substrate for seizure discharge [24,40,41]. Conversely, patients with simple partial seizures related to focal temporal lobe activation may not exhibit aura of any type but after successful surgical resection of a localized focus which eliminates seizures with or without a motor component, aura may appear; this aura may be of any sensory modality but an intermittent recurrent olfactory aura or phantosmia is common (S.J. Potolicchio, unpublished observations). While most patients considered elimination of seizures with or without a motor component as successful seizure treatment following surgical central nervous system (CNS) resection, aura onset was considered a negative post operative component. Aura believed to be the beginning of convulsive attack or seizure [45,47], following successful CNS surgery for intractable epilepsy, has been reported to persist albeit patients were seizure free (*i.e.*, with or without motor components [47]). These results relate to auras of many types including visual, auditory, somatosensory, viscerosensory, gustatory or olfactory [47]. 

Patients with both temporal lobe epilepsy [14,24] and migraine headaches [48,49,50,51,52,53,54] also reported olfactory auras prior to seizure or headache pain onset, respectively. One group of authors related these phantosmias to trigeminal nerve activation [55]. These auras were related to suspected CNS pathology in temporal lobe in epilepsy (v.i.) and initially to medial temporal lobe ischemia in migraine [48]. However, more recent studies have considered the physiological substrate of migraine aura to relate to cortical spreading depression and blood flow changes more related to oligemic neuronal dysfunction rather than primary cerebrovascular ischemia. Odor character was usually reported as obnoxious or unpleasant. Phantosmia in patients with migraine on occasion was familial with both mother and daughter experiencing the same type of olfactory aura and headache [50]. This may not be unexpected since a positive family history of migraine has been reported in 20%–70% of cases [48,56,57]. Patients with auras and temporal lobe epilepsy commonly exhibited EEG abnormalities [16,32] whereas those with aura and migraine usually exhibited both normal EEGs [34] and CT brain scans [34]; however, these latter patients can also exhibit paroxysmal EEG and other neuronal abnormalities [58,59,60,61,62] suggesting an underlying neuronal component in the generation of these symptoms [63,64]. The aura of migraine has been considered a separate phenomenon from the headache [65], considered by some investigators to be determined by aura suceptibility genes [66], the pain associated with other genes [67,68]. Some investigators considered migraine aura characterized by a wave of oligemia [67,69,70], considered the human counterpart of Leão’s spreading depression [71] that passes across the cortex.

Some investigators reported more than 50% of patients with temporal lobe epilepsy had an aura of some type [14,17,18]. Estimates of frequency of olfactory auras associated with seizure activity vary considerably ranging from 1% or less [42,72], to 8% [23,73], to 16%–20% [17,74] to over 30% [75]. Reports of incidence of phantosmia in patients with temporal lobe foci vary from 6% to 12% [32,51] whereas with occipital or central foci incidence was 4% and 3%, respectively [32]. Gustatory hallucinations in patients with seizures have an incidence reportedly about one-half that of olfactory hallucinations [16,33] or as low as less than 1% [73]. Gustatory hallucinations have been reported in patients with temporal lobe epilepsy [33,34]. While reports of visual and auditory hallucinations are much more frequent than olfactory or gustatory hallucinations in patients with epilepsy, these latter sensations have been described as being the most prominent and consistent features of a seizure disorder [14,27,34].

Most frequent reports of olfactory hallucinations without subsequent clinical motor activity are in psychiatric literature [76,77,78,79] with estimates ranging from 11% to 83% among patients with schizophrenia [80,81,82,83,84] and from 19% to 33% among patients with major depressive illness [85]. Phantosmia has been reported in patients with severe depression [86]. Patients with schizophrenia commonly do not describe their phantosmia distinctly; they usually use vague descriptive terms such as strange or peculiar although phantosmia is generally considered unpleasant. About 25% of patients with what has been termed the olfactory reference syndrome [87,88,89,90] reported perception of a foul odor emanating from a body orifice other than nose or mouth or on occasion from the body as a whole (e.g., skin) unrelieved by any maneuver to remove the odor such as a cleaning procedure; this syndrome is characterized by this fixed, monosymptomatic odor. Successful treatment of this syndrome has been reported by use of aripiprazole presumably as its use as a partial agonist of dopamine D_2_ receptors in the olfactory bulb [91]. On the other hand, psychiatric symptoms have been associated with several types of organic brain lesions [92,93,94].

More recently, olfactory hallucinations without subsequent clinical motor activity have been reported in as many as 29% of patients with several types of eating disorders [95], in patients with hypochondriasis [96] or associated with enuresis [97]. Olfactory hallucinations have occurred after use of intravenous caffeine infusion [98,99] but with these patients having associated seizure induction. Gustatory and olfactory hallucinations have occurred after treatment with bupropion [100]. Phantosmia without associated seizure induction has also been reported in over 60% of patients who reported hyposmia following head injury [101], following a variety of other clinical conditions [102], following intake of therapeutic drugs [103] and for no clinically apparent cause [35,104,105].

Incidence of phantosmia without seizure activity has been reported by other investigators to occur in as many as 10%–31% in a randomly selected group of factory workers [106]; this subject has recently been reviewed [107]. However, among patients with partial seizures one group of investigators estimated that only 0.9% exhibited what they described as olfactory epileptic auras [72] whereas another group estimated that 5.5% of these patients exhibited these olfactory auras [108]. Our incidence estimates of phantosmia without associated epileptic activity among patients with self reported and measured loss of smell and/or taste acuity varied between 40% and 60% of the group of over 5000 patients studied at The Taste and Smell Clinic in Washington, DC, USA [105].

Olfactory hallucinations have also occurred without seizure activity as a presenting symptom in patients with intracerebral hemorrhage [109,110], with glioblastoma multiforme [111] or with electrical stimulation of the olfactory bulb and tract [112]. Olfactory hallucinations have occurred in local nasal disease related to rhinosinusitis [113]. Patients with Parkinson disease have been reported to exhibit phantosmia without associated seizure activity [114,115,116] and after treatment with rasagiline [117]. Olfactory hallucinations without associated seizure activity have been reported following radiation therapy [118].

On the other hand phantosmia has been treated with surgical excision of the olfactory epithelium [119] following resection of the olfactory bulbs [120] by use of venlafaxine [121] or by use of topiramate [122]. One group of investigators reported that what they described as “idiopathic phantosmia” disappeared spontaneously after the symptoms were present for a period of more than five years [123].

However, symptoms associated with these phantosmias have been confusing and considered by some investigators to be psychogenic, nonepileptic seizures [124], with altered brain connectivity among some of these patients [125]; however, these workers appear to lack understanding of the biochemical parameters underlying these symptoms [35,36,105].

We recognized that patients with birhinal phantosmia without subsequent clinical motor activity occurred commonly after a clinical event which induced loss of smell [102,105]; these patients are a much larger group than those with phantosmia associated with either epilepsy, brain tumors or migraine. In a survey of smell among over 5000 men and women in the US who attended a commercial fragrance establishment about 7% reported chronic smell loss or hyposmia [126]. Extrapolation of this number to the total US population suggests an estimated 21 million people in the US may have some form of hyposmia (vi). In another publication we estimated that 40% to 60% of patients with hyposmia, regardless of cause, reported birhinal phantosmia and/or global oral phantageusia without clinical motor activity [102,105]. These estimates are supported, in part, by investigators who reported phantosmia without subsequent clinical motor activity in a small group of patients with what they called “simple partial status epilepticus” whom they considered greatly underdiagnosed [3]. In another study involving 1000 patients evaluated at a Japanese otolaryngological clinic for any symptom related to any pathology of the head and neck about 14% spontaneously reported presence of phantageusia without clinical motor activity [127].

With respect to terminology we defined phantosmia as an odor in the nose in the absence of any external initiating stimulus [102,105,128]. Phantosmia may have been clinically first described systematically by Jackson as a transient, unpleasant odor preceding a seizure [6,7,8]. Descriptions were almost uniformly birhinal, unpleasant, usually a rotten, sickening, burning, bad or indescribable odor [38,129]. They were also described as tobacco-like, smoky or sulfur-gas-like. We classified phantosmias into three general types: (1) cacosmic (rotten, decayed or fecal [102,103,105,130], (2) torquosmic (burned, metallic, chemical-like [102,103,105,130]), or mixed, a combination of both cacosmia and torquosmia [102,103,105,130]. Phantosmias as such were previously recognized by others [38,131,132,133] with associated EEG abnormalities, changes in positron emission tomography (PET), computerized tomography (CT) and magnetic resonance imaging of brain (MRI) [26,47] and with typical subsequent seizure activity [10,11,12,13] related to temporal lobe [38,131,132,133] and other brain tumors [21,22,26]. Seizures associated with brain tumors were commonly reported to begin with abnormal sensations of global oral taste or birhinal smell (auras) along with masticatory movements and spitting [75] and were called uncinate fits by Féré [134] who suggested that taste and smell sensations originated in the uncinate gyrus. 

We defined phantageusia as a global taste in the entire mouth in the absence of any initiating oral stimulus [102,103,105,135]. Prior descriptions of phantageusias as such were also predominantly present in the entire oral cavity, were unpleasant and varied in character from rotten, bitter, salty, acidic or burned to pleasant or even sweet [33,34,102,105,135]. We classified phantageusias as either cacogeusic, torquegeusic or mixed [128], similar to phantosmias. Phantageusias [136,137] have also been reported preceding seizures, usually associated with brain tumors [6,7,8].

Association of both birhinal phantosmia and global oral phantageusia without subsequent clinical motor activity with loss of smell and taste acuity, respectively, [102,105] is consistent with changes in CNS plasticity. Onset of similar hallucinatory behavior has been previously observed with sensory losses of visual and auditory systems; association of visual hallucinations with loss of visual acuity has been reported in patients with cataract [138], and with age, sometimes referred to as Bonnet syndrome [139,140,141,142,143]; auditory hallucinations without subsequent clinical motor activity have been reported in patients with loss of auditory acuity [144,145,146].

Most patients with sensory hallucinations reported these phenomena as involving an entire sensory system; most visual hallucinations involved both visual fields, auditory hallucinations both auditory fields, olfactory hallucinations, both nares and gustatory hallucinations in the entire oral cavity. However, unilateral sensory hallucinations have been reported in only one visual field [147,148] or one ear [149,150,151,152,153,154]. In a preliminary study we previously described a patient group with olfactory hallucinations in only one naris [155]. These patients independently related a strikingly similar history; insidious onset of an obnoxious, initially unirhinal odor without any initiating external stimulus which initially arose without any antecedent cause, initially occurring spontaneously, intermittently, but over time increasing in frequency, initiated by coughing, laughing, crying, shouting, vigorous nasal inhalation and/or exhalation or sneezing and initially inhibited spontaneously but over time only relieved by sleep or a Valsalva type maneuver [155]. These patients were usually young women who, because of associated EEG abnormalities, were commonly considered to have a form of partial seizure disorder despite absence of antecedent or subsequent clinical motor activity and were initially treated with various anticonvulsant agents which did not induce any symptomatic relief.

Whereas we have previously briefly reported these patients [155], as well as their fMRI and biochemical responses [104,156], it is the purpose of this study to describe these patients in detail for the first time. To our knowledge, these patients reflect the only group in whom phantosmia (and/or phantageusia) without subsequent clinical motor activity is either spontaneously initiated or inhibited and subsequently inhibited by specific physiological maneuvers. We will contrast these patients with the much larger, more common but previously poorly defined patient group with birhinal phantosmia without subsequent clinical motor activity who initially experienced an illness or some event which impaired their smell (and/or taste) acuity and who subsequently developed birhinal phantosmia.

## 2. Material and Methods

### 2.1. Subjects

Subjects were 128 consecutive patients with phantosmia [102,105] without subsequent clinical motor activity from a total group of 1272 consecutive patients evaluated at The Taste and Smell Clinic, Washington, DC, USA from 1985 to 1995 for various abnormalities of taste and/or smell dysfunction and six consecutive normal volunteers recruited from the normal volunteer pool at the National Institutes of Health (NIH), Bethesda, MD, USA. 

Normal volunteers were three men and three women, all right handed Caucasians, aged 21–34 years, who were in good health and not taking any medication. Their taste and smell function were within normal limits. The subjects served as volunteers only for the functional magnetic resonance brain imaging and magnetic resonance spectroscopy studies performed at the National Institute of Neurological Diseases and Stoke, NIH, Bethesda, MD, USA.

The 128 patients, studied in a retrospective manner, were all those of the 1272 who had symptoms of phantosmia, either unirhinal or birhinal, and also had sleep-deprived EEGs with nasopharyngeal leads as part of their initial evaluation (Table 1). Eighty-eight of these 128 presented with hyposmia but also other sensory abnormalities including hypogeusia, phantageusia, aliageusia (an obnoxious taste in the mouth initiated by presence of food or drink and related directly to the ambient tastant [102,103,105,130]) and/or aliosmia (presence of an obnoxious smell in the nose initiated by presence of an external vapor and related directly to the ambient odor [102,103,105,130]). These patients developed birhinal phantosmia which was either persistent or intermittent, neither cyclic nor episodic (Table 1) but related to a specific clinical condition which initiated their hyposmia including head injury [101,105], post influenza-like illness [105,157], post nasal surgery [102,105,128] or several other medically related conditions [105,158,159]. Seventy-six (86%) of these 88 patients initially presented with hyposmia [102,105]; 12 presented with complaints of either phantageusia [102,105,135], aliosmia [105,130], aliageusia [105,130] or some symptom combination. Although many other patients among the 1272 evaluated reported birhinal phantosmia [102,105], the relatively small patient number who met these latter criteria in this report (88 of 1272) was due to the fact that only patients with birhinal phantosmia who had EEGs with nasopharyngeal leads as part of their initial evaluation were included in this study.

**Table 1:** Clinical characteristics of patients with phantosmia.

Condition	Cyclic Unirhinal Phantosmia (CUP)	Birhinal Phantosmia with Associated Symptoms (BPAS)
*Patient number*	40	88
Gender	30 women, 10 men ^z^	47 women, 41 men
Ratio women:men	3:1	1:1
*Age (year)*	31 ± 1 *^, a^ (19–49 years)	46 ± 2 (12–82 years)
Men	31 ± 1 ^a^ (19–46 years)	41 ± 3 (12–81 years)
Women	30 ± 1 ^a^ (19–49 years)	49 ± 2 (19–82 years)
*Symptom duration before evaluation (year)*	7.2 ± 1.0 ^a^ (0.5–28 years)	1.8 ± 0.2 (1 week–10 years)
*Initial phantosmia character*		
Cacosmic	31 ^†, w1^	20 ^†^
Torquosmic	9 ^w^	68 ^w2^
*Onset of phantosmia*		
Unirhinal	40 ^w, w1^	0
Birhinal	0 ^w^	88 ^w2^

* Mean ± SEM; ^†^ Patient Number; ( ) Range; *CUP vs. BPAS*: ^a^ *p* < 0.001, *t* test; ^w^ *p* < 0.001, *X*^2^ (torquosmic, unirhinal, birhinal); ^z^ *p* < 0.05, *X*^2^ (women *vs*. men); *CUP*: ^w1^ *p* < 0.001, *X*^2^ (cacosmic *vs*. torquosmic, unirhinal *vs*. birhinal); *BPAS*: ^w2^ *p* < 0.001, *X*^2^ (cacosmic *vs*. torquosmic, unirhinal *vs*. birhinal).

Forty of these 128 were all those who presented with a history of a cyclic, episodic, initially unirhinal phantosmia as their only complaint (Table 1).

To differentiate these two groups we labeled patients with cyclic, episodic, recurrent, initially unirhinal phantosmia as having cyclic unirhinal phantosmia (CUP); those who presented with non-cyclic, non-episodic birhinal phantosmia and hyposmia (and/or other symptoms of taste and/or smell dysfunction) and were labeled as having birhinal phantosmia with associated symptoms (BPAS). 

The purpose of this study is to describe, in a retrospective manner, the symptoms which define these two types of olfactory hallucinations and then, based upon these descriptions, describe the procedures developed to understand the putative neurological and metabolic parameters responsible for these hallucinatory activities. The hypothesis underlying this study relates to an attempt to develop the neurological and metabolic parameters associated with and putatively responsible for the development of these hallucinations.

All studies were approved by the Institutional Review Boards of the Clinical Center of the National Institutes of Health, the Georgetown University Medical Center and the George Washington University Medical Center. All subjects who participated in these studies formally signified their agreement after specific explanation of any study protocol which they signed.

### 2.2. Clinical History

Each patient had a complete medical history with special emphasis on taste and smell dysfunction [102,105,160,161]. Examination of head and neck was performed in each patient (including indirect nasopharyngoscopy with use of a topically applied vasoconstrictive agent (e.g., 0.25% neosynephrine)) to assist visualization of nasal airways [102,105,128].

Taste function tests (gustometry) were performed using the entire oral cavity with a standard forced choice, three stimuli, staircase drop technique using NaCl (salty), sucrose (sweet), HCl (sour) and urea (bitter) as stimuli [102,105,161] by which detection thresholds (DT), recognition thresholds (RT) and estimations of magnitude intensity (ME) for each tastant were obtained [102,105,135,161]. Four types of taste loss were defined (ageusia, Type I, II, III) based upon loss severity with ageusia the most severe, Type I, II and III in decreasing severity as type increased [105,128,160]. Ageusia was defined as inability to detect or recognize any tastant (DT = 0, RT = 0, ME = 0). Type I hypogeusia was defined as inability to recognize any tastant (DT > 0, RT = 0, ME = 0). Type II hypogeusia was defined as decreased ability to detect or recognize tastants but ability to do so at a level less than normal (DT < normal, RT < normal, ME < normal). Type III hypogeusia was defined as normal ability to detect and recognize tastants but decreased ability to quantitate tastant intensity (DT = normal, RT = normal, ME < normal). Taste distortions (dysgeusias) were quantified by questionnaire [102,105,135] on a scale from 1 to 100 and labeled aliageusia or phantageusia with subcategories of aliageusia and phantageusia defined as noted above as cacogeusic, torquegeusic, or mixed [105,128].

Smell function tests (olfactometry) were performed with a standard forced choice three-stimuli, staircase sniff technique using pyridine (pungent), nitrobenzene (bitter almond), thiophene (petroleum-like), amyl acetate (banana oil) and menthone (minty) as stimuli [102,105,160] by which DT, RT and ME for each odorant was obtained [102,105,130,160,161]. Four types of smell loss were defined (anosmia, Type I, II, III) based upon severity of loss with anosmia the most severe and Type I, II, III in decreasing severity as type increased. Definitions of smell loss type were similar to those described for taste loss [102,105,160]. Both unirhinal and birhinal smell function tests were performed in each patient; results did not differ significantly in any patient. Smell distortions were defined similar to taste distortions and labeled aliosmia and phantosmia with subcategories defined as cacosmic, torquosmic or mixed [102,105,160].

#### 2.2.1. Electroencephalographic (EEG) Studies

EEGs were performed in all patients after sleep deprivation for 12–24 h; each refrained from taking caffeine-containing beverages during this period but ate a complete meal prior to study. Nasopharyngeal leads were employed in all studies. Studies were performed on an 8, 10, 16, or 21 channel electroencephalograph monitored by a registered EEG technician. All examinations included photostimulation and hyperventilation performed by International EEG Society standard techniques. All records were initially interpreted by a Clinical Neurophysiology Board certified physician (SP) without specific knowledge of patient clinical status and reviewed independently by an ANA board eligible neurophysiologist (EC, see acknowledgements). 

CUP: Prolonged recordings (30 min–24 h) were obtained in each patient. Twenty-four hour recordings were obtained in 11 patients with 30–180 min recordings obtained in the remainder. Twenty-seven of the 40 records were interpreted similarly by both neurophysiologists. In 15 records clinical interpretations differed; each was reinterpreted by each neurophysiologist and a consensus report was issued. 

BPAS: Twenty-four hour recordings were obtained in 12 patients with 30–180 min recordings obtained in the remainder. Each of the 88 records was interpreted similarly by both neurophysiologists.

#### 2.2.2. Neuroimaging Studies

Computer assisted tomographic (CT) scans of brain was performed on 29 patients with CUP and 55 patients with BPAS on either a GE (Syracuse, NY, USA) or Siemens Vision (Iselin, NJ, USA) unit. MRI of brain was performed using a quadrature headcoil on five patients with CUP and two patients with BPAS on a GE or a Siemens 1.5 Tesla scanner. One patient with CUP had both a CT scan and MRI study; one patient with CUP had two MRI studies. One patient with BPAS had two CT brain studies and one had four such studies. A venous angiogram of the orbit was performed in one patient with CUP and two had four vessel cerebral angiograms based upon initial abnormal findings on CT scans. Skull roentgenograms were performed in 20 patients with CUP and in six with BPAS. Sinus roentgenograms were performed in 12 patients with CUP and in 14 with BPAS. Tc99m brain scans were performed in six patients with BPAS. All results were interpreted by an ARA board certified neuroradiologist (LML) without any specific knowledge of the clinical status of the patient. Results were reviewed by at least one author (RH and/or SP) and no disputed readings were obtained.

#### 2.2.3. Functional Magnetic Resonance Brain Imaging (fMRI)

Two patients with CUP (one man, one women) [104,156] and two patients with BPAS (one man, one woman) [35] were studied using fMRI. For these studies each patient was placed in the 1.5T Siemens MR scanner using a quadrature head coil. Under standard conditions as shown previously for odor memory [162] and taste memory [163], normal volunteers and patients with CUP and BPAS were requested to think of (remember) their phantosmia and in patients with CUP to attempt to initiate and inhibit it. Results were quantitated by calculation of significant activation of pixel number activated in specific anatomical regions by each condition [162,163].

#### 2.2.4. Magnetic Resonance Spectroscopy (MRS)

Two patients (two women) with CUP, three patients (two women, one man) with BPAS and the six normal volunteers were studied using MRS as previously described [36,164]. Subjects were placed in a 1.5T General Electric (GE) MR scanner using a quadrature head coil. Under standard conditions specific brain regions (occipital, cingulate and insular cortex) were evaluated for levels of gamma-aminobutyric acid (GABA) and creatine. Patients with CUP had onset of their phantosmia and phantageusia induced either spontaneously or by hyperventilation at initiation of these studies. Patients with BPAS had persistent phantosmia during MRS. Data were analyzed using software previously developed and utilized by L.M. Levy *et al*. [36,164]. Results were calculated as ratio of GABA: creatine in each region studied and comparisons were made between each group studied.

#### 2.2.5. CO_2_ Inhalation

Ten patients (three men, seven women) with CUP were treated with inhalation of 50% CO_2_ delivered through nasal prongs within a nasal mask for periods of 10–60 s during presence of unirhinal phantosmia. Each patient was seated in a chair, prongs fitted snugly into the nose and CO_2_ flow adjusted to a maximum of 50 lbs/in^2^.

#### 2.2.6. Unirhinal Cocaine Administration

A solution of 10% cocaine was administered by nasal spray into the upper nasal cavity on the side of phantosmia on one occasion in two patients (two women) with CUP during the time they reported unirhinal phantosmia and birhinally into the upper nasal cavity in one patient (one woman) with BPAS during the time she reported birhinal phantosmia.

#### 2.2.7. Repetitive Transcranial Magnetic Stimulation (rTMS)

Two right handed patients (two women) with CUP and four right handed patients (two men, two women) with BPAS were studied using rTMS, as previously described [165,166]. Signal was generated by use of a Cadwell electromagnetic stimulator (Seattle, WA, USA) monitored with a TECA TD20 (Pleasantville, NY, USA) wave form generator, as previously described [165,166]. Stimulation was applied with a single quadrangular coil in three phases; initially two sham phases and then one active phase. In the first sham phase stimulation was applied at 40% of maximal output (40% of 1.5 Tesla or about 0.8 T) to each shoulder at Erb’s point and to the posterior neck. This stimulation resulted in significant movement of each shoulder and of head extension. In the second sham phase stimulation was applied at 10% of maximal output (20% of 1.5 Tesla or about 0.1 T). The stimulation was applied to each of four skull regions (left and right temporoparietal, occipital, frontal) and was considered a non-significant stimulation since no physical response occurred after this stimulation. A third set of stimuli at 60% maximal output (between 0.8 and 1.2 T since stimulus delivery was non-linear) was then delivered to each of these same four skull locations. This third set of stimuli was repeated at a specific response location (usually left temporoparietal) in patients in whom a change (usually lowering) in phantosmia intensity and/or change in phantosmia character occurred for three to six additional stimuli or until no further lowering or change in phantosmia character occurred. Stimulus frequency was 1 pulse per 1–2 s for 30–90 s. Stimulation was monitored in each phase by degree of flexion of muscle groups associated with stimulus application (e.g., right radial, median and/or ulnar nerve stimulation with left temporoparietal stimulation) and was graded on a six point scale with one, the least amount of muscle flexion and six, the greatest. Quantitative patient responses were elicited after each application in each skull location on a scale of 0–100 with 100 reflecting the greatest phantosmia intensity encountered, zero the absence (inhibition with disappearance) of phantosmia and intermediate numbers indicating intermediate changes of inhibition. Any change in phantosmia character was also reported. Response to stimulation was recorded for periods up to 60 months following this procedure. Following rTMS increases in specific blood plasma, erythrocytes and salivary growth factors were measured in CUP and BPAS [167].

#### 2.2.8. Statistics

Means of each parameter measured for each patient group were determined and differences evaluated by Student t test with *p* < 0.05 considered significant. Differences between sets of parameters were also evaluated by Chi square (*X*^2^, *F* test) and ANOVA with *p* < 0.05 considered significant.

## 3. Results

### 3.1. Patient Characteristics

CUP: Thirty women and 10 men had CUP (Table 1). Patients’ age ranged from 18 to 49 years (31 ± 1 years, mean ± SEM, (Table 1)). The ratio of women to men was 3:1. All patients developed symptoms post puberty. All were Caucasian. Symptoms were experienced 3 months–28 years (mean, 7.2 years) prior to presentation at The Clinic (Table 1). Initial symptom was always onset of an unirhinal phantom odor usually of a cacosmic type (78%). Each patient related a similar history; none had any prior knowledge of this symptom or syndrome. This syndrome consisted of two phases, an initial one (Table 1) associated with symptom onset and a subsequent one associated with symptom persistence (Table 2). No prior or subsequent clinical motor activity ever occurred in any patient.

**Table 2:** Odor character of patients with phantosmia.

Condition	CUP ^†^	BPAS ^†^
*Final odor character*		
Cacosmic	20 ^y1^	17
Putrid/rotten	20	17
Torquosmic	9 ^w^	70
Burned/smoky	2	22
Stale	2	0
Metallic	1	7
Fetid	1	0
Diesel-like exhaust fumes	1	0
Chemical/bitter	1	17
Sweaty	1	1
Excessively sweet	0	6
Body odor-like	0	1
Indescribable	0	16
Mixed	11 ^z^	1
*Initial phantosmia location*		
Unilateral	40 ^w, w1^	0
Right naris	23	
Left naris	17	
Bilateral	0 ^w^	88
*Final phantosmia location*		
Unilateral	25 ^w^	0
Right naris	15	0
Left naris	10	0
Bilateral	15 ^w^	88

**^†^** Patient Number; *CUP vs. BPAS*: ^w^ *p* < 0.001, *X*^2^ (torquosmic); ^z^ *p* < 0.05, *X*^2^ (unilateral, initial and final; bilateral, initial and final); *CUP*: ^w1^ *p* < 0.001, *X*^2^ (initial unilateral *vs*. bilateral), ^y1^ *p* < 0.02, *X*^2^ (cacosmic *vs*. torquosmic; unilateral (initial) *vs*. bilateral; cacosmic *vs*. mixed).

Initial Phase: Phantosmia usually occurred spontaneously, unirhinally, usually without any other symptom (Table 1). It occurred usually without warning and was an extremely unpleasant odor (commonly described as rotten, decayed, fecal (cacosmic) but also less frequently as chemical, metallic or burned (torquosmic)). It initially appeared in only one naris in the absence of any external ambient odor/or external stimulus and lasted from 5 to 10 s to as long as 10 to 20 min and then spontaneously disappeared (Table 2 and Table 3). The odor would then reappear, in the same naris again usually without any external stimulus after d, wk or mo, again lasting a few sec or min and then disappeared. After this second appearance and disappearance the interval between phantosmia onset and offset shortened such that it occurred with increasing frequency, intensity and duration eventually occurring in some patients daily lasting sec-min-hr and recurring as frequently as 5–20 times daily.

**Table 3:** Clinical history in patients with phantosmia.

Condition	CUP	BPAS
*Initial symptom*	Phantosmia (100% (40/40))	Hyposmia (80% (76/88))
*Symptom onset*		
Naris location	Unirhinal (100% (40/40))	Birhinal (100% (88/88))
Length	Momentary (s–min), Fleeting	Intermittent (10 s–24 h), Variable
Frequency	1–20×/day	1–10×/day or persistent
*Symptom progression*		
Naris location	Mainly unirhinal, occasionally birhinal	Birhinal only
Length	Gradual longer episodes 2–30 min; rarely persistent	No change (intermittent, persistent) or gradual symptom shortening with disappearance
Intensity	Increasing	No change or decreased intensity
Character	No change—always unpleasant	May vary, can change from one unpleasant odor to another (occasionally less unpleasant)
*Triggers initiating phantosmia*	Sneeze	None; onset is spontaneous
	Nasal Sniffing	Strong external odor may occasionally
	Nasal Blowing Out	trigger phantom (Allodynosmia)
	Rhinorrhea	
	Cough	
	Laugh	
	Loud Speech	
	Vigorous Physical Activity	
	Strong External Odor, may occasionally trigger phantom (Allodynosmia)	
*Physiological phantom inhibitors*	Sleep (30–180 min), usually in PM	None
	Total Nasal Blockage	May decrease over time spontaneously
	Valsalva	

After initial and second insidious onset all patients noted phantosmia was initiated following coughing, laughing, crying, sneezing, blowing the nose, loud speech or shouting, any intense emotional outburst, physical exertion, strenuous exercise, forced nasal inhalation and/or exhalation or hyperventilation (Table 3). One patient elicited phantosmia when diving into a swimming pool. No movement disorder, change in behavioral state, loss of consciousness, change in awareness of surrounding or automatism of any type was associated with phantosmia onset or presence. Over time half the patients, only on occasion, noted phantosmia (and/or phantageusia (v.i.)) onset after contact with a common, ambient vapor at its usual concentration (e.g., smell of brewing coffee, cigarette smoke, perfume, garlic or cutting onion) or taste of a flavorful food (e.g., taste of coffee, garlic, onion, *etc*.) labeled allodynosmia (for smell) and allodyngeusia (for taste), respectively, similar to allodynia (a painful sensation related to an usually perceived non-painful touch stimulus) [105]. In presence of these distortions they perceived these ambient vapors and oral tastes appropriately as the vapor or taste would be perceived normally (*i.e.*, no aliosmia or aliageusia, respectively). After removal of stimulating vapor or taste, phantosmia and/or phantageusia persisted as it did after any physiologically based initiating stimulus (e.g., sneezing, laughing) and continued until inhibited by one of the physiological maneuvers used by the patient to terminate phantosmia (and/or phantageusia) (v.i.). 

After a few weeks all patients recognized that phantosmia was inhibited by sleep (Table 3). Naps as short as 20–30 min, if deep enough sleep were obtained, alleviated phantosmia. This effect was most commonly noted if sleep were obtained in mid or late afternoon. Naps in younger patients were more effective in inhibiting phantosmia than in older patients. However, over time, naps became less effective in relieving phantosmia. During the initial phase of this syndrome no matter how intense or persistent the phantosmia, an overnight sleep was uniformly associated with phantom disappearance upon awakening in the morning. After an extended time period (>5 years) overnight sleep became somewhat less effective in relieving phantosmia (secondary phase). 

Independently, most patients discovered that a Valsalva-type maneuver terminated phantosmia (Table 3). Variations of this procedure included hanging the body trunk over a sofa and either holding breath or crying, snorting tap water or NaCl into the affected naris, or forced induction of emesis. After initial discovery of this technique, termination of phantosmia was uniformly successful and inhibition usually persisted for h. During this refractory period events such as sneezing or shouting did not reinitiate phantosmia. However, over time, while Valsalva still terminated phantosmia, length of inhibitory period became progressively shorter and less likely to resist reinitiation by crying, shouting, blowing of nose, or sneezing. 

Some patients discovered that plugging the naris in which phantosmia appeared reduced intensity or eliminated it; however, this was generally ineffective over relatively prolonged time periods since if a cotton plug were used it became soaked with nasal mucus and had to be replaced or removed which activated phantosmia, or if a rubber or plastic plug were used, it induced excess nasal mucus so that the patient had to remove the plug in order to retain normal nasal and oral mechanics which reactivated phantosmia. Some patients habitually breathed through their mouth with some phantosmia relief; however, return to nasal breathing with nose blowing, *etc*., reinitiated phantosmia. 

Acute coryza had a dichotomous effect; associated with nasal congestion, phantosmia was temporarily diminished or terminated but associated with sneezing and/or rhinorrhea, with or without nasal congestion, phantosmia was exacerbated (Table 3). One patient reported phantosmia inhibition coupled with hyposmia onset during recreational snorting of cocaine; cessation of snorting was associated with a gradual return of normal smell function and phantosmia. This patient also reported recurrent smoking tetrahydrocannanibol (THC) inhibited phantosmia without change in olfactory acuity; with termination of smoking, phantosmia gradually returned. This patient used this latter technique on multiple daily occasions to inhibit phantosmia with complete success on each occasion. Inhibition usually occurred 20–30 min after initiation of THC use and continued throughout the smoking process. Deffervescence occurred gradually, coincident with deffervescence of THC effects. 

After phantosmia terminated either spontaneously, following sleep or a Valsalva maneuver, no physiological carry-over (*i.e.*, no post-ictal-type symptoms) was present. Patients continued their normal daily activities without any change from their usual practice no matter how frequently they performed a Valsalva or some similar maneuver to relieve their phantosmia. Patients were usually embarrassed to perform a Valsalva maneuver in public and usually closeted themselves in some private area to perform this act; on occasion a patient might perform a Valsalva-type maneuver 5–20 times daily in order to obtain effective relief.

Patients were very disturbed by presence of this strong obnoxious odor. Phantosmia character was always constant, considered obnoxious and intolerable, causing the patient to focus on its presence because of its intensity and character. In 78% of patients the phantosmia was cacosmic, in the remainder it was torquosmic (Table 1). Although a few patients had difficulty describing the odor, once parameters of cacosmia and torquosmia were discussed each was able to categorize the odor specifically (Table 2). No patient reported ever previously experiencing such an intense, unpleasant, overwhelming sensation. In this sense, its presence interfered with patient activities although each could continue to and did perform usual work activities. At times, however, phantosmia intensity became so great that patients terminated normal daily activities, most to attempt to alleviate the symptom with a Valsalva-type maneuver (Table 3). Phantosmia presence was not associated with any behavioral, mental or consciousness change. No specific change in emotional state other than displeasure and discomfort with phantosmia presence was associated with its initiation or inhibition. No willful behavior of any type other than the specific physiological maneuvers described (v.s.) affected either phantosmia onset or termination.

In 21 of 40 (52.5%) patients once phantosmia became recurrent, some characteristic sensation alerted them prior to its appearance. These sensations varied from nasal appearance of an odor similar to phantosmia but of much less intensity (12 patients), a feeling of facial pressure (five patients), a metallic taste (one patient), an indescribable intranasal sensation (one patient) or depression (one patient). Time preceding phantosmia onset varied from sec to 3 h (two patients (1–59 s), 16 patients (1–59 min), three patients (1–3 h)).

Phantosmia occurred in absence of any clinical disorder and was of an unknown cause in 85% of patients (34 of 40, Table 4). A viral illness was associated with phantosmia onset in three (7.5%). Phantosmia occurred either during first trimester of pregnancy or immediately post partum in two (5%) (Table 4). Phantosmia arose after head injury in one patient (2%) (Table 4). One patient with phantosmia of idiopathic onset had a prior diagnosis of progressive systemic sclerosis, which was in remission (without treatment) at time of phantosmia onset. 

**Table 4:** Phantosmia etiology in patients with phantosmia.

Primary etiology	CUP ^†^	BPAS ^†^
*Idiopathic*	32 ^w^	10
*Other*	8 ^x1^	78 ^w2^
Head injury	1 ^w^	40
PIHH ^‡^	3 ^w^	24
Infectious mononucleosis	2	0
Pregnancy (during/post partum)	2	1
Allergic rhinitis	0	4
Post nasal surgery	0	3
Post stroke	0	2
Idiopathic hyposmia	0	0
Idiopathic aliosmia ^φ^	0	2

**^†^** Patient Number; ^‡^ Post influenza-like hyposmia and hypogeusia; ^φ^ Distorted smell in presence of external vapor; *CUP vs. BPAS*: ^w^ *p* < 0.001, *X*^2^ (idiopathic *vs*. other); PIHH; Infectious mononucleosis; *CUP*: ^x1^ *p* < 0.01, *X*^2^ (idiopathic *vs*. other); *BPAS*: ^w2^ *p* < 0.001, *X*^2^ (idiopathic *vs*. other).

Phantosmia was initially present only in right naris in 60% (24 of 40) (Table 2). In 40% phantosmia was initially present only in left naris (16 of 40 patients). Of patients with right naris presentation 54% were right handed 46% were left handed (Table 2). Of patients with left naris presentation, 61% were right handed, 39% were left handed (Table 2). There were no differences in phantosmia naris location in left or right handed patients. There were no gender related differences with respect to handedness or initial side of phantosmia. Nineteen percent of patients were left handed, a greater number than the 10%–15% usually considered to be left handed in the general population [168,169]. 

Phantosmia occurred initially spontaneously without any stimulation in 36 patients (90%) (Table 5). Phantosmia occurred initially after an endogenous stimulatory event in four patients (9.5%); in two (5%) after they blew their nose, in one after she sneezed and in one after she took oral adrenal corticosteroids for treatment of a systemic reaction to a bee sting.

No patient spontaneously complained of or recognized any loss of taste or smell acuity either before or after phantosmia onset but each complained that their smell and taste function was changed (*i.e.*, distorted) in presence of phantosmia. However, using specific psychophysical tests (gustometry and olfactometry) in the absence of phantosmia 8 of 40 (20%) had a mild smell loss (Type II or III hyposmia) and 9 (22.5%), a mild taste loss (Type II or III hypogeusia [102,105] (Table 5)). Each patient with hyposmia detected and recognized all odorants but seven of eight (those with Type II hyposmia [102,105,160]) had DT and/or RT greater than those of normal subjects; one patient had Type III hyposmia. Any smell deficit was birhinal. Each patient with hypogeusia detected and recognized all tastants but eight of these nine had Type II hypogeusia [102,105,161] (Table 6) and one patient had Type III hypogeusia [105,161]. Any taste deficit was orally global.

While each patient developed phantosmia as an independent symptom which did not subjectively influence taste or smell acuity, eventually as phantosmia became more intense and occurred more frequently, each complained that the odor interfered with ability to eat and enjoy food with eating and drinking intolerable due to this overriding obnoxious sensation. Only in this sense did patients report that phantosmia affected smell or taste function.

**Table 5:** Taste and smell acuity in patients with phantosmia.

	CUP ^†^	BPAS ^†^
*Taste acuity*		
Normal	31 ^w1^	17
Abnormal	9	71 ^w2^
Ageusia	0	0
I	0	0
II	8	61
III	1	10
*Smell acuity*		
Normal	32 ^w1^	12
Abnormal	8 ^w^	76 ^w2^
Unilateral	0	0
Bilateral	8 ^w^	76 ^w2^
Anosmia	0	1
I	0	20
II	7	42
III	1	13

**^†^** Patient Number; *CUP vs. BPAS*: ^w^ *p* < 0.001, *X*^2^ (taste acuity, abnormal; smell acuity, bilateral, abnormal); *CUP*: ^w1^ *p* < 0.001, *X*^2^ (taste acuity, normal *vs*. abnormal; smell acuity, normal *vs*. abnormal); *BPAS*: ^w2^ *p* < 0.001, *X*^2^ (taste acuity, normal *vs*. abnormal; smell acuity, normal *vs*. abnormal; unilateral *vs*. bilateral).

**Table 6:** Final naris localization in patients with phantosmia with respect to odor character.

	CUP ^†^ Final naris odor localization			BPAS ^†^ Final naris odor localization		
Odor character	Right	Left	Birhinal	Right	Left	Birhinal
Cacosmic (putrid)	7 ^4^	9 ^1^	4 ^3^	0	0	20
Mixed (cacosmic and torquosmic)	7	1	3	0	0	1
Torquosmic (burned, metallic, *etc*.)	1	0	8	0	0	67

**^†^** Patient Number; ^1^ *p* < 0.01, ANOVA, left *vs*. bilateral, cacosmic *vs*. mixed (CUP), left *vs*. bilateral, cacosmic *vs*. torquosmic (CUP), right *vs*. bilateral, cacosmic *vs*. torquosmic (CUP); ^3^ *p* < 0.02, ANOVA, bilateral, cacosmic *vs*. mixed (CUP, BPAS); ^4^ *p* < 0.05, ANOVA, right *vs*. left, cacosmic *vs*. mixed (CUP).

An associated history of some prior clinical nasal pathology was reported by 15 patients (37.5%). Five had a history of chronic allergic rhinitis with hayfever and two of these had chronic sinusitis. Two of these five patients also had a history of nasal polyps in the naris of the phantosmia prior to phantosmia onset although polyps were not present at time of presentation at The Clinic. Ten patients, prior to their visit at The Clinic, but after phantosmia onset, had surgical procedures in the naris on the side of phantosmia in an apparent attempt to relieve phantosmia; this included submucous resection (7 patients) and unilateral Caldwell-Luc operation (three patients). No surgical procedure influenced phantosmia onset, offset or intensity.

Most patients (85% (34 of 40)) reported an associated recurrent, cyclic, episodic orally global phantom taste sensation (cyclic phantageusia) time-locked with phantosmia. Cacogeusic phantageusia was present initially in over 90% of patients and was similar to phantosmia character. Because most considered phantosmia to permeate their entire oronasopharyngeal cavity, albeit it began in one naris, phantageusia might be considered an extension of phantosmia so that it is difficult to insure that this symptom was independent and related only to taste, per se, rather than a phantosmia manifestation. In 10%, cyclic phantageusia was of a torquegeusic character, different from that of phantosmia (*i.e.*, cacogeusic) albeit still time-locked to appearance and disappearance of phantosmia. In these latter patients reports of this symptom may be considered an indicator of phantageusia *per se*. Just as phantosmia was initiated by cough, sneeze, blowing of nose, *etc*., or inhibited by sleep or Valsalva, so phantageusia was also initiated or inhibited.

In some patients intermittent cyclic nature of phantosmia, once it attained a specific level of intensity, frequency, and duration, persisted in this pattern for y, occurring as often as 5–20 times daily on some d, being absent on others, but always recurring in an episodic manner in the same manner in which it initially occurred (Table 3). Its onset did not have any specific relationship to menses in women. The episodic pattern was interrupted following Valsalva performance or daytime nap for periods varying from minutes to hours but following this inhibition the cyclic, episodic pattern eventually resumed. In these patients after Valsalva or nap, inhibition of phantosmia lasted for minutes–hours, but then it either returned spontaneously or was reinitiated by cough, sneeze, nasal discharge, blowing of nose, *etc*., as noted above. Because phantosmia could be initiated or triggered by these activities some patients attempted to suppress all activities that might initiate phantosmia causing them to limit self-expression, social and physical activity and was eventually associated with a degree of depression. Phantosmia intensity usually reached a specific level at which it remained albeit to some extent intensity waxed and waned.

No patient reported headache or facial fullness as part of the onset or offset of this initial phase of hallucinatory activity or during any part of the post symptom period.

Second Phase: In some patients, usually after a period of years, phantosmia did not spontaneously disappear after onset but became relatively persistent. Thus, once phantosmia appeared in the morning or afternoon it persisted until sleep or Valsalva relieved it. Among patients in whom phantosmia became more permanent most found it progressively more difficult to inhibit it with either sleep or Valsalva. 

During this second phase there was some change in phantosmia character. In half the patients (20 of 40) phantosmia in this second phase persisted as cacosmic (Table 2 and Table 6), in 22% (9 of 40) it persisted as torquosmic but in the remainder (11 patients, 28%) it became mixed (both cacosmic and torquosmic). 

Phantosmia persisted unirhinally in 62.5% (Table 2, Table 3 and Table 6). In 27.5% (11 of 40 patients) phantosmia was initially present only in left naris and it remained localized on that side (Table 2 and Table 6); in 40% (16 of 40 patients) phantosmia was present initially in right naris and it remained localized on that side. In 37.5%, phantosmia became birhinal. Among patients with unirhinal cacosmic phantosmia, four (10%) developed a birhinal component; among patients with unirhinal torquosmic phantosmia eight (20%) developed a birhinal component and among patients with a mixed unirhinal phantosmia three (7.5%) developed a birhinal component (Table 2 and Table 6). These results indicate that patients with unirhinal torquosmic phantosmia were significantly more likely to develop a birhinal component than were patients with either unirhinal mixed or unirhinal cacosmic phantosmia (*p* < 0.01, *X*^2^). This difference may be of value in differentiating patients with these phantosmic characteristics.

All patients related a consistent history that the side of initial phantosmia retained its initial character, although frequency and intensity increased. However if phantosmia occurred in the opposite naris it rarely appeared alone but usually in both nares simultaneously. When this occurred phantosmia in the initial naris retained its initial character and intensity but the naris to which phantosmia extended was usually of less intensity and frequency and was usually of a different character than initial unirhinal phantosmia; this was commonly of a torquosmic type (v.s.). In these patients it was still most common to perceive phantosmia in one naris and only on occasion would phantosmia appear in both nares simultaneously. However, when phantosmia appeared birhinally patients reported that it was more overwhelmingly obnoxious and their ability to disregard it was even more impaired than it was when it appeared unirhinally due to odor character and intensity.

One patient developed unique sequelae. She developed painful optic neuritis in the left eye 10 years after phantosmia onset associated with optic atrophy, which was unresponsive to systemic adrenal corticosteroid therapy. Visual acuity progressively decreased in this eye to only light-dark perception with absence of finger counting and eventually decreased in right eye to only finger counting. This patient also had varicosities in venous plexi in both retinae, which became engorged during Valsalva used to relieve phantosmia. Neither oligoclonal immunoglobulin pattern in cerebral spinal fluid (CSF) nor periventricular white matter lesions on MRI were present. 

No patient reported headache or facial fullness either as part of the onset or offset of this initial or second phase of this syndrome or during any part of the post symptom period.

BPAS: Forty-seven women and 41 men had BPAS (Table 1). Patients’ age varied from 1 to 82 years (46 ± 2 years, mean ± SEM (Table 1). Three patients developed symptoms prior to puberty. All were Caucasian. Symptoms were experienced one week to 10 years (mean 1.8 years) prior to presentation at The Clinic. Birhinal phantosmia followed hyposmia onset by 1 week–6 months in 70 patients (80%); it occurred simultaneously with onset of either hyposmia or other symptoms in 18 (20%) (Table 1). The most common initial symptom was hyposmia (in 86%). Phantosmia was always birhinal as opposed to the unirhinal character in CUP (Table 1). It was usually torquosmic as opposed to the primarily cacosmic character in CUP (Table 1). Mean age in BPAS was significantly older than in CUP (*p* < 0.01, *t* test) (Table 1). In BPAS there was a similar number of men and women whereas in CUP women outnumbered men by 3:1, a significant different gender ratio (*p* < 0.05, *X*^2^). No prior or subsequent clinical motor activity occurred in any patient.

Birhinal hyposmia was the initial presenting clinical complaint in 86% (as opposed to initial presenting complaint of unirhinal phantosmia in 100% of patients with CUP (Table 1)); the remainder of BPAS patients had other associated smell and/or taste dysfunction including global hypogeusia and/or global aliageusia, aliosmia and phantageusia (Table 5 (v.s.)). BPAS patients recognized their impaired smell acuity as opposed to CUP patients who generally did not have this symptom (Table 1 and Table 6); 28% of BPAS patients exhibited an inability to recognize any vapor at any concentration (*i.e.*, absent recognition thresholds for any odorant, Type I hyposmia [102,105,130]), whereas no CUP patient exhibited Type I hyposmia (Table 5).

Phantosmia developed as a sequela of an illness, secondary to hyposmia in 86% of patients with BPAS (Table 4 (v.s.)). The most common cause was head injury (post-concussive syndrome, 40 patients, 45% of total group [102,105]), followed by an influenza-type viral infection (post influenza-like hyposmia and hypogeusia (PIHH), 24 patients, 27% of total group [102,105,157] (Table 4)). As opposed to CUP in whom unirhinal phantosmia was the first and only symptom, phantosmia in BPAS usually appeared later, days, weeks or even months after appearance of hyposmia or other symptoms (Table 1); infrequently it appeared with onset of their associated symptoms. No patient experienced a seizure with motor activity before or after phantosmia onset despite the fact that 45% experienced head injury as the cause of their hyposmia and several of these patients experienced prograde and retrograde amnesia, loss of consciousness and skull fracture with their injury. After phantosmia terminated no physiological carry-over (*i.e.*, no post-ictal type symptoms) were present similar to CUP.

Phantosmia was always birhinal (Table 1 and Table 2) and it always retained this character. It was initially intermittent or persistent but neither cyclic nor episodic, usually lasting from seconds to minutes to hours (Table 3); in some patients phantosmia was persistent and was present for days to weeks. In contrast to CUP, phantosmia in BPAS usually remained intermittent or persistent and, rather than increase in frequency and intensity, as in CUP; overtime it either remained the same or, more commonly, decreased in both frequency and intensity and in some patients disappeared although hyposmia always persisted. Phantosmia onset and offset usually occurred in an irregular, unpatterned, unpredictable manner. Phantosmia was not elicited by any physiological maneuver, usually occurring spontaneously; however, in a few patients, a strong, common ambient odor triggered phantosmia (*i.e.*, allodynosmia) with no distortion associated with the ambient odor per se (*i.e.*, no aliosmia). If phantosmia occurred after exposure to an ambient odor, after external odor removal, phantosmia either disappeared immediately or pursued its usual course and then disappeared as opposed to phantosmia persistence in CUP. No physiological maneuver initiated, diminished or abolished phantosmia. Phantosmia usually retained its initial character, which did not vary over time (v.s.).

Only on occasion did BPAS patients experience phantageusia (in 22 patients or 25% of the total group). This symptom was usually independent of phantosmia, was of a different character than phantosmia and of much less severity than phantosmia. When it occurred, phantageusia was orally global but usually transient and unrelated to any physiological stimulus. Allodyngeusia rarely occurred in BPAS.

Phantosmia character was significantly more frequently torquosmic (70 patients, 80% of the total group) than cacosmic (in 17 patients or 21%) or mixed (1 patient, 1% of the total group) (Table 1 and Table 2), in contrast to CUP patients in whom phantosmia were more frequently cacosmic (ANOVA, *p* < 0.01, *t* test).

Two patients had psychiatric diagnoses at their first visit to The Clinic; one had an obsessive-compulsive disorder and one had clinical depression. Both were under psychiatric care and taking anxiolytic drugs at that time; this treatment did not alter character or frequency of phantosmia or hyposmia.

No patient experienced any sensation in nose, taste in mouth or unusual feelings of any type prior to phantosmia onset; it occurred spontaneously after hyposmia onset.

No change in emotional state, willful behavior or physiological maneuver initiated or altered phantosmia.

No patient reported headache or facial fullness either as part of the onset or offset of the hallucinatory activity or during any part of the post symptom period.

### 3.2. Physical Examination of Head and Neck

CUP: No patient had any observable change from normal in nasopharyngeal cavity, mouth or neck. Uvular and palatal reflexes were normal. Each patient had both thick and thin nasal mucus in both nasal cavities. Nasal mucous membranes in both nares were of normal character and turgor. Nasal breathing was not altered in any patient.

BPAS: Twelve of 88 patients (14%) exhibited observable changes from normal in their nasal cavity; these were in 10 patients with PIHH and two patients with allergic rhinitis. In patients with PIHH there was observable thinning of nasal mucous membranes with absence of thick nasal mucus and increased nasal airways patency, as previously described [102,105,157]. In patients with allergic rhinitis there was modest edema of nasal mucous membranes birhinally, increased nasal congestion and slightly decreased nasal airways patency [102,105,158]. Changes did not subjectively restrict nasal air flow in any patient and each stated that nasal breathing was unchanged either after loss of olfactory acuity and/or onset of birhinal phantosmia. No patient exhibited nasal polyposis and none exhibited polypoid degeneration of nasal mucous membranes. No abnormalities were found in examination of mouth or neck in any patient.

### 3.3. Neurological Examination

CUP: Except for the presence of phantosmia the clinical neurological examination was within normal limits in each patient.

BPAS: Except for the presence of phantosmia and hyposmia the clinical neurological examination was within normal limits in each patient.

### 3.4. EEG Changes

CUP: EEG abnormalities were observed in 19 of 40 patients (47.5%) (Table 7 and Table 8). Abnormalities were found in temporal lobe in 13 patients, in fronto-temporal region in two, in frontal region in three and location was indeterminate in one. EEG slowing was observed in two patients and was diffuse. Seventeen patients (89%) exhibited sharp waves, three exhibited spike discharges. Photostimulation elicited abnormalities in two patients but changes were only exacerbations of already existing EEG abnormalities, both left temporal sharp wave discharges. Hyperventilation elicited abnormalities in two records, one in which photostimulation also exacerbated previously observed sharp waves in left temporal lobe.

Of 15 right handed patients with EEG abnormalities, eight exhibited left sided changes, six, bilateral and one with right sided changes (Table 8). Of six patients with bilateral abnormalities, three exhibited changes greater on left than right. Of the five left handed patients with EEG abnormalities, two exhibited right sided changes, two exhibited left sided changes and one exhibited bilateral changes with changes greater on left side than right.

**Table 7:** EEG characteristics in patients with phantosmia.

Condition	CUP ^†^	BPAS ^†^
*EEG abnormality present*		
No	22 ^w^	76
Yes	18	12
Men	5	5
Women	13	7
*Abnormality type*		
Slowing	2 ^z1^	6
Sharp waves	16 ^z^	6
*Abnormality site*		
Localized	17 ^z, w1^	8
Generalized	1	4
*Similar side phantosmia/EEG abnormality*		
Yes	4 ^z1^	--
No	14	--
*“Opposite side” phantosmia/EEG abnormality*		
Yes	6	--
No	12	--

**^†^** Patient Number; *CUP vs. BPAS*: ^w^ *p* < 0.001, *X*^2^ (EEG abnormality, no *vs*. yes); ^z^ *p* < 0.05, *X*^2^, (sharp waves present, localized EEG changes); *CUP*: ^w1^ *p* < 0.001, *X*^2^ (localized *vs*. generalized); ^z1^ *p* < 0.05, *X*^2^ (slowing *vs*. sharp waves; EEG abnormality, not on similar side EEG abnormality).

**Table 8:** EEG characteristics in patients with phantosmia.

Condition	CUP ^†^	BPAS ^†^
*Skilled Hand*		
Right (R)	37 ^x^ (78)	85 (97)
Left (L)	9 ^x^ (22)	3 (3)
*Final naris side of phantosmia*		
L Sided Phantom	13	0
R Sided Phantom	15	0
Bilateral phantom	12 ^w^	88
*Handedness vs. Final side of phantosmia*		
R Handed, L Sided Phantom	12 (30)	0 (0)
R Handed, R Sided Phantom	9 (22)	0 (2)
R Handed, Bilateral Phantom	10 (25)	85 ^w2^ (97)
L Handed, R Sided Phantom	2 (5)	0 (1)
L Handed, L Sided Phantom	3 (8)	0 (2)
L Handed, Bilateral Phantom	4 (10)	3 (3)
*Handedness vs. Side of EEG abnormality*		
R Handed, L Sided EEG	8 (44)	3 (25)
R Handed, R Sided EEG	1 (6)	1 (8)
R Handed, Bilateral EEG	4 (22)	8 (67)
L Handed, R Sided EEG	1 (6)	0
L Handed, L Sided EEG	2 (11)	0
L Handed, Bilateral EEG	2 (11)	0
*Final side of phantosmia vs. Side of EEG abnormality*		
R Side, L EEG	4 (22)	0
R Side, R EEG	0 (0)	0
R Side, Bilateral EEG	3 (17)	0
L Side, R EEG	2 (11)	0
L Side, L EEG	3 (17)	0
L Side, Bilateral EEG	2 (11)	0
Bilateral, L EEG	3 (17)	0
Bilateral, R EEG	0 (0)	0
Bilateral, Bilateral EEG	1 (5)	0

**^†^** Patient Number; ( ) Percent; *CUP vs. BPAS*: ^w^ *p* < 0.001, *X*^2^ (bilateral phantom, final side); ^x^ *p* < 0.01, *X*^2^ (R skilled hand, L skilled hand); *BPAS*: ^w2^ *p* < 0.001, *X*^2^ (bilateral phantom *vs*. L or R sided phantom).

Stage II sleep was obtained in 22 patients (55%) demonstrated by sleep spindles on EEG record; in only one patient were EEG changes not observed during the waking EEG observed during sleep. 

Phantosmia was elicited and specifically noted during EEG recording in 24 patients (60%); no abnormality not previously observed was elicited with phantosmia. Mean duration of phantosmia was 21.4 min (range 1–120 min). In some patients phantosmia was not present at beginning of EEG but was elicited during the record by deep, rapid, repeated nasal sniffs or hyperventilation; in other patients phantosmia was present at initiation of EEG record and was terminated by a Valsalva maneuver. No matter the sequence, no change in EEG over that observed before initiation or termination of phantosmia was observed.

Nasopharyngeal leads revealed no additional abnormalities over those observed without use of these leads.

BPAS: EEG abnormalities were observed in 12 of 88 patients (14%) a significantly smaller percentage than in CUP (Table 7) (*p* < 0.01, *X*^2^, *F* test). Of EEG changes observed, six showed slowing and six showed sharp waves (all among the 40 who had head injury), also significantly less than in CUP (Table 7) (*p* < 0.05, *X*^2^, *F* test). EEG changes were localized in eight patients and generalized in four, also significantly different from increased localization of EEG changes observed in CUP (*p* < 0.05, *X*^2^, *F* test). Phantosmia was reported present during EEG in 15 patients lasting from sec to the entire EEG; this symptom was not and could not be elicited by any behavioral or physiological maneuver in any patient who did not have the symptom at initiation of EEG. Phantosmia presence was not associated with any specific abnormality observed in EEG record and did not distinguish EEG records of these patients from others with BPAS. 

Stage II sleep was obtained in 47 patients (53%) demonstrated by sleep spindles on EEG record; no EEG changes not observed during wakefulness were observed during sleep. Nasopharyneal leads revealed no additional abnormalities over those observed without these leads.

### 3.5. Neuroimaging Studies

CUP: Some abnormality was observed in 11 patients (27.5% (Table 9); these were CT brain scan changes in six patients (bilateral parietal parasagital encephalomalacia (one patient), mild frontal atrophy (one patient), undefined soft tissue density (one patient), calcification within the sella turcica (one patient) mucoperiosteal thickening of ethmoid and maxillary sinuses, bilaterally (one patient) and a cystic formation in the left maxillary antrum (one patient)). In one patient a solitary plaque was found in central white matter on MRI. In the three women who underwent cerebral angiography one had bilateral orbital vein varicosities with right superior orbital vein thrombosis whereas no abnormalities were observed in the other two. Abnormalities on skull X-rays were sella turcica calcification and mucoperiosteal sinus thickening also observed on CT brain scan. Abnormalities on sinus x-rays were mucoperiosteal thickening of the maxillary sinuses, bilaterally, in two patients. 

**Table 9:** Imaging studies in patients with phantosmia.

Condition	CUP ^†^	BPAS ^†^
*CAT scan—brain*	28 ^z^	55
Abnormal studies	6	16
Normal studies	22	39
*MRI brain*	4	2
Abnormal studies	1	0
Normal studies	3	2
*Tc99^m^ brain scan*	0	6
Abnormal studies	0	0
Normal studies	0	6
*Skull X-ray*	20	6
Abnormal studies	2	1
Normal studies	18	5
*Sinus X-ray*	12	14
Abnormal studies	2	4
Normal studies	10	10
*Total patients with radiographic studies*	40	83
Abnormal studies	11	21
Normal studies	29	62

**^†^** Patient Number; *CUP vs. BPAS*: ^z^ *p* < 0.05, *X*^2^ (abnormal CAT scan).

BPAS: Abnormalities were observed in 21 patients (24% (Table 9)), not significantly different from that observed in CUP. Abnormalities on brain CT scans included seven with encephalomalacia with or without atrophy, four with specific frontal or temporal atrophic changes, one with a subdural hematoma, one with a subarachnoid hemorrhage, one with an occipital skull fracture (all 14 abnormalities among the 40 patients with a prior history of head injury) and two with bilateral maxillary sinusitis (among the four patients with allergic rhinitis). The abnormality on skull X-ray was a basilar skull fracture in one patient at time of prior head trauma. Abnormalities on sinus-X rays were sinusitis of maxillary antra (unilateral or bilateral) in four patients (two among the four patients with allergic rhinitis and two among the three patients post nasal surgery). 

In a recent study of brain MRI scans in 1000 asymptomatic nominally normal volunteers 18% showed abnormal results with 13.2% exhibiting what was interpreted radiographically as paranasal sinusitis [170]. In other studies of this type paranasal sinusitis was found in 42%–49% of supposedly normal subjects [170,171,172,173].

### 3.6. fMRI Studies

CUP: Brain activation to phantosmia memory was extremely robust and quantitatively greater than to any other olfactory stimulus (e.g., smell of any vapor, including absolute pyridine, an extremely pungent, unpleasant odor [104]). Although phantosmia was unirhinal, brain activation to phantosmia and phantosmia memory was bihemispheric. CNS activation was not related to patient handedness or side of phantosmia although a suggestion of preponderance of right as opposed to left hemispheric activation was apparent [174]. After forced nasal inhalation and exhalation initiated unirhinal phantosmia, increased bihemispheric CNS activation occurred [104]. After Valsalva inhibited unirhinal phantosmia increased bihemispheric CNS activation also occurred [104]. Regional CNS activation was sensory specific, greatest in anterior frontal and temporal cortex [104], but prominent activation was measured in cingulate and in striatum.

BPAS: Brain activation to phantosmia memory was also robust and quantitatively greater than to any olfactory stimulus previously presented [162]. Regional CNS activation was sensory specific, greatest in the anterior frontal and temporal cortex with little or no activation in striatum [162]. No physiological maneuver increased and inhibited CNS activation in patients with BPAS.

### 3.7. MRS Studies

CUP: Results of preliminary studies indicated ratio of GABA: creatine in occipital cortex was not different between the two patients studied and the normal volunteers (patients, mean, 0.22, normals, 0.18 ± 0.03 (Mean ± SEM)). Ratio of GABA: creatine was lower in cingulate and right insula cortex in patients compared to normal volunteers (patients, mean, cingulate, 0.12, right insula, 0.10; normals, cingulate, 0.21 ± 0.05, right insula, 0.22 ± 0.06). Results indicate mean ratio of GABA: creatine in cingulate cortex in patients was 57% that in normal volunteers, mean ratio in right insula in patients was 45% that in normals. 

BPAS: Similar regional changes in GABA: creatine to those found in CUP were also found in BPAS. In occipital cortex, ratio of GABA: creatine was similar in patients and normals (patients, 0.17 ± 0.04, normals, 0.18 ± 0.03). In patients, in cingulate and right insular cortex, ratio of GABA: creatine were significantly lower than in normals (cingulate, patients, 0.11 ± 0.007, normals, 0.21 ± 0.05, *p* < 0.01 *t* test; right insula, patients, 0.14 ± 0.03, normals, 0.22 ± 0.06, *p* < 0.01, *t* test).

### 3.8. CO_2_ Inhalation Studies

CUP: Following CO_2_ inhalation, inhibition of phantosmia was reported by seven of the 10 patients (six women, one man) to whom it was administered. CO_2_ inhalation was difficult to maintain due to its abrasive character. After initial CO_2_ inhalation phantosmia inhibition persisted from periods of one-48 h (five women, one man) to 7 days (one woman). The longer the patient tolerated CO_2_ exposure the longer phantosmia inhibition. During this inhibition period no physiological maneuver which previously initiated phantosmia (e.g., sneezing, crying, blowing the nose, hyperventilation) initiated phantosmia despite repeated efforts to perform these maneuvers. At termination of this refractory period phantosmia either returned spontaneously or was initiated by a maneuver which commonly initiated it prior to CO_2_ inhalation. In the woman in whom phantosmia was inhibited for 7 days, phantosmia returned after she blew her nose. CO_2_ inhalation was then repeated on two additional occasions over a period of five weeks; after each inhalation her phantosmia was inhibited, albeit length of inhibition period decreased after each CO_2_ inhalation. In four of the 10 patients (one woman, three men) no effect of CO_2_ inhalation could be convincingly demonstrated. Administration of CO_2_ by facial mask may play a role in delivery of this agent since, by analogy, delivery of oxygen through nasal prongs did not influence incidence of post-operative wound infections [175,176] whereas delivery through facial mask decreased wound infection incidence [177] presumably through increased arterial oxygen tension and subcuteneous tissue oxygen tension [178]. No change in smell acuity occurred following this procedure. 

BPAS: CO_2_ inhalation was not used in any patient.

### 3.9. Intranasal Cocaine Administration

CUP: Unirhinal intranasal cocainization initiated immediate cessation of phantosmia in each patient in whom it was used. This effect persisted for 2 h in one patient and for 8 h in the other. During this inhibitory period no physiological maneuver, which previously initiated phantosmia, did so despite repeated attempts to initiate it. Whereas prior to this intervention both exhibited normal smell function Type I hyposmia was induced in the cocainized naris subsequent to this procedure (determined by absent RT and diminished DT for all odorants using unirhinal testing) and its time course roughly followed onset and offset of phantosmia. Phantosmia returned either spontaneously (one patient) or after blowing the nose (one patient), which terminated this inhibitory period. Subsequent to this intervention with phantosmia return smell function also returned to normal in each patient.

BPAS: Birhinal intranasal cocainization initiated immediate cessation of phantosmia in the patient in whom it was used. This effect persisted for approximately 10 days after which time it returned in a pattern similar to that noted prior to this procedure. This patient exhibited Type I hyposmia prior to nasal cocainization and it did not change after intranasal cocainization. 

### 3.10. rTMS Studies

CUP: No patient reported any change in phantosmia intensity or character after shoulder or neck stimulation at 40% intensity or after stimulation to any skull region at 20% intensity. Each patient reported a significant initial decrease (10%–30%) in phantosmia after stimulation at 60% maximal intensity in left temporoparietal region with no further change after stimulation in any other region. Repeat stimulation (one to six applications) in this region induced a continued decrease to a stable inhibition level about 50% of pre stimulation phantosmia which persisted for 3 h in one patient and 20 days in the other. During this inhibitory period no physiological maneuver (e.g., nose blowing, hyperventilation) induced phantosmia. In the patient in whom inhibition persisted for three hours phantosmia intensity increased to prestimulation levels after this period following hyperventilation. In the patient in whom phantosmia inhibition persisted for 20 days phantosmia intensity increased after this period but only to about 60% of pre stimulation intensity after nose blowing and did not increase further in intensity with any physiological maneuver. Significant increases in carbonic anhydrase I, II and VI occurred in blood plasma, erythrocytes and saliva, respectively, and in plasma, erythrocytes and saliva zinc and copper measured after rTMS consistent with the phantosmia decrease in each patient [167]. Repeat stimulation was not performed in either patient.

BPAS: No patient reported any change in phantosmia intensity or character after stimulation to shoulder or neck or after stimulation in any skull region at 20% intensity. Each patient reported a significant initial decrease in phantosmia after stimulation at 60% maximal intensity [165,166]. Three reported an initial decrease in phantosmia intensity after stimulation in left temperoparietal region with no further change after stimulation in any other region. Initial response was usually a decrease of 20%–50%. Repeat stimulation (one to six applications) in this region induced a report of total absence of phantosmia (an intensity decrease of 50%–100%). Associated with this decrease changes in carbonic anhydrase I, II and VI and in zinc and copper similar to those measured in CUP were also reported [167].

The decrease in phantosmia was as long lasting as reports were obtained in two patients (both women (*i.e.*, after 24 months)) and lasted two mo (one man) in the other patient; repeat stimulation in this latter patient induced a four month phantosmia remission after a spontaneous return to about 50% of its prior intensity. After another spontaneous return to about 40% of prior phantosmia intensity a further stimulation resulted in phantosmia inhibition that has lasted for over one year. One patient (one man) reported an initial decrease (~40%) in phantosmia after initial stimulation in right temporoparietal region without further change in stimulation in any other region. Repeat right temporoparietal stimulation resulted in complete inhibition of phantosmia (100% decrease), which has lasted as long as records were obtained (>48 months).

### 3.11. Evaluation and Treatment Prior to This Study

CUP: After phantosmia onset each patient sought assistance from some medical caregiver to attempt to evaluate and alleviate the symptom [102,105]. Each treated patient was evaluated by an average of four practitioners prior to evaluation and treatment at The Taste and Smell Clinic in Washington, DC, USA. Initially patients received little or no encouragement or treatment from their local medical consultants. Subsequently, 28 of the 40 (70%) received at least one form of treatment for periods of one wk to four mo prior to their visit to The Clinic (Table 10). Thirteen (32.5%) were told their phantosmia was psychosomatic and were referred for psychiatric evaluation and treatment. Each of these patients underwent psychiatric treatment and were given anxiolytic agents which did not alter phantosmia (Table 10). Twelve (30%) received antidepressants, (including dibenzocycloheptadines (eight patients), benzodiazepines (two patients), phenothiazines (one patient) and other tricyclic antidepressants (one patient)) which improved mood in four patients but did not significantly alter phantosmia in any patient. Thirty patients (75%) were referred to a neurologist. After obtaining an EEG, the results of which are not available, each was considered to have some type of seizure disorder and 24 (60%) were given an anticonvulsant either singly (usually phenytoin) or in combination with another anticonvulsant agent (Table 10); phantosmia was not permanently altered by any of these drugs although one patient reported temporary relief from phenobarbital treatment. Some patients went to otolaryngologists. Thirteen (32.5%) were given antibiotics; five of these were also given antihistamines and eight were also given either systemic adrenocorticosteroids or intranasal steroid sprays; no phantosmia change resulted from these therapies. Three were treated with allergic immunotherapy without benefit. Three received intranasal installation of 10% cocaine; in two patients phantosmia was inhibited for 24 h and then returned; in one patient phantosmia was inhibited for four mo before returning. Ten patients underwent surgical procedures involving the affected naris in an attempt to relieve phantosmia; seven underwent nasal submucous resections and three underwent Caldwell-Luc procedures. No procedure inhibited phantosmia. Eleven were given exogenous oral zinc ion and two were treated with massive doses of vitamins (vitamin A, 50,000 units daily (one patient), B complex vitamins (one patient)) without change in phantosmia. Other attempts to evaluate and control phantosmia included various forms of alternative medical practice including medicinal herbs, nutritional supplements, hypnosis and acupuncture. One patient reported phantosmia inhibition with daily smoking of THC (v.s.). Two patients received thioridazine with one noting diminution in phantosmia intensity after the drug was administered for one mo whereas the other noted no benefit after one mo of treatment.

BPAS: Each patient repeatedly sought medical assistance primarily for treatment of loss of smell and flavor acuity, their major complaints [102,105], although not all patients received treatment (Table 10). While not unconcerned with their phantosmia most physicians considered this a lesser problem than loss of ability to smell and taste (obtain flavor) from food. Patients were evaluated and treated by an average of four practitioners prior to their first visit to The Clinic. Seventy-one patients (81%) received at least one form of treatment for two weeks to six months prior to their visit to The Clinic (Table 10). Most patients, after receiving little assistance from their local medical consultant, were referred to an otolaryngologist who usually considered their symptoms a local nasal disease. Fifty-five (62.5%) were treated with antibiotics, 23 (26%) with antihistamines and 22 (25%) with adrenocorticosteroids (systemic therapy (19 patients), intranasal sprays or injections (12 patients)); none of these treatments changed phantosmia. Oxymetazoline nasal spray was used in six patients without alteration of phantosmia. Eleven were treated with allergic immunotherapy without benefit. Surgical procedures were attempted to correct what was considered clinically to be either nasal anatomical problems (e.g., deviated nasal septum) or as treatment for hyposmia and/or phantosmia. Eight patients underwent submucous nasal resection and three underwent birhinal Caldwell-Luc procedures. None of these procedures was effective in relieving the patients’ symptoms. When this approach failed to restore smell and flavor acuity patients went to a variety of other physicians for assistance. Nine went to neurologists (significantly fewer than those with CUP (*p* < 0.01, *X*^2^)) who treated them with anticonvulsant medication either singly or in some drug combination (Table 10); this therapy was ineffective in relieving phantosmia. Five patients were referred to psychiatrists; each was treated with antidepressants (dibenzocycloheptadines (two patients), benzodiazepines (one patient) and other tricyclic antidepressants (two patients)), significantly fewer than those with CUP. As in CUP no patient noted any change in phantosmia with this therapy. Thirty (34%) were treated with exogenous oral zinc ion and 10 were treated with massive doses of vitamins (vitamin A, 50,000 units daily (five patients) B complex vitamins (five patients)) without any change in phantosmia. Some patients were evaluated and treated by alternative healthcare practitioners including nutritionists, acupuncturists and hypnotists without change in phantosmia. Neurologists and neurosurgeons who initially evaluated most patients with head injury commonly considered the associated loss of smell and flavor perception related to irreversible anatomical damage to the olfactory nerves and/or CNS and usually told patients that there was no possible treatment for their loss of acuity [179] albeit some instituted therapy for phantosmia and/or phantageusia with antiepileptic agents if these symptoms were prominent enough that they considered them consistent with a seizure disorder. If phantosmia and/or phantageusia still persisted many patients returned to otolaryngologists who again treated their loss of acuity as a local nasal disease with agents noted above, again without success. 

**Table 10:** Treatment prior to evaluation at The Taste and Smell Clinic in patients with phantosmia.

Condition	CUP ^†^	BPAS ^†^
*Prior treatment*		
No	13	17
Yes	27	71 ^w^
*Treatment type*		
Anti-convulsants	23 ^w^	9
Phenytoin	12	9
Primidone	5	0
Carbamazepine	3	0
Mebaral	1	3
Phenobarbitol	4	3
Alone	2	0
With another ac	2	3
Anti-depressants	11	5
Anti-histamines	5	23
Adrenocorticosteroids	8	22
Systemic	1	19 ^x^
Intranasal	7	12
Spray	6	10
Local injection	1	2
Other nasal sprays	1	6
Oxymetazoline	1	6
Allergy immunotherapy	3	11
Surgical intervention	2	11
Submucous resection	2	8
Other	0	3
Vitamins/minerals	12	30
Zinc	11	22
Vitamin A	1	5
Other	0	5
Intranasal cocaine	3	0
Thioridazine	2	1

**^†^** Patient Number; *CUP vs. BPAS*: ^w^ *p* < 0.001, *X*^2 ^(yes, prior treatment; anti-convulsant treatment); ^x^ *p* < 0.01, *X*^2^ (systemic adrenocorticosteroids).

## 4. Discussion

### 4.1. Pathophysiological, Peripheral and CNS Components of CUP

A significant peripheral component initiated phantosmia in CUP consistent with peripheral hypersensitivity leading to CNS reorganization and plasticity. Phantosmia was initiated after unirhinal stimulation with normal physiological activities that would not cause any symptom in normal subjects; e.g., blowing the nose, repetitive or excessively intense breathing or exposure to ambient odors initiated phantosmia. These results indicate increased local excitation manifested by peripheral hypersensitivity played a significant role in initiating phantosmia and these effects were localized to olfactory epithelium in one naris. We suggest an endogenous loss of GABAergic function occurred in these patients thereby sensitizing the olfactory system to normal sensory input which initiated inappropriate peripheral responses, extending to CNS as well, as we previously reported [36].

While explanation for this observed peripheral hypersensitivity in CUP may appear to be simplistic it may be similar to task related plasticity [180,181,182,183,184,185] and peripheral hypersensitivity previously observed in dystonia [180,183]. It is well known that repetitive hand or arm movements or extremity overuse initiated and/or increased dystonia [180,181] associated with CNS somatosensory degradation [180], abnormal recruitment of cortical areas involved with control of voluntary movement [186] with impaired and inefficient CNS sensory integration [183]. These phenomena were reproduced in animal models of dystonia in which CNS GABA levels in specific regions were decreased compared to controls [186,187]. Reduction of intracortical GABAergic inhibition in animals by local CNS application of GABA antagonists to motor cortex (e.g., bicuculline, muscimol, lamotrigene) led to hypersensitivity exacerbating dystonic-like movements [186,188,189]. Reduction in putamen and globus pallidus GABA levels were also found in a patient with symptomatic dystonia [190].

Blocking this peripheral component in CUP by a variety of maneuvers always inhibited phantosmia. Unirhinal nasal blockage, unirhinal installation of cocaine, smoking of THC or excision of olfactory epithelium [191] inhibited phantosmia. We suggest peripheral blocking activities in CUP inhibited peripheral hypersensitivity in the unirhinal olfactory epithelium either by inhibiting local stimulus delivery or by increasing local and/or CNS GABAergic activity. These results are similar in several ways to those in task specific focal dystonia [180] in which decreased peripheral input [184] or decreased repetitive activity [192] decreased dystonic movements. For example, limb immobilization [184] inhibited sensory input that might otherwise initiate dystonia [183]. These results support the concept of a balance between peripheral excitation and inhibition in these patients. 

However, these blocking maneuvers (e.g., intranasal cocaine) induced two effects in CUP; *i.e.*, both phantosmia inhibition and hyposmia induction, and it may be difficult to separate these effects functionally or anatomically. Thus, each of these maneuvers that inhibited phantosmia induced loss of smell by directly blocking delivery of vapor to olfactory receptors (nasal plugging) or by inhibiting olfactory activation at the olfactory epithelium (cocaine inhalation). Although changes in olfactory acuity with THC have not been systematically investigated these effects indicate that it is possible to inhibit phantosmia locally by a peripheral effect in one naris. This effect may be due to changes in peripheral GABAergic function or GABA concentration, which putatively increases, since GABA effects, including l-glutamic acid decarboxylase (GAD) metabolism, may relate to its effects in peripheral tissues [193] as well as in CNS. Although data are not clear-cut on this issue THC has been shown to augment inhibitory effects of GABA [194,195,196,197]. Since we demonstrated various drugs which inhibited phantosmia [166,167] also increased CNS GABA function [198,199] it seems reasonable to speculate that these inhibitory effects may occur through increased GABAergic activity which might occur in the periphery as well as in CNS. On the other hand, it might seem reasonable to speculate that if initial effect of any of these maneuvers were to inhibit olfaction then subsequent effects would be deafferentation and functional cortical reorganization with GABA diminution rather than increased GABAergic function similar to induction of phantom limb syndrome. But in CUP we observed both hyposmia and phantosmia inhibition with these blocking maneuvers and we have suggested that this effect was due to increased CNS and presumably increased peripheral GABAergic activity. Mechanisms underlying these effects are complex and current data do not allow a definitive explanation.

Whereas these blocking maneuvers (nasal plugging, nasal cocaine installation) also induced hyposmia in normal subjects, cocaine [200,201,202] and THC [203,204] inhalation may initiate rather than inhibit hallucinations (obviously, normal subjects do not exhibit phantosmia prior to cocaine or THC use). These results suggest a plausible mechanism for these effects in normal subjects although it may be difficult again to separate peripheral from CNS effects. Hallucination induction in normals is consistent with data indicating nasal cocaine inhalation or THC smoking decreased CNS GABA or GABA function [205,206,207,208,209], perhaps also extending to peripheral structures, with subsequent changes in CNS GAD, other moieties, disinhibition of CNS and sensory function [210,211,212] and hallucination onset. It is this decreased GABA we previously hypothesized as consistent in both normals and CUP with onset of hallucinatory activity. However, this hallucinatory behavior in normals occurs after the local effect of cocaine occurs, *i.e.*, after cocaine-induced hyposmia. This loss of sensory input in normal subjects may induce a phenomenon similar to deafferentation hypersensitivity with subsequent CNS dysplasticity as in phantom limb syndrome, a change that may occur rapidly after hyposmia induction. This effect may be similar to rapid reductions in CNS GABA, which occur after deafferentation, as observed after reversible induction of forearm ischemia [213], unilateral labyrinthectomy in rat [214] and in other classic studies [215,216]. There are also slower CNS GABA reductions, long term changes reported in humans after limb amputation [217] after eye enucleation in animals [218,219,220] and after other sensory or activity dependent procedures [221,222] including changes in GAD [222]. 

One possible attempt to understand this putative dichotomous effect of cocaine (and THC) in normals and in CUP may relate to reported biphasic effects of both cocaine and THC on GABAergic activity. Cocaine and THC have been shown to both increase or decrease GABAergic activity by direct CNS effects [206,207,208,222,223,224], through effects on G proteins [220], through effects on GABA receptor function, [222,223,224,225,226,227] through various GABA related metabolic enzymes [205,228,229] mediated by induction of dopamine uptake inhibition [230,231] or through complex chemical kindling mechanisms [232]. These effects for cocaine occur in addition to its well-known local nasal anesthetic effects. By analogy with dystonia, animal models of dystonia have shown that decreased CNS GABA levels resolved after disease disappearance [190,233,234] and treatment with GABAergic drugs restored normal peripheral motor function and CNS GABA levels [232]. Other drugs, which activate CNS GABA (e.g., baclofen, vigabatrin, gabapentin [234,235,236,237,238]) inhibited dystonic movements and we have shown that drugs with similar effects (haloperidol, thioridazine) inhibited phantosmia in CUP [36,104]. 

These mechanisms are supported by our preliminary results with rTMS, which inhibited phantosmia in CUP (v.s.) consistent with induction of increased CNS GABA [165,166]. rTMS in normal humans [239,240], in patients [241] and in animals [242] applied in a similar manner was without any subsequent long term functional physiological effect albeit intracortical inhibitory circuitry was transiently suppressed [243]. Similar inhibitory results were also demonstrated in dystonia [244,245] since rTMS inhibited dystonic movements by inhibiting hypersensitivity with subsequent reorganization of the CNS and correction of maladaptive CNS plasticity. By analogy, these results suggest that phantosmia was created by disorganization of normal CNS pathways secondary to induction of CNS plasticity related to peripheral hypersensitivity and was corrected by rTMS through reorganization of the disorganized CNS. 

#### 4.1.1. Valsalva Effects

Although mechanism(s) responsible for physiological termination of phantosmia in CUP may be complex, maneuvers, which inhibited phantosmia may offer clues as to CNS mechanism(s) of these effects and to the syndrome itself. A consistent method for phantosmia inhibition in CUP followed performance of a Valsalva maneuver. The normal Valsalva response involves an initial transient rise in systemic blood pressure, then a gradual decrease in pulse pressure and stroke volume, due to decreased venous return to the heart, then a transient decrease in systemic blood pressure followed by a characteristic overshoot of systemic blood pressure over baseline [246,247]. Changes in CSF pressure accompany these effects [248] as do changes in cerebral blood flow. It is therapeutically useful in terminating paroxysmal supraventricular tachycardias [249], ventricular tachycardias [250] and angina related to coronary artery disease [251,252]. This latter effect has been attributed to an abrupt decrease in determinants of myocardial oxygen demand. Other effects attributed to this maneuver are doubling of portal vein pressure in patients with cirrhosis [253] and an increase in factor VIII concentration attributed to an increase in circulating epinephrine [254]. Perhaps some aspect of changes in CSF pressure, cerebral blood flow or local catecholamine concentration may play roles in inhibiting phantosmia in patients with CUP. Studies using brain fMRI in patients with CUP demonstrated that Valsalva performance, which inhibited phantosmia in patients with their phantosmia initiated, increased brain fMRI activation over the already robust activation measured with the initiated phantosmia [104]. This result is consistent with increased regional cerebral blood flow creating a transient state of oxyhemoglobin abundance such that use of pulse sequences that are sensitive to changes in oxyhemoglobin/deoxyhemoglobin disparities (*i.e.*, BOLD imaging [255,256]) allows visualization of these transient changes.

However, Valsalva may also inhibit phantosmia through direct CNS effects. In an investigation of hyperventilation, orthostasis and Valsalva in healthy normal volunteers, Lempert *et al*. induced syncope, generalized and multifocal myoclonic jerks, visual and auditory hallucinations and EEG changes of high-amplitude slowing and flattening [257], with hyperventilation, then Valsalva [258,259]. During Valsalva, brain fMRI activation in normal volunteers was observed in putamen, anterior hypothalamus, interpeduncular nuclei, basal and dorsal pons, deep cerebellar nuclei, ventral medullary surface and dorsal column nuclei [260] but not in regions of frontal cortex, a region in which we previously observed significant activation in brain fMRI studies of patients with both CUP [105] and BPAS [35,104]. These studies suggest that while CNS changes in CUP may have a different locus from those in normal volunteers, Valsalva may alter the balance of CNS excitation/inhibition. While the multiple processes underlying this complex interaction were not addressed in any previous study it was hypothesized that frontal cortex would be a brain region that would be most sensitive to hypocapnia and changes in arterial pCO_2_ [261].

If this latter hypothesis were correct then sensitivity of a focal brain region in frontal cortex to hypocapnia and changes in pCO_2_ suggest another possible mechanism by which Valsalva could inhibit phantosmia and by which any physiological activity that would mimic hyperventilation could initiate it. It is well known that GABA activation or inhibition is very sensitive to focal changes in brain pH [262]. Thus, phantosmia inhibition by Valsalva may relate to partial breath holding associated with this maneuver with a subsequent transient focal brain CO_2_ accumulation with focal acidification of enough magnitude to initiate GABAergic activation. On the other hand, forced ventilation, strenuous physical activity, coughing or sneezing may initiate transient focal brain CO_2_ excretion of sufficient magnitude to initiate brain alkalization with subsequent diminution in GABAergic inhibitory function. Since CNS kindling has been considered to be a type of use or activity dependent CNS plasticity with inherent alterations at both single cells as well as CNS neuronal networks [263], this concept is consistent with our hypotheses related to pathogenesis of phantosmia induction and inhibition in CUP. Thus, we can speculate that there may be a specific focal brain area especially sensitive to CO_2_ and changes in GABA [264] in these patients such that a type of chemical kindling [265,266,267] trigger effect might take place. Perhaps olfactory bulbs, anterior frontal cortex and rhinencephalic networks, anatomical regions known to be most sensitive to early and late stage kindling effects [268,269,270,271,272,273], might be logical regions for these effects to occur. Kindled seizures have been shown to be both hormonally [274,275,276] as well as possibly CO_2_ [277,278,279,280,281] dependent and it is well known that there is a loss or reduction in GABAergic inhibition in epileptic tissue [282] associated with disinhibition leading to hyperexcitability and generation of epileptiform bursting. Chemical kindling describes a progressive and persistent change in focal brain sensitivity, which may be initially sub-threshold for evoking an electrical or epileptic discharge, but which we speculate may eventually evoke full-blown phantosmia. A decrease in CO_2_ in this region could initiate a trigger related focal alkalization, decrease in GABAergic inhibitory activity and subsequent onset of phantosmia; a focal increase in CO_2_ could initiate a focal acidification, activation of GABAergic inhibitory activity and subsequent inhibition of phantosmia. Thus, any maneuver that would increase CNS pCO_2_ would be associated with phantosmia inhibition and any maneuver associated with decreased CNS pCO_2_ would be associated with phantosmia activation. Indeed, raised tissue concentrations of CO_2_ have been hypothesized to be protective in CNS whereas lowered concentrations have been considered injurious [282]. In this sense, the disease process in these patients could take the form of a focal brain abnormality in chemosensation such that a focal brain area exceedingly sensitive to changes in pCO_2_ and pH could trigger either GABAergic inhibition or activation with subsequent onset or offset, respectively, of a chemically induced phantosmia and/or phantageusia. This hypothesis is supported by results of CO_2_ inhalation studies in which seven of the 10 patients treated with CO_2_ inhalation reported phantosmia inhibition with a subsequent inability to initiate phantosmia by those maneuvers, which prior to CO_2_ inhalation uniformly initiated phantosmia. We suggest that this focal CNS area lies within the region of anterior frontal and/or temporal cortex [35,104], the CNS region found activated during patient activation of phantosmia.

#### 4.1.2. CO_2_ Effects

Increased CO_2_ concentrations (hypercarbia) initiate triphasic CNS effects [283,284]. Relatively low CO_2_ levels (5%–10%) induced depression in CNS excitability as measured by increased electroshock thresholds, protection against convulsions or seizures induced by electroshock or chemical agents [284] and fast, low voltage EEG waves [284]. As CO_2_ concentrations increased to 30%–35% of inspired air, increased CNS excitability was observed with experimental animals and humans exhibiting minimal type-missing seizures [285] and slow, high voltage EEG activation [284,286,287]. If CO_2_ concentrations were increased still further, to greater than 40% of inspired air, narcosis and anesthesia occurred [288,289,290], whereas if high concentrations of CO_2_ were rapidly withdrawn, associated with a rapid decrease in blood CO_2_, clonic type seizures occurred [291,292]. CO_2_ is rapidly transported into CNS and cerebral spinal fluid (CSF) with a time constant of 2.7 min [293] with an active transport of H^+^ out of cells and consumption of organic acids [294]. Although acute CO_2_ effects in CNS are difficult to measure, transmembrane fluxes of HCO3^-^ and H^+^ active transport were measureable in 45 min and return of cell pH to normal can take as long as 48 h [295]. Permissive hypercapnia in which CO_2_ concentrations were increased was associated with protection from organ injury in patients with a variety of pulmonary disorders [282]. 

Exposure to 35% CO_2_ induced panic attacks [296] whereas CO_2_ exposure inhibited phantosmia in CUP. Indeed, CO_2_ challenge was considered the most informative challenge to induce panic disorder [297] because the prominent respiratory symptom in panic could be linked to a measureable abnormality (increased ventilatory responsiveness with a known physiological basis [298]). While glutamate release has not been generally observed during acute seizure induction in experimental animals, in chronic epilepsy models of several types, including those with spontaneous recurrent seizures after amygdala kindling [299], there has been a consistent marked increase in glutamate release during seizures [295]. While other maneuvers, which initiated phantosmia in CUP, may be less obviously related to hyperventilation they appear to activate similar mechanisms, which involve changes in blood and CNS pH with subsequent decreased CNS GABA and onset of an imbalance between excitation and inhibition such that excitation is emphasized, and phantosmia appears.

Breath holding, the opposite effect in terms of control of hyperventilation, resulted in increased end-tidal CO_2_, inhibited seizure frequency and severity in patients with severe chronic hyperventilation and seizures which were refractory to therapeutic levels of anticonvulsants [300]. After breath holding, signal intensity observed in human CNS during fMRI increased 3%–10% [301,302,303,304]. It has also been suggested that evolutionary changes were developed to set a particular level of focal synaptic ratio of CNS excitation; inhibition based upon metabolic protection of hypoxia and hypoglycemia and dependent upon synthesis of GABA from glutamate (dependent upon input from acetyl CoA and citrate from the Krebs cycle (through GAD)) and degradation of GABA (by GABA transaminase to succinic semialdahyde to succinate and back into the Krebs cycle [305]). This so called ‘GABA shunt’ is highly sensitive to changes in pCO_2_ and pH with increased pCO_2_ and decreased pH resulting in a shift in this reaction toward glutamate with increased brain excitability and with decreased pCO_2_ and increased pH resulting in a shift in the reactivity toward increased GABA with decreased brain excitability.

#### 4.1.3. Hyperventilation Effects

It has long been recognized that hyperventilation (hypocarbia) giving rise to hypocapnia precipitated seizures in epileptic patients [295,299,306,307,308], induced abnormal EEGs in both epileptic and some normal subjects (increased high voltage, slow waves [308,309,310]), independent of inspired O_2_ concentration [311] due to cerebral hypoxia [312,313,314] and/or cerebral vasoconstriction [312], induced decreased blood CO_2_ [283,310,315], cerebral arterial blood CO_2_ tension ((PaCO_2_) [316]) and brain pCO_2_ [316]. Indeed, hyperventilation is a well-known standard challenge in electroencephalography to induce CNS hyperactivity. Thus, there are intimate correlations among respiration, brain waves and seizure activity dependent upon changes in cerebral vascular tone, blood flow and cerebral arterial pCO_2_. Two deep breaths will reduce arterial CO_2_ significantly [285] and further hyperventilation will result in respiratory alkalosis [285]. Doppler ultrasound indicated mean flow velocity in middle and posterior cerebral arteries decreased by up to 50% after four min of hyperventilation [317]; it also led to marked surface negative DC shift, perhaps due to depolarization of apical dendritic trees of cortical pyramidal cells [318,319] which increased excitability of cortical neuronal networks and may explain the resultant potential epileptogenicity [285]. Hyperventilation eliminates CNS lactate rapidly [285,320] which may be reflected in increased GAD concentration and decreased GABA. Hyperventilation strongly reduced global CNS magnetic resonance signal intensity [321,322] reflecting increased oxygen extraction due to cerebral vasoconstriction and decreased cerebral blood flow [323]. This maneuver is also consistent with induction of several neurotransmitters including glutamate, *N*-methyl-d-aspartate (NMDA), α-amino-3-hydroxy-5-methyl-isoxazole-4-propriovic acid (AMPA) which have been implicated in initiation of seizure or seizure-like activity [315]. The hypocapnia due to hyperventilation not only reduced cerebral blood flow and volume but also induced decreased signal response during functional imaging of the visual cortex with BOLD-contrast MRI [323]. By reducing cerebral blood flow the associated increased alkalosis shifts the oxyhemoglobin dissociation curve to the left further limiting oxygen delivery to the CNS [285] and further impairing oxidative metabolism [322,323].

Cerebral hypoxia, linked to hyperventilation, has been considered etiologic in epilepsy [324,325] and seizure induction [285], associated with shift in the oxygen dissociation curve (Bohr effect [326,327,328]). These results suggest that acid-base balance in epileptics is crucial to control of seizure thresholds and that successful strategies for seizure control need to consider physiological consequences of respiratory alkalosis [329] as manifested by activities such as hyperventilation. Indeed, patients with so-called “idiopathic seizures” have been considered different from normal subjects due to intolerance to deviation from critical limits of cerebral PaCO_2_ [329]. This concept has led some investigators to believe that self-control of end-tidal CO_2_ through respiratory training could control intractable “idiopathic seizures” [329].

However, there is confusion about relationships among hyperventilation, panic attacks and epilepsy [285]. Indeed, confusion can also occur with respect to what has been called chronic hyperventilation syndrome [330], panic disorder [331,332], hyperventilation induced seizures [333], phobic anxiety depersonalization syndrome [293] and Rett’s syndrome [334]. Hyperventilation induced seizures, albeit rarely tonic-clonic or partial [333], has been the subject of many case reports [300,329,335]. Many of these involved adults or children with mental retardation with what were commonly characterized as absence or atypical absence attacks. Physical exercise [284,336] but not hyperventilation [285] has been shown to induce seizure activity in some patients. Mechanisms underlying these events are unclear but none had hallucinatory activity related to seizure induction [285].

#### 4.1.4. O_2_ Effects

For O_2_, seizures were induced at both abnormally high (hyperoxia) or abnormally low (hypoxia) levels. Hypoxia induced convulsions were first reported in birds, mice and cat, relatively sensitive species, by Boyle in 1660 [337], as have many more recent investigators [338,339,340] including studies in humans [341]. Recovery from an hypoxic episode in animals was associated with nervous system excitation [338,339,342]. While hypoxia can induce seizures under less dramatic conditions [343] hypoxia induced striking increases in brain GABA [344,345]. Bert first noted animals breathing oxygen at high pressure to initiate seizure activity in 1879 [346] and subsequently studied by many others [347,348]. These effects are dependent upon many factors including exposure to O_2_ under increased pressure [349], decreased CO_2_ [350], species susceptibility [343], male gender, since females are more sensitive than males [351] and nutritional status, since fasting induces decreased susceptibility [343]. Under these conditions of increased O_2_ exposure, GAD is very readily oxidized and inhibited *in vivo* resulting in decreased CNS GABA [343]. The correlation between susceptibility to seizures and decreased CNS GABA is well known [237,341]; brain GABA levels increase in epileptics treated with GABAergic drugs [352,353].

#### 4.1.5. Sleep Effects

Sleep usually inhibited phantosmia in CUP if “deep enough sleep” were obtained whereas no effect in BPAS was reported. This suggests the hypothesis that slow wave, REM sleep or both inhibited phantosmia in CUP. While clinical history does not allow a definitive answer the fact that “deep sleep” was required for this inhibition to occur and that its effectiveness was greatest if a nap were taken in the afternoon not in the morning suggests that slow wave not REM sleep was the mechanism through which this inhibition occurred, consistent with the well-known effect of latency to slow wave sleep longer in the morning than in the afternoon or early evening hours [354]. However, data in animals and in humans relate to roles that each of these sleep stages may play in this inhibitory process. REM sleep was initiated by administration of cholinergic agonists into pons [355,356]. Sleep related seizures in partial epilepsy occurred more than 20 times as frequently in non-REM (NREM) as opposed to REM sleep [357] and interictal epileptiform discharges in patients with epilepsy are more common in NREM than in REM sleep [358]. These results suggest that REM sleep may be considered neuroprotective and supports a role it may play in inhibiting phantosmia in these patients. On the other hand, sleep spindle activity has been associated with GABA release from thalamic reticular neurons and excitatory actions of thalamocortical (or relay) neurons which project to cerebral cortex and then project back to relay cells and inhibit them initiating a recurrent inhibitory circuit [359,360]. Thalamic perfusion of GABA in cats induced increased slow wave and paradoxical sleep as well as long lasting inhibition of somatosensory event-related potentials [361]. These results suggest slow wave sleep, through endogenous action of GABA release, may inhibit phantosmia in CUP. Clinically, as patients with CUP either aged or experienced phantosmia for a longer time period, sleep became less effective in inhibiting phantosmia consistent with the well-known phenomenon that time spent in slow wave sleep decreases with age [362]. 

#### 4.1.6. Hormonal Effects

Hormonal factors may influence CUP more than BPAS since significantly more women than men (3:1, women:men) had CUP than had BPAS (~1:1). Phantosmia was initiated in CUP in two women during pregnancy or immediately postpartum suggesting a role for estrogen, progesterone and/or prolactin. In at least 72% of women suffering from catamenial epilepsy, menses exacerbated seizures related, in part, to withdrawal of allopregnanolone, a progesterone metabolite, which acts as an endogenous anticonvulsant neurocorticosteroid that modulates GABA_A_ receptors [363,364,365,366,367]. The putative effect of estrogen or lack thereof on phantosmia generation may also relate to the well-known interrelationship between this hormone and brain GABA and/or GABA_A_ receptors. Estrogen specific effects on neuronal survival, differentiation and neurite extension suggested that this hormone modulates neurotransmitter and neuropeptide expression as well as tissue specific alterations in neural cell structure [368]. Estrogen has also been shown to bind specifically to preoptic GABAergic neurons [369] and to inhibit synthesis of GAD thereby decreasing local GABA concentration [370], Estrogen also acts to release neuronal epinephrine and nonepinepherine [371,372] although GABA may suppress CNS norepinephrine release [371]. Pregnenolone modulates GABA and GABA_A_ receptor function thereby influencing GABA activity [373,374,375,376]. Conversely, there are powerful and complex feedback mechanisms whereby GABA acts as an inhibitory neurotransmitter restricting LHRH release [377] thereby inhibiting pituitary LH secretion and inhibiting gonadal estrogen [378]. Ovariectomized rats have been shown to exemplify this feedback mechanism [379] as have pubertal and prepubertal primates [380]. Estrogen is also known to play a role in neuronal plasticity [381,382,383] in part through neuronal sprouting [384,385]. 

Women have a higher hemispheric cerebral blood flow than men [386,387] reflected in a 3%–5% higher blood flow velocity in the middle cerebral artery [388]. Since blood flow velocity is dependent upon pCO_2_, gender differences in pCO_2_ indicate differences in blood flow velocity as measured by transcranial Doppler sonography [389]. Studies by Kastrup *et al*. demonstrate not only that women have a significantly higher cerebrovascular CO_2_ reactivity than men [389] but also that this difference disappears as women enter menopause and reappears upon treatment with hormone replacement therapy [390], presumably estrogenic compounds, and presumably based upon estrogenic effects, enhancing cerebrovascular reactivity.

These results suggest young women, who comprise the major subject group of CUP, may be particularly susceptible to these effects. However, studies in rat indicate GABAergic neuronal activity is greater in males than in females [391,392] although studies in sheep demonstrate that estrogen reactive cells colocalize with GABA [393]. Gender differences in CNS turnover of GABA have also been clearly demonstrated [394,395,396,397]. It is well known that estrogen administration to ovariectomized rats has proconvulsant effects [398], that increased seizure frequency occurs in follicular phase of menstrual cycle when estrogen concentration has peaked [399] and that seizures were exacerbated in women given estrogen premenstrually to treat catamenial epilepsy [400]. GABAergic neurons are present in olfactory bulbs and in hypothalamus in both embryonic [401] and adult [402] animals and appear to play a role in hormonal regulation [403]. GABA has been localized to interneurons in the granular cell layer of olfactory bulb and appears to play a role in olfactory transmission [404,405]. GABA_A_ and GABA_B_ receptors have also been shown to be present in olfactory bulb [406]. These findings suggest that women might be expected to have lower baseline brain GABA levels, which might make them more susceptible to focal pCO_2_ changes than men. If they had relatively lower endogenous levels of brain GABA, coupled with focal brain pCO_2_ changes associated with hyperventilation, cough, forced expiration, *etc*., might initiate a type of chemical kindling which might make them more susceptible to activation of phantosmia than men. Indeed, all women in CUP were premenopausal whereas the majority of women with BPAS were postmenopausal.

#### 4.1.7. THC Effects

Recreational use of THC in one woman uniformly inhibiting phantosmia in CUP is consistent with the general hypothesis that GABA stimulation extinguishes phantosmia albeit there are several caveats to this effect. THC is mediated primarily through cannabinoid receptors which involve two types of G-protein-coupled cannabinoid receptors named CB_1_ and CB_2_ which have been identified and cloned [407]. Research data also suggest the presence of a third uncloned cannabinoid receptor [407]. Cannabinoids act at prejunctional CB_1_ receptors to inhibit sympathetic neurotransmission [408]. THC acts through presynaptic CB_1_ receptors which may reduce GABA_A_ but not GABA_B_ mediated inhibition of CA_1_ pyramidal neurons by inhibiting voltage dependent calcium channels located in inhibitory nerve terminals [409] including hippocampal neurons [410,411]. THC also reduced glutamate release through the action of G-protein mediated inhibition of these calcium channels which are responsible for neurotransmitter release from hippocampal neurons [410]. While THC has a single saturable, reversible binding site [412] to a specifically identified CNS receptor of the GTP class [413,414] several neurotransmitters, including GABA, play roles in its neuropharmacology. Several cannabinoid-related agents exert anticonvulsant activity [415] and THC has been reported to inhibit acetylcholine turnover in hippocampus by increasing activity of septal GABAergic activity [416]. In animal studies THC-like agents protect mice against 3-mercaptopropriovic acid-induced convulsions, an action specific for GABAergic agents [417], stereospecifically blocked isoniasid-induced levels of cGMP [417] and similar to other GABAergic drugs, activated dopaminergic and benzodiazapine systems [418]. THC-like agents exhibit several pharmacological effects similar to GABA [418] including increasing attenuation of dopamine turnover after haloperidol [419] and stereospecifically increased *in vivo* 3H-flunitrazepan binding to benzodiazepine receptors from mouse brain [417]. Consistent with this latter observation levo-, but not dextro-mantradol enhanced potency and efficacy of diazepam blockade of pentylenetetrazol induced seizures [417]. Chronic injections of THC increase GABA content of rat brain [420] and THC itself has been reported to have unique anticonvulsant properties [421]. Cannabinoids, albeit variably, inhibit neurotransmission [422] and have been reported to augment GABA effects [423]. One mM Δ^9^ THC was reported to induce a consistent and significant increase in amplitude of grease-gap recordings of rat hippocampal slices in response to GABA [196] and other investigators suggested that THC interacts with GABA to mediate synaptic transmission [424]. Behavioral studies show that Δ^9^ THC and drugs which stimulate or facilitate GABAergic transmission exhibit a marked synergy in abolishing righting reflexes in mice [425] and in producing catalepsy in mice and rats [425,426]. Mechanism(s) underlying these various effects are unclear [417]. However, on the other hand, THC has been reported to have both convulsant as well as anticonvulsant properties [421]. It can diminish effects of GABA [422,427], enhance focal epileptic potentials and seizure activity [428] and can inhibit uptake of GABA in hippocampus [196]. These results suggest that THC exhibits multiple effects on CNS GABA that may act through some common mechanism (s) such as influencing CNS Ca^2+^ uptake or reflex [417] alterations in activation of chloride channel proteins [429] or other mechanisms [430,431]. THC can also activate capsaicin-sensitive sensory nerves via the CB_1_ and CB_2_ receptor pathways suggesting other inhibitory pathways [432]. 

### 4.2. Pathophysiological, Peripheral and CNS Components of BPAS

In BPAS loss of sensory input (*i.e.*, hyposmia), as the initiating cause of phantosmia, is consistent with deafferentation hypersensitivity, similar to effects observed in phantom limb syndrome [433,434,435]. This effect may also be both rapid [213,216] or slow [218,219] associated with changes in GABAergic and GAD activity. These responses follow loss of a significant sensory input such that there are significant functional CNS changes including cortical enlargement, [434,435,436] rerouting and remapping of the usual cortical representation of the affected areas to specific brain areas [435,437,438] related to neuronal plasticity. These changes have been visualized by others studying deafferentation hypersensitivity related to various clinical syndromes using fMRI [439,440], magnetoencephalography [441] and PET [434] and in our own studies in BPAS using fMRI [35]. 

### 4.3. Common Clinical CNS Components in Both CUP and BPAS

Both CUP and BPAS exhibited EEG abnormalities although CUP exhibited significantly more frequent and specific EEG changes than did BPAS, despite the fact that 45% of patients with BPAS had symptoms secondary to a head injury. In CUP, EEG changes, if considered separately, could be and were considered clinically consistent with a temporal lobe seizure disorder. Indeed, 60% of CUP patients were initially considered to exhibit some form of epilepsy and were treated with anticonvulsants although none experienced phantosmia diminution with this therapy [104]. In our initial description of this disorder [155] we also considered CUP a partial seizure disorder with simple psychosensory symptomatology since patients exhibited what were considered auras commonly associated with temporal lobe EEG abnormalities. However, the lack of clinical motor activity cast doubt about this diagnosis even at this initial stage of our studies. Our further investigations allowed a more definitive description of this disorder and its possible mechanism [104].

In BPAS, while a peripheral component of phantosmia may be present, it may be less immediately apparent than in CUP. While binasal cocaine installation inhibited phantosmia in BPAS, as observed by others [442,443], this inhibition was only temporary. In CUP there was usually a post anesthesia inhibitory period along with the production of hyposmia. These results may relate to activation of GABAergic pathways after cocaine installation [222,224] but the issue of initial deafferentation hypersensitivity and decreased GABAergic activity prior to its use must be considered. rTMS was effective in inhibiting phantosmia in both CUP or BPAS consistent with inhibition of deafferentation hypersensitivity presumably by inducing increased CNS GABA [165,166].

### 4.4. Other Neurotransmitter Effects on CNS in CUP and BPAS

Several other neurotransmitters may also be involved in complex processes that initiate phantosmia in CUP (and also in BPAS) including relationships between both excitatory and inhibitory neurotransmitter systems related to various aspects of their synthesis, utilization or interaction with specific receptors. Hallucinations, usually visual or auditory, have been reported in Parkinson’s disease [444] due in part to CNS deficits in cholinergic [445,446,447] or dopaminergic [448,449] function. Drugs have induced hallucinations due to increased glutaminergic activity [450] or decreased GABAergic function [199]. Phenobarbital, which increases CNS GABAergic activity [451,452,453], inhibited phantosmia in one patient with CUP. Indeed, both thioridazine and haloperidol, neuroleptics that enhance GABAergic CNS activity [198,199,454,455] inhibited phantosmia in both CUP [104] and BPAS [35].

### 4.5. Behavioral and Sensory Effects in CUP and BPAS

Both seizure inhibition and activation by behavioral and sensory activities have been shown in patients with known seizure activity [456]. For seizure inhibition these activities have been classified as primary (direct seizure inhibition by an act of will) or secondary [by a behavior or mind action which interferes with seizure generation but was not intended to do so [456]]. Whereas 10% of patients with complex partial seizures were able to reduce their seizure frequency by behavioral means [457] patients with simple partial seizures were reportedly not able to do so [456]. Olfactory stimuli inhibited seizure activity [458] as has operant conditioning [459], behavior modification [460,461,462], relaxation programs [463] and other similar activities [456]. Historically, it is well known that focal epileptic seizures can be aborted by powerful sensory stimuli such as application of a tight ligature to a finger, vigorous massage of hand and foot muscles, forcibly resisting the first tonic seizure spasm, plunging a whole extremity into ice water or extremity pin pricking [464], related to activation of a widespread suppressor system of nociceptive impulses [465,466]. Gowers in 1885 wrote that seizures could be arrested by taste stimuli such as chewing ginger or swallowing table salt or olfactory stimuli such as smelling ammonia or amyl nitrite [17]. Efron’s studies suggest that olfactory stimuli that inhibited uncinate seizures were independent of psychological suggestion and related to activation of a localized inhibitory system [458]. 

On the other hand clinical experience suggests that there can be a close interrelationship between what may be considered “atypical behavior” and what some clinicians might consider seizure activity [467,468,469,470] such that sensory and other activities might activate seizure activity. Many adolescents reported auditory hallucinations without any psychotic ideation [471]. So-called psychogenic pseudoseizures have been reported in non-epileptic patients precipitated by suggestion or environmental stress but none had EEG changes either before or after these “attacks” [472]. As many as 20% of epileptics have been reported to have nonepileptic or “pseudoepileptic” episodes simulating status epilepticus [473]. Reflex seizures [474,475,476], sensory precipitation epilepsy [476] and stimulus-sensitive epilepsy precipitated by light [477,478], vestibular stimuli [479,480], music [481] tastes [482], propriociption [483], somatosensory stimulation [484], eating [482,485,486], startle and/or arousal [459,487], integration of higher cerebral functioning or thinking, reading and decision making [488,489] have been reported. Seizures of these various types have been related to an imbalance between GABAergic and dopaminergic neurotransmission [476] and initiated in animals following strychnization of sensory cortex, including olfactory cortex [490]. Seizures of this type have been reported evoked or self induced in as many as 30% of patients with so-called photoparoxysmal responses [491], have been estimated to occur in 5% of people with epilepsy [461], have been considered genetically determined [492] and occurred more commonly in young women than in men [492]. Psychogenic [493] or pseudoseizures [[494,495,496] (hysterical or nonepileptic seizures)] have been provoked by some psychic event [469,493], can be deliberately precipitated, have been estimated to occur in five to 40% of outpatients with epilepsy evaluated at epilepsy centers [469,497], again most commonly observed in young women [493] and have been related, particularly in animals, to activation in a single, focal brain area [490]. Some investigators considered epilepsy rare in patients with psychogenic seizures [498] whereas others found nonepileptic seizures and epilepsy, per se, to co-occur in 12%–42% of patients [499] making these discriminations difficult. 

Although seizure activity of the type described above is diffuse and difficult to specify none of these patients reported hallucinatory activity of the type observed in either CUP or BPAS. However, the symptomatology of these latter two syndromes may itself cause clinical confusion due to their protean character. At initial presentation it might be understandable that patients with CUP were considered to manifest a seizure disorder. However, while various types of seizures and mechanisms for their initiation and inhibition may be relatively common and difficult to classify, patients with epileptic seizures can be readily distinguishable from those with either CUP or BPAS by careful history. Thus, in both CUP and BPAS, there is a much longer duration of each sensory event which has a pattern of persistent intermittancy, a sudden onset of symptoms as opposed to the gradual onset of ictus, a lack of any overt autonomous activity as opposed to wild movements, intermittent jerking and myoclonic activity in the other disorders, a fixed, stereotypic phantosmia as opposed to non-fixed stimulus evoking and fluctuating clinical features in the other disorders, no type of post-ictal symptomatology which occur in each of these other disorders and in CUP, specific physiological not behavioral stimuli which initiate and/or inhibit phantosmia as opposed to the lack of these specific stimuli in either initiating or inhibiting activity in either BPAS or the other disorders.

Many of the patients with both CUP and BPAS expressed great personal distress associated with the presence of these hallucinations. Their presence, their intensity, their recurrent chronicity, the lack of understanding of the severity of their presence and the lack of effective treatment exacerbated their personal distress. Some patients likened their hallucinations to a recurrence or persistence of a chronic, recurrent nasal or oral pain without effective relief with which they had a great deal of difficulty in tolerating similar to the experience noted by others in relationship to the presence of chronic somatic pain [500]. 

### 4.6. Epistemology of Sensory Hallucinations

Nomenclature related to sensory hallucinations has been historically diffuse and unclear (v.s.). Some investigators used the term ‘pseudo-hallucination’ to differentiate onset of unprovoked odors from “true hallucinations” [501,502]. This terminology is vague and not only does it not accurately describe the sensory phenomena experienced in either CUP or BPAS but also does not account for demonstration of sensory specific brain fMRI activation associated and measured in each syndrome [35,104]. A variety of other terms have been used to describe unusual, odd or peculiar “behavior” that may be and has been considered consistent with some type of seizure disorder. These terms include some associated with sensory behavior that could be considered an aura involving any number of sensory systems, including olfaction and taste [8,93,137,503,504]. We speculate that these behaviors may have a common origin. CUP and BPAS are manifestations of clinical and biochemical phenomena which at other times might have been considered odd or unusual behaviors but since they have specific neurological correlates they can be categorized specifically, whereas some of these other behaviors, without specific neurological correlates, were simply considered bizarre behaviors.

Attempts to systematize sensory hallucinations may assist to clarify mechanisms underlying phantosmia in CUP and BPAS. We have used the word phantosmia to refer to smell sensations induced in the absence of any known stimulus and the word phantageusia to refer to similar taste sensations. We classified phantosmia and phantageusia as primary or secondary [35], similar to terminology used in dystonia [505] or Parkinsonism [506]. Primary phantosmia or phantageusia was defined as an odor or taste occurring in absence of any apparent stimulus in the absence of any apparent clinical or disease process; secondary phantosmia or phantageusia was defined as an odor or taste occurring in the absence of any apparent stimulus associated with a clinical disease process [35]. Previously, the phantom, which we have classified as either primary or secondary phantosmia or phantageusia, was classified as an aura which may be considered a simple partial seizure [507,508]. We consider these symptoms to be pathophysiological processes involving a trigger mechanism (be it chemical kindling or some other similar mechanism) and a process in which CNS is altered due to what we consider an alteration in the complex balance between glutaminergic or similar neurotransmitter related CNS excitation and GABAergic or similar type inhibition in a type of maladaptive plasticity [35]; for CUP we hypothesize this effect initiated as a type of receptor hypersensitivity with subsequent CNS plasticity [as in dystonia [181,184]] and for BPAS we hypothesize this effect initiated as a type of deafferentiation hypersensitivity with subsequent CNS dysplasticity (as in phantom limb pain [433,438,441]). It is possible to consider the “secondary” phantosmia and phantageusia described by Jackson [6,7,8] and others [92] as another manifestation of chemical/electrical kindling set in motion by a tumor discharge or epileptic focus which can result in impaired consciousness, clonic movement or automotisms or which can itself, without a specific localizing epileptic focus, represent a manifestation of the seizure itself.

The observations we describe in both CUP and BPAS in which myoclonic activity does not occur relate to new, heretofore unrecognized applications of well-established and well known physiological principles dealing principally with changes in CNS plasticity and in CNS GABA.

## 5. Conclusions

We describe two previously underrecognized syndromes, one with cyclic unirhinal phantosmia not directly associated with hyposmia labeled CUP and one with non-cyclic birhinal phantosmia directly associated with hyposmia labeled BPAS, but both without prior or subsequent myoclonic activity. Patients with CUP reflect an uncommon clinical syndrome whereas we suggest that patients with BPAS are relatively common although they are not recognized as such by the neurological or otorhinological communities. Studies of the mechanisms related to these disorders suggest that decreased CNS GABA activity in specific brain regions is associated with phantosmia in CUP whereas changes in CNS plasticity consistent with changes observed in phantom limb sensations are associated with phantosmia in BPAS. GABA agonists, increased either by pharmacological or neurophysiological means, have been useful in inhibiting phantosmia in each patient group and suggest the relevance of levels of CNS GABA in both initiation and inhibition of these two syndromes.

## References

[1] Aretaeus, Adams F. (1978). The Extant Works of Aretaeus: The Cappadocian.

[2] Kühn C.G. (1964). Claudii Galeni Opera Omnia.

[3] Manford M., Shorvon S.D. (1992). Prolonged sensory or visceral symptoms: An under-diagnosed form of non-convulsive focal (simple partial) status epilepticus. J. Neurol. Neurosurg. Psychiatry.

[4] Gove P.B. (1967). Webster’s Seventh New Collegiate Dictionary.

[5] Esquirol E. Des Maladies Mentales Considérées sous les Rapports Médical, Hygiénique et Médico-Légal, (1838).

[6] Jackson J.H. (1866). Subjective sensations of smell with epileptiform attacks. R. Lond. Ophthalmic Hosp. Rep..

[7] Jackson J.H. (1866). Clinical remarks on the occasional occurrence of subjective sensations of smell in patients who are liable to epileptiform seizures or who have symptoms of mental derangement and in others. Lancet.

[8] Jackson J.H. (1871). Subjective sensation of smell with epileptiform seizures. Lancet.

[9] Lennox W.G., Cobb S. (1933). Aura in epilepsy: A statistical review of 1,359 cases. Arch. Neurol. Psychiatry.

[10] Penfield W., Kristiansen K. (1951). Epileptic Seizure Patterns.

[11] Gastaut H., Broughton R.J. (1972). Epileptic Seizures: Clinical and Electrographic Features, Diagnosis and Treatment.

[12] Daly D.D. (1975). Ictal clinical manifestations of complex partial seizures. Adv. Neurol..

[13] Weiser H.G. (1983). Electroclinical Features of Psychomotor Seizure.

[14] Currie S., Heathfield W.G., Henson R.A., Scott D.F. (1971). Clinical course and prognosis of temporal lobe epilepsy. A survey of 666 patients. Brain.

[15] Commission on Classification and Terminology of the International League against Epilepsy (1981). Proposal for revised clinical and electroencephalographic classification of epileptic seizures. Epilepsia.

[16] Gupta A.K., Jeavons P.M., Hughes R.C., Covanis A. (1983). Aura in temporal lobe epilepsy: Clinical and electroencephalographic correlation. J. Neurol. Neurosurg. Psychiatry.

[17] Gowers W.R. (1885). Epilepsy and Other Chronic Convulsive Diseases: Their Causes, Symptoms and Treatment.

[18] Jasper H.H., Rasmussen T. (1958). Studies of clinical and electrical responses to deep temporal stimulation in man with some considerations of functional anatomy. Res. Publ. Assoc. Res. Nerv. Ment. Dis..

[19] Falconer M.A., Cavanagh J.B. (1959). Clinico-pathological considerations of temporal lobe epilepsy due to small focal lesions. Brain.

[20] Van Buren J.M. (1963). The abdominal aura: A study of abdominal sensations occurring in epilepsy and produced by depth stimulation. Electroencephalogr. Clin. Neurophysiol..

[21] Penfield W., Jasper H.H. (1954). Epilepsy and the Functional Anatomy of the Human Brain.

[22] Mullan S., Penfield W. (1959). Illusions of comparative interpretation and emotion; production by epileptic discharge and by electrical stimulation in the temporal cortex. Arch. Neurol. Psychiatry.

[23] Penfield W., Perot P. (1963). The brain’s record of auditory and visual experience. A final summary and discussion. Brain.

[24] Gloor P., Olivier A., Quesney L., Andermann F., Horowitz S. (1982). The role of the limbic system in experiential phenomena of temporal lobe epilepsy. Ann. Neurol..

[25] Sperling M.R., Lieb J.P., Engel J., Crandall P.H. (1989). Prognostic significance of independent auras in temporal lobe seizures. Epilepsia.

[26] Palmini A., Gloor P. (1992). The localizing value of auras in partial seizures: A prospective and retrospective study. Neurology.

[27] Taylor D.C., Lochery M. (1987). Temporal lobe epilepsy: Origin and significance of simple and complex auras. J. Neurol. Neurosurg. Psychiatry.

[28] Williamson P.D., Wieser H.G., Delgado-Escueta A.V., Engel J. (1987). Clinical Characteristics of Partial Seizures. Surgical Treatment of the Epilepsies.

[29] Ajmone-Marsan C., Engel J. (1987). Commentary: Clinical Characteristics of Partial Seizures. Surgical Treatment of the Epilepsies.

[30] Janati A., Nowack W.J., Dorsey S., Chesser M.Z. (1990). Correlative study of interictal electroencephalogram and aura in complex partial seizures. Epilepsia.

[31] Sperling M.R., O’Connor M.J. (1990). Auras and subclinical seizures: Characteristics and prognostic significance. Ann. Neurol..

[32] King D.W., Marsan C.A. (1977). Clinical features and ictal patterns in epileptic patients with EEG temporal lobe foci. Ann. Neurol..

[33] Hausser-Hauw C., Bancaud J. (1987). Gustatory hallucinations in epileptic seizures. Electrophysiological, clinical and anatomical correlates. Brain.

[34] Carter J.L. (1992). Visual, somatosensory, olfactory and gustatory hallucinations. Psychiatr. Clin. N. Am..

[35] Henkin R.I., Levy L.M., Lin C.S. (2000). Taste and smell phantoms revealed by brain functional MRI (fMRI). J. Comput. Assist. Tomogr..

[36] Levy L.M., Henkin R.I. (2004). Brain gamma-aminobutyric acid levels are decreased in patients with phantageusia and phantosmia demonstrated by magnetic resonance spectroscopy. J. Comput. Assist. Tomogr..

[37] Jackson J.H, Beevor C.E. (1890). Case of tumor of the right temporo-sphenoidal lobe, bearing on the localization of the sense of smell and on the interpretation of a particular variety of epilepsy. Brain.

[38] Jackson J.H., Stewart P. (1899). Epileptic attacks with a warning of a crude sensation of smell and the intellectual aura (Dreamy state) in a patient who had symptoms pointing to gross organic disease of the right temporo-sphenoidal lobe. Brain.

[39] Jackson J.H., Taylor J. (1931–1932). Lectures on the Diagnosis of Epilepsy. On Epilepsy and Epileptiform Convulsions, Selected Writings of John Hughlings Jackson.

[40] Gloor P., Purpura D.P., Penry J.K., Walter R.D. (1975). Contributions of Electroencephalography and Electrocorticography to the Neurosurgical Treatment of the Epilepsies. Neurosurgical Management of the Epilepsies.

[41] Gloor P., van Duijn H., Donker D.N., van Huffelen A.C. (1977). The EEG and Differential Diagnosis of Epilepsy. Current Concepts in Clinical Neurophysiology.

[42] Devinsky O., Kelley K., Porter R.J., Theodore W.H. (1988). Clinical and electroencephalographic features of simple partial seizures. Neurology.

[43] Lieb J.P., Walsh G.O., Babb T.L., Walter R.D., Crandall P.H. (1976). A comparison of EEG seizure patterns recorded with surface and depth electrodes in patients with temporal lobe epilepsy. Epilepsia.

[44] Klass D.W., Espinosa R.E., Fischer-Williams M. (1973). Analysis of concurrent electroencephalographic and clinical events occurring sequentially during partial seizures. Electroencephalogr. Clin. Neurophysiol..

[45] Theodore W.H., Porter R.J., Penry J.K. (1983). Complex partial seizures: Clinical characteristics and differential diagnosis. Neurology.

[46] Broglin D., Delgado-Escueta A.V., Walsh G.O., Bancaud J., Chauvel P. (1992). Clinical approach to the patient with seizures and epilepsies of frontal origin. Adv. Neurol..

[47] Fried I., Spencer D.D., Spenser S.S. (1995). The anatomy of epileptic auras: Focal pathology and surgical outcome. J. Neurosurg..

[48] Rose F.C., Adelman G., Smith B.H. (1999). Migraine. Encyclopedia of Neuroscience.

[49] Wolberg F.L., Ziegler D.K. (1982). Olfactory hallucination in migraine. Arch. Neurol..

[50] Crosley C.J., Dhamoon S. (1983). Migrainous olfactory aura in a family. Arch. Neurol..

[51] Diamond S., Freitag F.G., Prager J., Gandi S. (1985). Olfactory aura in migraine. N. Engl. J. Med..

[52] Daniel C., Donnet A. (2011). Migrainous complex hallucinations in a 17-year-old adolescent. Headache.

[53] Coleman E.R., Grosberg B.M., Robbins M.S. (2011). Olfactory hallucinations in primary headache disorders: Case series and literature review. Cephalalgia.

[54] Demarquay G., Créac’h C., Peyron R. (2012). Olfactory hallucinations in primary headache disorders: Case series and literature review. A comment. Cephalalgia.

[55] Benemei S., Eleonora R., Geppetti P. (2012). Trigeminal nerve and phantosmia in primary headaches. Cephalalgia.

[56] Gardner K. (1999). The genetic basis of migraine: How much do we know?. Can. J. Neurol. Sci..

[57] Ducros A. (2000). Genetics of migraine. Pathol. Biol. (Paris).

[58] Khalil N.M., Legg N.J., Anderson D.J. (2000). Long term decline of P100 amplitude in migraine with aura. J. Neurol. Neurosurg. Psychiatry.

[59] Baron J.C. (2000). The pathophysiology of migraine: Insights from functional neuroimaging. Rev. Neurol. (Paris).

[60] Aurora S.K., Welch K.M. (2000). Migraine: Imaging the aura. Curr. Opin. Neurol..

[61] James M.F., Smith J.M., Boniface S.J., Huang C.L., Leslie R.A. (2001). Cortical spreading depression and migraine: New insights from imaging?. Trends Neurosci..

[62] Dreier J.P., Kleeberg J., Petzold G., Priller J., Windmüller O., Orzechowski H.D.,  Lindauer U., Heinemann U., Einhäupl K.M., Dirnagl U. (2002). Endothelin-1 potently induces Leão’s cortical spreading depression *in vivo* in the rat: A model for an endothelin trigger of migrainous aura?. Brain.

[63] Vonderheid-Guth B., Todorova A., Wedekind W., Dimpfel W. (2000). Evidence for neuronal dysfunction in migraine: Concurrence between specific EEG findings and clinical drug response-a retrospective analysis. Eur. J. Med. Res..

[64] Eggers A.E. (2001). New neural theory of migraine. Med. Hypotheses.

[65] Gowers W.R. (1892). A Manual of Diseases of the Nervous System.

[66] Goadsby P.J. (2001). Migraine, aura and cortical spreading depression: Why are we still talking about it?. Ann. Neurol..

[67] Olesen J., Larsen B., Lauritzen M. (1981). Focal hyperemia followed by spreading oligema and impaired activation of rCBF in classic migraine. Ann. Neurol..

[68] Goadsby P.J., Lipton R.B., Ferrari M.D. (2002). Migraine-current understanding and treatment. N. Engl. J. Med..

[69] Woods R.P., Iacoboni M., Mazziota J.C. (1994). Brief report: Bilateral spreading cerebral hypoperfusion during spontaneous migraine headache. N. Engl. J. Med..

[70] Sanchez del Rio M., Bakker J., Wu O., Agosti R., Mitsikostas D.D., Ostergaard L., Wells W.A., Rosen B.R., Sorensen G., Moskowitz M.A. (1999). Perfusion weighted imaging during migraine: Spontaneous visual aura and headache. Cephalalgia.

[71] Leão A.A. (1944). Spreading depression of activity in the cerebral cortex. J. Neurophysiol..

[72] Acharya V., Acharya J., Lüders H. (1998). Olfactory epileptic auras. Neurology.

[73] Halgren E., Walter R.D., Cherlow D.G., Crandall P.H. (1978). Mental phenomena evoked by electrical stimulation of human hippocampal formation and amygdala. Brain.

[74] Herpin T.H. (1867). Des Accès Incomplets d’Epilepsie.

[75] Howe J.G., Gibson J.D. (1982). Uncinate seizures and tumors, a myth reexamined. Ann. Neurol..

[76] Kopala L.C., Good K.P., Honer W.G. (1994). Olfactory hallucinations and olfactory identification ability in patients with schizophrenia and other psychiatric disorders. Schizophr. Res..

[77] Stevenson R.J., Langdon R., McGuire J. (2011). Olfactory hallucinations in schizophrenia and schizoaffective disorder: A phenomenological survey. Psychiatry Res..

[78] Lewandowski K.E., DePaola J., Camsari G.B., Cohen B.M., Öngür D. (2009). Tactile, olfactory, and gustatory hallucinations in psychotic disorders: A descriptive study. Ann. Acad. Med. Singapore.

[79] Arguedas D., Langdon R., Stevenson R. (2012). Neuropsychological characteristics associated with olfactory hallucinations in schizophrenia. J. Int. Neuropsychol. Soc..

[80] St. John Bullen F. (1899). Olfactory hallucinations in the insane. J. Ment. Sci..

[81] Kraepelin E. Dementia Praecox and Paraphrenia (1919).

[82] Bromberg W., Schilder P. (1934). Olfactory imaginations and olfactory hallucinations. Arch. Neurol. Psychiatry.

[83] Davidson G.M. (1938). Concerning hallucinations of smell. Psychiatr. Q..

[84] Mueser K.T., Bellack A.S., Brady E.U. (1990). Hallucinations in schizophrenia. Acta Psychiatr. Scand..

[85] Assad G. (1990). Hallucinations in Clinical Psychiatry: A Guide for Mental Health Professionals.

[86] Croy I., Yarina S., Hummel T. (2013). Research Letter: Enhanced parosmia and phantosmia in patients with severe depression. Psychol. Med..

[87] Pryse-Phillips W. (1971). An olfactory reference syndrome. Acta Psychiatr. Scand..

[88] Munro A., Chmara J. (1982). Monosymptomatic hypochondriacal psychosis: A diagnostic checklist based on 50 cases of the disorder. Can. J. Psychiatry.

[89] Bishop E.R. (1980). An olfactory reference syndrome-monosymptomatic hypochondriasis. J. Clin. Psychiatry.

[90] Begum M., McKenna P.J. (2011). Olfactory reference syndrome: A systematic review of the world literature. Psychol. Med..

[91] Muffatti R., Scarone S., Gambini O. (2008). An olfactory reference syndrome successfully treated by aripiprazole augmentation of antidepressant therapy. Cogn. Behav. Neurol..

[92] Furstenburg A.C., Crosby E., Farrior B. (1943). Neurologic lesions which influence the sense of smell. Arch. Otolaryngol..

[93] Allen I.M. (1944). Spontaneous olfactory and gustatory phenomena with and without organic lesions of the brain. N. Z. Med. J..

[94] Mulder D.W., Daly D. (1952). Psychiatric symptoms associated with lesions of temporal lobe. J. Am. Med. Assoc..

[95] Hollander E., Neville D., Frenkel M., Josephson S., Liebowitz M.R. (1992). Body dysmorphic disorder. Diagnostic issues and related disorders. Psychosomatics.

[96] Harriman P.L. (1934). A case of olfactory hallucinations in a hypochrondriacal prisoner. J. Abnorm. Soc. Psychol..

[97] Mizuo T., Ando M. (1988). A case of olfactory hallucinations with adult enuresis. Hinyokika Kiyo.

[98] Kellner C.H., Bachman D.L. (1992). Olfactory hallucination after intravenous caffeine. Am. J. Psychiatry.

[99] Koinigsburg H.W., Pollak C.P., Fine J. (1993). Olfactory hallucinations after the infusion of caffeine during sleep. Am. J. Psychiatry.

[100] Kroemer S., Kawohl W. (2011). Gustatory and olfactory hallucinations under therapeutic dosing of bupropion. J. Neuropsychiatry Clin. Neurosci..

[101] Schechter P.J., Henkin R.I. (1974). Abnormalities of taste and smell after head trauma. J. Neurol. Neurosurg. Psychiatry.

[102] Henkin R.I., English G.M. (1993). Evaluation and Treatment of Human Olfactory Dysfunction. Otolaryngology.

[103] Henkin R.I. (1994). Drug induced taste and smell disorders: Incidence, mechanisms and management related primarily to treatment of sensory receptor dysfunction. Drug Saf..

[104] Levy L.M., Henkin R.I. (2000). Physiologically initiated and inhibited phantosmia: Cyclic unirhinal, episodic, recurrent phantosmia revealed by brain fMRI. J. Comput. Assist. Tomogr..

[105] Henkin R.I., Levy L.M., Fordyce A., Henkin R.I., Levy L.M., Fordyce A. (2013). Taste and smell function in chronic disease: A review of clinical and biochemical evaluation of taste and smell dysfunction in over 5000 patients at The Taste and Smell Clinic in Washington, DC. Am. J. Otolaryngol..

[106] Lin S.H., Chu S.T., Yuan B.C., Shu C.H. (2009). Survey of the frequency of olfactory dysfunction in Taiwan. J. Chin. Med. Assoc..

[107] Hong S.C., Holbrook E.H., Leopold D.A., Hummel T. (2012). Distorted olfactory perception: A systematic review. Acta Otolaryngol..

[108] Chen C., Shih Y.H., Yen D.J., Lirng J.F., Guo Y.C., Yu H.Y., Yiu C.H. (2003). Olfactory auras in patients with temporal lobe epilepsy. Epilepsia.

[109] Mizobuchi M., Ito N., Tanaka C., Sako K., Sumi Y., Sasaki T. (1999). Unidirectional olfactory hallucination associated with ipsilateral unruptured intracranial aneurysm. Epilepsia.

[110] ye E., Arendts G. (2002). Intracerebral haemorrhage presenting as olfactory hallucinations. Emerg. Med. (Fremantle).

[111] Capampangan D.J., Hoerth M.T., Drazkowski J.F., Lipinski C.A. (2010). Olfactory and gustatory hallucinations presenting as partial status epilepticus because of glioblastoma multiforme. Ann. Emerg. Med..

[112] Kumar G., Juhász C., Sood S., Asano E. (2012). Olfactory hallucinations elicited by electrical stimulation via subdural electrodes: Effects of direct stimulation of olfactory bulb and tract. Epilepsy Behav..

[113] DiFabio R., Casali C., Giugni E., Pierelli F. (2009). Olfactory hallucinations as a manifestation of hidden rhinosinusitis. J. Clin. Neurosci..

[114] Landis BN., Burkhard P.R. (2008). Phantosmias and Parkinson disease. Arch. Neurol..

[115] Bannier S., Berdagué J.L., Rieu I., de Chazeron I., Marques A., Derost P., Ulla M., Llorca P.M., Durif F. (2012). Prevalence and phenomenology of olfactory hallucinations in Parkinson’s disease. J. Neurol. Neurosurg. Psychiatry.

[116] Tousi B., Frankel M. (2004). Olfactory and visual hallucinations in Parksinson’s disease. Parkinsonism Relat. Disord..

[117] Brundin P., Petit G. (2011). Use of Rasagiline for the Treatment of Olfactory Dysfunction. U.S. Patent.

[118] Yang J.C., Khakoo Y., Lightner D.D., Wolden S.L. (2013). Phantosmia during radiation therapy: A report of 2 cases. J. Child Neurol..

[119] Leopold D.A., Loehrl T.A., Schwob J.E. (2002). Long-term follow-up of surgically treated phantosmia. Arch. Otolaryngol. Head Neck Surg..

[120] Markert J.M., Hartshorn D.O., Farhat S.M. (1993). Paroxysmal bilateral dysosmia treated by resection of the olfactory bulbs. Surg. Neurol..

[121] Landis B.N., Croy I., Haehner A. (2012). Long lasting phantosmia treated with venlafaxine. Neurocase.

[122] Johnson J., Bourgeois J.A., Quanbeck C. (2006). Treatment of olfactory hallucinations with topiramate. J. Clin. Psychopharmacol..

[123] Landis B.N., Reden J., Haehner A. (2010). Idiopathic phantosmia: Outcome and clinical significance. ORL J. Otorhinolaryngol. Relat. Spec..

[124] Brown R.J., Bouska J.F., Frow A., Kirkby A., Baker G.A., Kemp S., Burness C., Reuber M. (2013). Emotional dysregulation, alexithymia, and attachment in psychogenic nonepileptic seizures. Epilepsy Behav..

[125] Xue Q., Wang Z.Y., Xiong X.C., Tian C.Y., Wang Y.P., Xu P. (2013). Altered brain connectivity in patients with psychogenic non-epileptic seizures: A scalp electroencephalography study. J. Int. Med. Res..

[126] Henkin R.I. (1987). Report on a survey on smell in the US. Olfactory Rev..

[127] Ikui A., Ikeda M., Tahara I., Ito I., Endo S., Kurihara K., Suzuki N., Ogawa H. (1994). Clinical Aspects of Phantogeusia. Olfaction and Taste XI.

[128] Henkin R.I. (1971). Disorders of taste and smell. JAMA.

[129] Anderson J.  (1886). On sensory epilepsy. A case of basal cerebral tumour affecting the left tempero-sphenoidal lobe, and giving rise to a paroxysmal taste-sensation and dreamy state. Brain.

[130] Henkin R.I., Kurihara K., Suzuki N., Ogawa H. (1994). Concepts of Therapy in Taste and Smell Dysfunction: Repair of Sensory Receptor Function as Primary Treatment. Olfaction and Taste XI.

[131] Beeman C.E. (1889). A case of epilepsy with olfactory aura from a tumour in the tempero-sphenoidal lobe. Br. Med. J..

[132] Clarke J.M. (1900). Epileptic attacks preceded by subjective auditory and taste sensations, probably due to tumour of the left tempero-sphenoidal lobe. Lancet.

[133] Friedman H. (1909). Taste hallucinations in cerebral tumor. Wien Klin Rundsch..

[134] Feré C. (1896). Note sur les sensations subjectives de l’odorat chez un épileptique. Compt. Rendus Soc. Biol..

[135] Henkin R.I., Taylor R.B. (1992). Phantageusia. Difficult Diagnosis II.

[136] Toulouse E., Vaschide N. (1899). Influence des crises épileptiques sur l’olfaction. Compt. Rendus Soc.Biol..

[137] Gastaut H., Roger J., Giove C. (1955). Troubles de l’olfaction, de la gustation et de l’appetit chez les épileptiques psychomoteurs. Ann. Med. Psychol..

[138] Bartlet J.E. (1951). A case of organized visual hallucinations in an old man with cataract and their relation to the phenomena of the phantom limb. Brain.

[139] Podoll K., Osterheider M., Noth J. (1989). The Charles Bonnet syndrome. Fortschr. Neurol. Psychiatr..

[140] Pliskin N.H., Kiolbasa T.A., Towle V.L., Pankow L., Ernest J., Noronha A, Luchins D.J. (1996). Charles Bonnet syndrome: An early marker for dementia?. J. Am. Geriatr. Soc..

[141] Teunisse R.J., Cruysberg J.R., Verbeek A., Zitman F.G. (1995). The Charles Bonnet syndrome: A large prospective study in the Netherlands. Br. J. Psychiatry.

[142] Teunisse R.J., Cruysberg J.R., Hoefnagels W.H., Verbeek A.L., Zitman F.G. (1996). Visual hallucinations in psychologically normal people: Charles Bonnet’s syndrome. Lancet.

[143] Teunisse R.J., Cruysberg J.R., Hoefnagels W.H., van’t Hof M.A., Verbeek A.L., Zitman F.G. (1998). Risk indicators for the Charles Bonnet Syndrome. J. Nerv. Ment. Dis..

[144] De Ajuriaguerra J., LeGenne J. (1946). Unilateral auditory and visual hallucinations in a hard of hearing blind man with deafness in the right ear. Ann. Med. Psychol. (Paris).

[145] Hécaen H., Ropert R. (1959). Auditory hallucinations in the course of neurologic syndromes. Ann. Med. Psychol. (Paris).

[146] Lennox G. (1988). Auditory hallucinations due to ear disease. Br. J. Psychiatry.

[147] Toulouse E. (1892). Unilateral hallucinations. Gazette Hôpitaux.

[148] Chamorro A., Sacco R.L., Ciecierski K., Binder J.R., Tatemichi T.K., Mohr J.P. (1990). Visual hemineglect and hemihallucinations in a patient with a subcortical infarction. Neurology.

[149] Bergman P.S. (1965). Unilateral auditory hallucinations. Trans. Am. Neurol. Assoc..

[150] Hauptman B., Glick I.D. (1968). Auditory hallucinations with imipramine. J. Hillside Hosp..

[151] Gordon A.G. (1988). Unilateral auditory hallucinations. Br. J. Psychiatry.

[152] Almeida O.P., Forstl H., Howard R., David A.S. (1993). Unilateral auditory hallucinations. Br. J. Psychiatry.

[153] Bremer J. (1996). New onset auditory hallucinations after right temporal lobectomy. Am. J. Psychiatry.

[154] Gordon A.G. (1997). Unilateral auditory hallucinations: Ear or brain?. J. Neurol. Neurosurg. Psychiatry.

[155] Potolicchio S.J., Lossing J.H., O’Doherty D.S., Henkin R.I. (1986). Partial seizures with simple psychosensory symptomatology (cyclic phantosmia): A new and distinct seizure disorder. Clin. Res..

[156] Levy L.M., Hutter A., Schroder J., Lin C.S., Schellinger D., Henkin R.I. (1997). Brain localization of cyclic unilateral olfactory phantoms by functional magnetic resonance imaging (fMRI) by treatment with valproic acid and thioridazine. J. Invest. Med..

[157] Henkin R.I., Larson A.L., Powell R.D. (1975). Hypogeusia, dysgeusia, hyposmia and dysosmia following influenza-like infection. Ann. Otol. Rhinol. Laryngol..

[158] Church J.A., Bauer H., Bellanti J.A., Satterly R.A., Henkin R.I. (1978). Hyposmia associated with atopy. Ann. Allergy.

[159] Henkin R.I. (1973). Hyposmia and hypogeusia due to nonallergic rhinitis. J. Am. Med. Assoc..

[160] Henkin R.I., Velicu I., Schmidt L. (2009). An open-label controlled trial of theophylline for treatment of patients with hyposmia. Am. J. Med. Sci..

[161] Henkin R.I., Schultz M., Minnick-Poppe L. (2012). Intranasal theophylline treatment of patients with hyposmia and hypogeusia: A pilot study. Arch. Otolaryngol. Head Neck Surg..

[162] Levy L.M., Henkin R.I., Lin C.S., Hutter A., Schellinger D. (1999). Odor memory induces brain activation as measured by functional MRI. J. Comput. Assist. Tomogr..

[163] Levy L.M., Henkin R.I., Lin C.S., Finley A., Schellinger D. (1999). Taste memory induces brain activation as revealed by functional MRI. J. Comput. Assist. Tomogr..

[164] Levy L.M., Hallett M. (2002). Impaired brain GABA in focal dystonia. Ann. Neurol..

[165] Henkin R.I., Potolicchio S.J., Levy L.M. (2011). Improvement in smell and taste dysfunction after repetitive transcranial magnetic stimulation. Am. J. Otolaryngol..

[166] Henkin R.I., Potolicchio S.J., Levy L.M. (2002). Rapid changes in taste and smell function following transcranial magnetic stimulation (TCMS) in humans: Relationships to CNS plasticity. FASEB J..

[167] Henkin R.I., Potolicchio S.J., Levy L.M., Moharram R., Velicu I., Martin B.M. (2010). Carbonic anhydrase (CA) I, II and VI, blood plasma, erythrocyte and saliva zinc and copper increase after repetitive transcranial magnetic stimulation. Am. J. Med. Sci..

[168] Herron J. (1980). Neuropsychology of Left-Handedness.

[169] McManus I.C., Bryden M.C., Rapin I., Segalowitz S.J. (1992). The Genetics of Handedness, Cerebral Dominance and Lateralization. Handbook of Neuropsychology.

[170] Katzman G.L., Dagher A.P., Patronas N.J. (1999). Incidental findings on brain magnetic resonance imaging from 1000 asymptomatic volunteers. JAMA.

[171] Maly P.V., Sundgren P.C. (1995). Changes in paranasal sinus abnormalities found incidentally on MR. Neuroradiology.

[172] Patel K., Chvada S.V., Violaris N., Pahor A.L. (1996). Incidental paranasal sinus inflammatory changes in a British population. J. Laryngol. Otol..

[173] Iwabuchi Y., Hanamure Y., Hirota J., Ohyama M. (1997). Clinical significance of asymptomatic sinus abnormalities on magnetic resonance imaging. Arch. Otolaryngol. Head Neck Surg..

[174] Kurz A., Sessler D.I., Lenhardt R. (1996). Perioperative normothermia to reduce the incidence of surgical-wound infection and shorten hospitalization. Study of Wound Infection and Temperature Group. N. Engl. J. Med..

[175] Henkin R.I., Levy L.M. (2001). Lateralization of brain activation to imagination and smell of odors using functional magnetic resonance imaging (fMRI): Left hemispheric localization of pleasant and right hemispheric localization of unpleasant odors. J. Comput. Assist. Tomogr..

[176] Greif R., Akça O., Horn E.P., Kurz A., Sessler D.I., Outcomes Research Group (2000). Supplemental perioperative oxygen to reduce the incidence of surgical-wound infection. N. Engl. J. Med,.

[177] Gottrup F., Firmin R., Rabkin J., Halliday B.J., Hunt T.K. (1987). Directly measured tissue oxygen tension and arterial oxygen tension assess tissue perfusion. Crit. Care Med..

[178] Gottrup F. (2000). Prevention of surgical-wound infections. N. Engl. J. Med..

[179] Levy L.M., Henkin R.I., Lin C.S., Hutter A., Schellinger D. (1998). Increased brain activation in response to odors in patients with hyposmia after theophylline treatment demonstrated by fMRI. J. Comput. Assist. Tomogr..

[180] Sheehy M.P., Marsden C.D. (1982). Writers’ cramp-a focal dystonia. Brain.

[181] Berardelli A., Rothwell J.C., Hallett M., Thompson P.D., Manfredi M., Marsden C.D. (1998). The pathophysiology of primary dystonia. Brain.

[182] Byl N.N., McKenzie A., Nagarajan S.S. (2000). Differences in somatosensory hand organization in a healthy flutist and a flutist with focal hand dystonia: A case report. J. Hand Ther..

[183] Tinazzi M., Priori A., Bertolasi L., Frasson E., Mauguière F., Fiasche A. (2000). Abnormal central integration of a dual somatosensory input in dystonia. Evidence for sensory overflow. Brain.

[184] Priori A., Pesenti A., Cappellari A., Scarlato G., Barbieri S. (2001). Limb immobilization for the treatment of focal occupational dystonia. Neurology.

[185] Pascual-Leone A. (2001). The brain that plays music and is changed by it. Ann. N. Y. Acad. Sci..

[186] Matsumura M., Sawaguchi T., Oishi T., Ueki K., Kubota K. (1991). Behavioral deficits induced by local injection of bicuculline and muscimol into the primate motor and premotor cortex. J. Neurophysiol..

[187] Löscher W., Hörstermann D. (1992). Abnormalities in amino acid neurotransmitters in discrete brain regions of genetically dystonic hamsters. J. Neurochem..

[188] De Yebenes J.G., Vazquez A., Martinez A., Mena M.A., del Rio R.M., de Felipe C., del Rio J. (1988). Biochemical findings in symptomatic dystonias. Adv. Neurol..

[189] Richter A., Löschmann P.A., Löscher W. (1994). The novel antiepileptic drug, lamotrigine, exerts prodystonic effects in a mutant hamster model of generalized dystonia. Eur. J. Pharmacol..

[190] Messer A. (1982). Amino acid changes in the mouse mutant dystonia musculorum similar to those in Friedreich’s ataxia. Can. J. Neurol. Sci..

[191] Leopold D.A., Schwob J.E., Youngentob S.L., Hornung D.E., Wright H.N., Mozell M.M. (1991). Successful treatment of phantosmia with preservation of olfaction. Arch. Otolarngol. Head Neck Surg..

[192] Pujol J., Roset-Llobet J., Rosinés-Cubells D., Deus J., Narberhaus B., Valls-Solé J., Capdevila A., Pascual-Leone A. (2000). Brain cortical activation during guitar-induced hand dystonia studied by functional MRI. Neuroimage.

[193] Tanaka C. (1985). Gamma-aminobutyric acid in peripheral tissues. Life Sci..

[194] Pertwee R.G., Wickens A.P. (1991). Enhancement by chloridiazepoxide of catalepsy induced in rats by intravenous or intrapallidal injections of enantiomeric cannabinoids. Neuropharmacology.

[195] Romero J., Garcia-Palomero E., Fernández-Ruiz J.J., Ramos J.A. (1996). Involvement of GABA(B) receptors in the motor inhibition produced by agonists of brain cannabinoid receptors. Behav. Pharmacol..

[196] Coull M.A., Johnston A.T., Pertree R.G., Davies S.N. (1997). Action of delta-9-tetrahydrocannabinal on GABA(A) receptor mediated responses in a grease-gap recording preparation of the rat hippocampal slice. Neuropharmacology.

[197] Garcia-Gil L., de Miguel R., Romero J., Perez A., Ramos J.A., Fernández-Ruiz J.J. (1999). Perinatal delta-9-tetrahydrocannabinol exposure augmented the magnitude of motor inhibition caused by GABA(B), but not GABA(A), receptor agonists in adult rats. Neurotoxicol. Teratol..

[198] Gale K. (1980). Chronic blockade of dopamine receptors by antischizophrenic drugs enhances GABA binding in substantia nigra. Nature.

[199] Lawrence L.J., Geen K.W., Yamamura H.I., Olsen R.W., Venter C.J. (1986). GABA Involvement in Benzodiazepine Receptor Modulation of the Chloride Ionophore. Benzodiazepine/GABA Receptors and Chloride Channels: Structural and Functional Properties.

[200] Ey H. (1973). Traité des Hallucinations.

[201] Mantegazza P., Andrews G., Solomon D. (1975). On the Hygienic and Medicinal Virtues of Coca. The Coca Leaf and Cocaine Papers.

[202] Siegel R.K. (1978). Cocaine hallucinations. Am. J. Psychiatry.

[203] Dittrich A., Bickel P., Schöpf J., Zimmer D. (1976). Comparison of altered states of consciousness induced by the hallucinogens (−)-delta9-*trans*-tetrahydrocannabinol (delta9-THC) and *N*,*N*-dimethyltryptamine (DMT) (author’s transl). Arch. Psychiatr. Nervenkr..

[204] Koukkou M., Lehmann D. (1976). Human EEG spectra before and during cannibis hallucinations. Biol. Psychiatry.

[205] Sorg B.A., Guminski B.J., Hooks M.S., Kalivas P.W. (1995). Cocaine alters glutamic acid decarboxylase differentially in the nucleus accumbens core and shell. Brain Res. Mol. Brain Res..

[206] Peris J., Jung B.J., Resnick A., Walker P., Malakhova O., Bokrand Y., Wielbo D. (1998). Antisense inhibition of striatal GABA_A_ receptor proteins decreases GABA-stimulated chloride uptake and increases cocaine sensitivity in rats. Brain Res. Mol. Brain Res..

[207] Peris J. (1996). Repeated cocaine injections decrease the function of striatal gamma-aminobutyric acid (A) receptors. J. Pharmacol. Exp. Ther..

[208] Ye J.H., Liu P.L., Wu W.H., McArdle J.J. (1997). Cocaine depresses GABA_A_ current of hippocampal neurons. Brain Res..

[209] Giorgetti M., Javaid J.I., Davis J.M., Costa E., Guidotti A., Appel S.B., Brodie M.S. (1998). Imidazenil, a positive allosteric GABA_A_ receptor modulator, inhibits the effects of cocaine on locomotor activity and extracellular dopamine in the nucleus accumbens shell without tolerance liability. J. Pharmacol. Exp. Ther..

[210] Isbell H., Gorodetzsky C.W., Jasinski D., Claussen U., von Spulak F., Korte F. (1967). Effects of (−)-delta-9-*trans*-tetrahydrocannabinol in man. Psychopharmacologia.

[211] Berrios G.E. (1982). Tactile hallucinations: Conceptual and historical aspects. J. Neurol. Neurosurg. Psychiatry.

[212] Bekavac I., Waterhouse B.D. (1995). Systemically administered cocaine selectively enhances long-latency responses of rat barrel field cortical neurons to vibrissae stimulation. J. Pharmacol. Exp. Ther..

[213] Ziemann U., Hallett M., Cohen L.G. (1998). Mechanisms of deafferentation-induced plasticity in human motor cortex. J. Neurosci..

[214] Yamanaka T., Him A., Cameron S.A., Dutia M.B. (2000). Rapid compensatory changes in GABA receptor efficacy in rat vestibular neurons after unilateral labyrinthectomy. J. Physiol..

[215] Donaghue J.P., Hess G., Sanes J.N., Bloedel J.R., Ebner T.J., Wise S.P. (1996). Substrates and Mechanisms for Learning in Motor Cortex. The Acquisition of Motor Behavior in Vertebrates.

[216] Huntley G.W. (1997). Correlation between patterns of horizontal connectivity and the extent of short-term representational plasticity in rat motor cortex. Cereb. Cortex.

[217] Capaday C., Richardson M.P., Rothwell J.C., Brooks D.J. (2000). Long-term changes of GABAergic function in the sensorimotor cortex of amputees. A combined magnetic stimulation and 11C-flumazenil PET study. Exp. Brain Res..

[218] Hendry S.H., Jones E.G. (1986). Reduction in number of immunostained GABAergic neurons in deprived-eye dominance columns of monkey area 17. Nature.

[219] Hendry S.H., Jones E.G. (1988). Activity-dependent regulation of GABA expression in the visual cortex of adult monkeys. Neuron.

[220] Rosier A.M., Arckens L., Demeulemeester H., Orban G.A., Eysel U.T., Wu Y.J., Vandesande F. (1995). Effect of sensory deafferentation on immunoreactivity of GABAergic cells and on GABA receptors in the adult cat visual cortex. J. Comp. Neurol..

[221] Welker E., Soriano E., van der Loss H. (1989). Plasticity in the barrel cortex of the adult mouse: Effects of peripheral deprivation on GAD-immunoreactivity. Exp. Brain Res..

[222] Cameron D.L., Williams J.T. (1994). Cocaine inhibits GABA release in the VTA through endogenous 5-HT. J. Neurosci..

[223] Dewey S.L., Chaurasia C.S., Chen C.E., Volkow N.D., Clarkson F.A., Porter S.P., Straughter-Moore R.M., Alexoff D.L., Tedeschi D., Russo N.B. (1997). ABAergic attenuation of cocaine-induced dopamine release and locomotor activity. Synapse.

[224] Morgan A.E., Dewey S.L. (1998). Effects of pharmacologic increases in brain GABA levels on cocaine-induced changes in extracellular dopamine. Synapse.

[225] Shoaib M., Swanner L.S., Beyer C.E., Goldberg S.R., Schindler C.W. (1998). The GABAB agonist baclofen modifies cocaine self-administration in rats. Behav. Pharmacol..

[226] Suzuki T., Abe S., Yamaguchi M., Baba A., Hori T., Shiraishi H., Ito T. (2000). Effects of cocaine administration on receptor binding and subunits mRNA of GABA(A)-benzodiazepine receptor complexes. Synapse.

[227] Lilly S.M., Tietz E.I. (2000). Chronic cocaine differentially affects diazepam’s anxiolytic and anticonvulsant actions. Relationship to GABA(A) receptor subunit expression. Brain Res..

[228] Abel M.S., Kohli N. (1999). GABA-transaminase antisense oligodeoxynucleotide modulates cocaine- and pentylenetetrazol-induced seizures in mice. Metab. Brain Dis..

[229] De Leon K.R., Todtenkopf M.S., Stellar J.R. (2000). An examination of glutamate decarboxylase (65) immunoreactive puncta with respect to rat ventral pallidum neurons after repeated cocaine administration. Neurosci. Lett..

[230] Ali S.F., Newport G.D., Scallet A.C., Gee K.W., Paule M.G., Brown R.M., Slikker W. (1989). Effects of chronic delta-9-tetrahydrocannabinol (THC) administration on neurotransmitter concentrations and receptor binding in the rat brain. Neurotoxicology.

[231] Bonci A., Williams J.T. (1996). A common mechanism mediates long-term changes in synaptic transmission after chronic cocaine and morphine. Neuron.

[232] Neugebauer V., Zinebi F., Russell R., Gallagher J.P., Shinnick-Gallagher P. (2000). Cocaine and kindling alter the sensitivity of group II and III metabotropic glutamate receptors in the central amygdala. J. Neurophysiol..

[233] Messer A., Gordon D. (1979). Changes in whole tissue biosynthesis of gamma-amino butyric acid (GABA) in basal ganglia of the dystonia (dtAlb) mouse. Life Sci..

[234] Richter A., Löscher W. (1999). Gabapentin decreases the severity of dystonia at low doses in a genetic animal model of paroxysmal dystonic choreoathetosis. Eur. J. Pharmacol..

[235] Weber O.M., Verhagen A., Duc D.O., Meier D., Leenders K.L., Boesiger P. (1999). Effects of vigabatrin intake on brain GABA activity as monitored by spectrally edited magnetic resonance spectroscopy and positron emission tomography. Magn. Reson. Imaging.

[236] Greene P. (1992). Baclofen in the treatment of dystonia. Clin. Neuropharmacol..

[237] Petroff O.A., Rothman D.L., Behar K.L., Mattson R.H. (1996). Low brain GABA level is associated with poor seizure control. Ann. Neurol..

[238] Siebner H.R., Tormos J.M., Ceballos-Baumann A.O., Auer C., Catala M.D., Conrad B., Pascual-Leone A. (1999). Low frequency repetitive transcranial magnetic stimulation of the motor cortex in writer’s cramp. Neurology.

[239] Pascual-Leone A., Valls-Sole J., Wassermann E.M., Hallett M. (1994). Responses to rapid-rate transcranial magnetic stimulation of the human motor cortex. Brain.

[240] Hoshiyama M., Kakige R. (1999). Shortening of the cortical silent period following transcranial magnetic brain stimulation during an experimental paradign for generating contingent negative variation (CNV). Clin. Neurophysiol..

[241] Sommer M., Tergau F., Wischer S., Reimers C.D., Beuche W., Paulus W. (1999). Riluzole does not have an acute effect on motor thresholds and the intracortical excitability in amyotrophic lateral sclerosis. J. Neurol..

[242] Ben-Shacher D., Gazawi H., Riboyad-Levin J., Klein E. (1999). Chronic repetitive transcranial magnetic stimulation alters beta adrenergic and 5-HT2 receptor characteristics in rat brain. Brain Res..

[243] Wu T., Sommer M., Tergau F., Paulus W. (2000). Lasting influence of repetitive transcranial magnetic stimulation on intracortical excitability in human subjects. Neurosci. Lett..

[244] Terao Y., Hayashi H., Shimizu T., Tanabe H., Hanajima R., Ugawa Y. (1995). Altered motor cortical excitability to magnetic stimulation in a patient with a lesion in the globus pallidus. J. Neurol. Sci..

[245] Siebner H.R., Dressmandt J., Auer C., Conrad B. (1998). Continuous tracheal baclofen infusions induced a marked increase of the transcranially evoked silent period in a patient with generalized dystonia. Muscle Nerve.

[246] Hamilton W.F., Woodbury R.A., Harper H.T. (1936). Physiologic relationships between intrathoracic, intraspinal and arterial pressure. JAMA.

[247] Nishimura R.A., Tajik A.J. (1986). The valsalva maneuver and response revisited. Mayo Clin. Prac..

[248] Hamilton W.F., Woodbury R.A., Harper H.T. (1944). Arterial, cerebrospinal and venous pressures in man during cough and strain. Am. J. Physiol..

[249] Bellet S. (1972). Essentials of Cardiac Arrhythmias: Diagnosis and Management.

[250] Waxman M.B., Wald R.W., Finley J.P., Bonet J.F., Downar E., Sharma A.D. (1980). Valsalva termination of ventricular tachycardia. Circulation.

[251] Levine H.J., McIntyre K.M., Glovsky M.M. (1966). Relief of angina pectoris by Valsalva maneuver. N. Engl. J. Med..

[252] Pepine C.J., Wiener L. (1971). Clinical and hemodynamic responses to the Valsalva maneuver during angina pectoris (abstract). Circulation.

[253] Burcharth F., Bertheussen K. (1979). The influence of posture, Valsalva maneuver and coughing on portal hypertension in cirrhosis. Scand. J. Clin. Lab. Invest..

[254] Jankovic G.M. (1980). Factor VIII response to a Valsalva manoeuvre. Br. J. Haematol..

[255] Ogawa S., Lee T.M., Nayak A.S., Glynn P. (1990). Oxygenation-sensitive sensory contrast in magnetic resonance image of rodent brain at high magnetic fields. Mag. Reson. Med..

[256] Kwong K.K., Belliveau J.W., Chesler D.A., Goldberg I.E., Weiskoff R.M., Poncelet B.P., Kennedy D.N., Hoppel B.E., Cohen M.S., Turner R. (1992). Dynamic magnetic resonance imaging of human brain activity during primary sensory stimulation. Proc. Natl. Acad. Sci. USA.

[257] Lempert T., Bauer M., Schmidt D. (1994). Syncope: A videometric analysis of 56 episodes of transient cerebral hypoxia. Ann. Neurol..

[258] Duvoisin R.C. (1962). Convulsive syncope induced by the Weber maneuver. Arch. Neurol..

[259] Whinnery J.E., Whinnery A.M. (1990). Acceleration-induced loss of consciousness. A review of 500 episodes. Arch. Neurol..

[260] Harper R.M., Gozal D., Bandler R., Spriggs D., Lee J., Alger J. (1998). Regional brain activation in humans during respiratory and blood pressure challenges. Clin. Exp. Pharmacol. Physiol..

[261] Posse S., Olthoff U., Weckesser M., Jäncke-Müller-Gärtner H.W., Dager S.R. (1997). Regional dynamic signal changes during controlled hyperventilation assessed with blood oxygen level-dependent functional MR imaging. Am. J. Neuroradiol..

[262] Roberts E., Adelman G., Smith B.H. (1999). Gamma-Aminobutyric Acid (GABA). Encyclopedia of Neuroscience.

[263] Morimoto K., Goddard G.V. (1986). Kindling induced changes in EEG recorded during stimulation from the site of stimulation: Collapse of GABA-mediated inhibition and onset of rhythymic synchronous burst. Exp. Neurol..

[264] Harris J.K., DeLorenzo R.J., Smith D.B. (1990). Epilepsy-A Molecular and Cellular Views. Epilepsy: Current Approaches to Diagnosis and Treatment.

[265] McNamara J.O., Bonhaus D.W., Shin C., Crain B.J., Gellman R.L., Giacchino J.L. (1985). The kindling model of epilepsy, a critical review. CRC Crit. Rev. Clin. Neurobiol..

[266] Tsura N. (1985). Neuronal firing pattern following amygdaloid kindling in unrestrained rats. Epilepsia.

[267] Wasterlain C.G., Morin A.M., Fujikawa D.G., Bronstein J.M., Bolwig T.G., Trimble M.R. (1989). Chemical Kindling. The Clinical Relevance of Kindling.

[268] Goddard G., McIntyre D., Leech C.K. (1969). A permanent change in brain function resulting from daily electrical stimulation. Exp. Neurol..

[269] Goddard G.V. (1983). The kindling model of epilepsy. Trends Neurosci..

[270] Morrell F. (1991). Kindling and Synaptic Plasticity: The Legacy of Graham Goddard.

[271] Burchfiel J.L., Appelgate C.D., Samoriski G.M., Nierenberg J., Corcoran M.E., Moshe S.L. (1998). The Role of Rhinencephalic Networks in Early Stage Kindling. Kindling 5 Advances in Behavioral Biology.

[272] Appelgate C.D., Burchfiel J.L., Ferland R.J., Nierenberg J., Corcoran M.E., Moshe S.L. (1998). The Role of Rhinencephalic Networks in the Late Stages of Kindling. Kindling 5 Advances in Behavioral Biology.

[273] McIntyre D.C., Kelly M.E., Corcoran M.E., Moshe S.L. (1998). The Perirhinal Cortex and Kindled Motor Seizures. The Role of Rhinencephalic Networks in Early Stage Kindling. Kindling 5 Advances in Behavioral Biology.

[274] Corcoran M.E., Wada J.A. (1981). Catecholamines and Kindling. Kindling 2.

[275] Vezzani A., Piwko C., Gobbi M., Schwarzer C., Sperk G., Hoyer D., Corcoran M.E., Moshe S.L. (1998). Somatostatin-and Neuropeptide y-Mediated Neurotransmission in Kindling Epileptogenesis. Kindling 5 Advances in Behavioral Biology.

[276] Nillni E.A., Sevarino K.A. (1999). The biology of prothyrotropin-releasing hormone-derived peptides. Endocr. Rev..

[277] Holmes G.L., Weber D.A. (1985). Effects of hypoxic-ischemic encephalopathies on kindling in the immature brain. Exp. Neurol..

[278] Moshe S.L., Albala B.J. (1985). Perinatal hypoxia and subsequent development of seizures. Physiol. Behav..

[279] Vion-Dury J., Rougier I., LeGal LaSalle G., Bras J., Papy J.J. (1985). Epileptogenesis caused by the kindling effect in diving at 3 ATA of air. J. Physiol. (Paris).

[280] Vion-Dury J., LeGal LaSalle G., Rougier I., Papy J.J. (1986). Effects of hyperbaric and hyperoxic conditions on amygdala kindled seizures in rat. Exp. Neurol..

[281] Frantseva M.V., Perez Velazquez J.L., Tsoraklidis G., Mendonca A.J., Adamchik Y., Mills L.R., Carlen P.L., Burnham M.W. (2000). Oxidative stress is involved in seizure induced neurodegeneration in the kindling model of epilepsy. Neuroscience.

[282] Laffey J.G., Kavanaugh B.P. (1999). Carbon dioxide and the critically ill—Too little of a good thing?. Lancet.

[283] Lennox W.G., Gibbs F.A., Gibbs E.L. (1938). The relationship in man of cerebral activity to blood flow and to blood constituents. J. Neurol. Psychiatry.

[284] Woodbury D.M., Rollins L.T., Cardner M.O., Hirschi W.L., Hogan J.R., Rallison M.L., Tanner G.S., Brodie D.A. (1957). Effects of carbon dioxide on brain excitability and electrolytes. Am. J. Physiol..

[285] Bird J.M., Engel J., Padley T.A. (1997). Hyperventilation and Panic Attacks. Epilepsy: A Comprehensive Textbook.

[286] Meduna L.J., Gyarfas K. (1949). Motor and sensory phenomena during CO_2_ inhalations. Dis. Nerv. Syst..

[287] Gyarfas K., Pollack G.H., Stein S.N. (1949). Central inhibitory effects of carbon dioxide, IV. Convulsive phenomena. Exp. Biol. Med..

[288] Leake C.D., Waters R.M. (1929). The anesthetic properties of carbon dioxide. Anesth. Analg..

[289] Seevers M.H. (1944). The narcotic properties of carbon dioxide. N. Y. State J. Med..

[290] Poulsen T. (1952). Investigations into the anesthetic properties of carbon dioxide. Acta Pharmacol. Toxicol..

[291] Gibbs F.A., Gibbs E.L., Lennox W.G. (1938). Cerebral dysrhythmias of epilepsy. Arch. Neurol. Psychiatry.

[292] Clowes G.H.A., Hopkins A.L., Simeone F.A. (1955). A comparison of the physiological effects of hypercapnia and hypoxia in the production of cardiac arrest. Ann. Surg..

[293] Roth M., Harper M. (1962). Temporal lobe epilepsy and the phobic anxiety-depersonalization syndrome. II. Practical and theoretical considerations. Compr. Psychiatry.

[294] Holmberg G. (1953). The electroencephalogram during hypoxia and hyperventilation. Electroencephalogr. Clin. Neurophysiol..

[295] Wyke B. (1963). Brain Function and Metabolic Disorders: The Neurological Effects of Changes in Hydrogen ion Concentration.

[296] Bellodi L., Perna G., Caldirola D., Arancio C., Bertani A., Di Bella D. (1998). CO_2_ induced panic attacks: A twin study. Am. J. Psychiatry.

[297] Papp L.A., Marinez J.M., Kelin D.F., Coplan J.D., Norman R.G., Cole R., de Jesus M.J., Ross D., Goetz R., Gorman J.M. (1997). Respiratory psychopathology of panic disorder: Three respiratory challenges in 98 subjects. Am. J. Psychiatry.

[298] Roy-Byrne P.P., Cowley D.S. (1998). Search for pathophysiology of panic disorder. Lancet.

[299] Brown E.B. (1953). Physiological effects of hyperventilation. Physiol. Rev..

[300] Magarian G.J., Olney R.K. (1984). Absence spells. Hyperventilation syndrome as a previously unrecognized cause. Am. J. Med..

[301] Stillman A.E., Hu X., Jerosch-Herold M. (1995). Functional MRI of brain during breath holding at 4T. Magn. Reson. Imaging.

[302] Kwong K.K., Wanke I., Donahue K.M., Dass T.L., Rosen B.R. (1995). EPI imaging of global increase in brain MR signal with breath-hold preceded by breathing O_2_. Magn. Reson. Med..

[303] Moritz C.H., Meyerand M.E., Saykin A.J. BOLD Contrast Response in Human Brain during Simple Breathhold Measured at 1.5 Tesla. Proceedings of the ISMRM 6th Annual Meeting.

[304] Li T.-Q., Kastrup A., Takakashi A.M., Moseley M.E. (1999). Functional MRI of human brain during breath holding by BOLD and FAIR techniques. Neuroimage.

[305] Tillakaratne N.J., Medina-Kaaawe L., Gibson K.M. (1995). Gamma-aminobutyric acid (GABA) metabolism in mammalian neural and non-neural tissues. Comp. Biochem. Physiol. A Physiol..

[306] Lennox W.G. (1931). Certain chemical and physiological conditions which may influence seizures. Assoc. Res. Nerv. Ment. Dis. Proc..

[307] Lennox W.G., Behnke A.R. (1936). Effect of increased oxygen pressure on the seizures of epilepsy. Arch. Neurol. Psychiatry.

[308] Toman J.E.P., Davis J.P. (1949). The effects of drugs upon the electrical activity of the brain. Pharm. Rev..

[309] Swanson A.G., Stavney L.S., Stavney F. (1958). Effects of blood pH and carbon dioxide on cerebral electrical activity. Neurology.

[310] Withrow C.D., Purpara D.P., Penry J.K., Woodburg D.M., Tower D.B., Water R.D. (1972). Systemic Carbon Dioxide Derangements. Experimental Models of Epilepsy.

[311] Kennealy J.A., Penovich P.E., Moore-Nease S.E. (1986). EEG and spectral analysis in acute hyperventilation. Electroencephalogr. Clin. Neurophysiol..

[312] Gibbs F.A., Davis H., Lennox W.G. (1935). The electroencephalogram in epilepsy and in condition of impaired consciousness. Arch. Neurol. Psychiatry.

[313] Meyer J.S., Waltz A.G. (1960). Arterial oxygen saturation and alveolar carbon dioxide during electroencephalography. I. Comparison of hyperventilation and induced hypoxia in subjects without brain disease. Arch. Neurol..

[314] Meyer J.S., Waltz A.G. (1960). Arterial oxygen saturation and alveolar carbon dioxide during electroencephalography. II. Comparison of hyperventilation and induced hypoxia in subjects with epilepsy. Arch. Neurol..

[315] Sokoloff L. (1960). The effects of carbon dioxide on the cerebral circulation. Anesthesiology.

[316] Siesjo B.K., Messeter K., Siesjo B.K., Sprensen S.C. (1971). Factors Determining Intracellular pH. Ion Homeostasis of the Brain.

[317] Bode H., Puglia E. (1992). Intra-individual variability of cerebral blood flow velocities in children while awake and asleep. Ultraschall Med..

[318] VonBulow I., Ebert T., Rockstroh B., Lutzenberger W., Canavan A., Birnbaumer N. (1989). Effects of hyperventilation on EEG frequency and slow wave potentials in relation to an anticonvulsant and epilepsy. J. Neuropsychophysiol..

[319] Rockstroh B. (1990). Hyperventilation-induced EEG changes in humans and their modulation by anticonvulsant drug. Epilepsy Res..

[320] Plum F., Pasner J.B., Smith W.W. (1968). Effect of hyperbaric-hyperoxic hyperventilation on blood, brain, and CSF lactate. Am. J. Physiol..

[321] Stehling M.K., Schmitt F., Ladebeck R. (1993). Echo-planar MR imaging of human brain oxygenation changes. J. Magn. Reson. Imaging.

[322] Prisman E., Slessarev M., Han J., Poublanc J., Mardimae A., Crawley A., Fisher J., Mikulis D. (2008). Comparison of the effects of independently-controlled end-tidal PCO_2_ and PO_2_ on blood oxygen level-dependent (BOLD) MRI. J. Magn. Reson. Imaging..

[323] Weckesser M., Posse S., Olthoff U., Kemna L., Drager S., Muller-Gartner H.W. (1999). Functional imaging of the visual cortex with bold-contrast MRI: Hyperventilation decreases signal response. Magn. Res. Med..

[324] Riley T.I. (1982). Epilepsy-or merely hyperventilation?. Emerg. Med..

[325] Chapman A.G., Pedley T.A., Meldrum B.S. (1995). Excitatory Neurotransmission and Antiepileptic Drug Development in Status Report. Recent Advances Epilepsy.

[326] Chanutin A., Curnish R.R. (1967). Effect of organic and inorganic phosphates on the oxygen equilibrium of human erythrocytes. Arch. Biochem. Biophys..

[327] Brewer G.J., Eaton J.W. (1971). Erythrocyte metabolism: Interaction with oxygen transport. Science.

[328] Woodson R.D. (1979). Physiological significance of oxygen dissociation curve shift. Crit. Care Med..

[329] Fried R., Rubin S.R., Carlton R.M., Fox M.C. (1984). Behavioral control of intractable idiopathic seizures: 1. Self-regulation of end-tidal carbon dioxide. Psychosom. Med..

[330] Pincus J.H., Tucker G.J. (1985). Behavioral Neurology.

[331] Klein D.F., Klein D.F., Rakin J.G. (1981). Anxiety Reconceptualized. Anxiety: New Research and Changing Concepts.

[332] Gorman J.M., Laebowitz M.R., Fryer A.J., Stein J.A. (1989). A neuroanatomical hypothesis for panic disorder. Am. J. Psychiatry.

[333] Engel G.L., Ferriss E.B., Logan M. (1947). Hyperventilation: Analysis of clinical symptomatology. Ann. Int. Med..

[334] Hagberg B., Aicardi J., Dias K., Ramos O. (1983). A progressive syndrome of autism, dementia, ataxia and loss of purposeful hand use in girls: Rett’s syndrome, a report of 35 cases. Am. Neurol..

[335] Bruno-Golden P., Holmes G.L. (1993). Hyperventilation-induced seizures in mentally impaired children. Seizure.

[336] Ogunyemi A.O., Gomez M.R., Klass D.W. (1988). Seizures induced by exercise. Neurology.

[337] Boyle R. (1660). New Experiments Physico-Mechanical, Touching the Spring of Air and Its Effects.

[338] Poussouant P., Cadilhoc J., Pternitis C., Baldy-Moulinier M. (1967). Épilepsie-temporale et decharges ammoniques provoqueés per l’anoxie exprive. Rev. Neurol..

[339] Poussouant P., Cadilhoc J., Pternitis C., Baldy-Moulinier M. (1967). Temporal epilepsy and hippocampal discharges induced by oxyprivic anoxia. Electroencephalogr. Clin. Neurophysiol..

[340] Nelson S.R., Mantz M.L. (1971). Brain energy reserve levels at the onset of convulsions in hypoxic mice. Life Sci. I.

[341] Tower D.B., Roberts E. (1960). The Administration of Gamma-Aminobutyric Acid to Man: Systemic Effects and Anticonvulsant Actions. Inhibition in the Nervous System and Gamma-Aminobutyric Acid.

[342] Gellhorn E., Heymans C. (1948). Differential action of anoxia, asphyxia and carbon dioxide on normal and convulsive potentials. J. Neurophysiol..

[343] Wood J.D., Purpura D.P., Penry J.K., Woodbury D.M., Tower D.B., Walter R.D. (1972). Systemic Oxygen Derangements. Experimental Models of Epilepsy.

[344] Lovell R.A., Elliott S.J., Elliott K.A. (1963). The gamma-aminobutyric acid and Factor I content of brain. J. Neurochem..

[345] Wood J.D., Watson W.J., Ducker A.J. (1963). Gamma-aminobutyric acid and oxygen poisoning. J. Neurochem..

[346] Bert P., Hitchcock M.A., Hitchcock F.A. (1943). La Pression Barométrique.

[347] Stadie W.C., Riggs B.C., Haugaard N. (1944). Oxygen poisoning. Am. J. Med. Sci..

[348] Bean J.W. (1945). Effects of oxygen at increased pressure. Physiol. Rev..

[349] Lambertson C.J., Fenn W.O., Rahn H. (1965). Effects of Oxygen at High Partial Pressure. Handbook of Physiology.

[350] Lambertson C.J., Ewing J.H., Kough R.H., Gould R., Stroud M.W. (1955–1956). Oxygen toxicity. J. Appl. Physiol..

[351] Wood J.D. (1966). Development of a strain of rats with greater than normal susceptibility to oxygen poisoning. Can. J. Physiol. Pharmacol..

[352] Novotny E.J., Hyder F., Shevell M., Rothman D.L. (1999). GABA changes with vigabatrin in the developing human brain. Epilepsia.

[353] Petroff O.A., Rothman D.L., Behar K.L., Collins T.L., Mattson R.H. (1996). Human brain GABA levels rise rapidly after initiation of vigabatrin therapy. Neurology.

[354] Webb W.B., Wauquier A., Dugovic C., Radulovacki M. (1989). Slow Wave Sleep and Prior Wakefulness, Sleep Time, and Stability across Time. Slow Wave Sleep: Physiological, Pathophysiological and Functional Aspects.

[355] Hobson J.A. (1989). Sleep: Behavior and Cellular Mechanism. Encyclopedia of Neuroscience.

[356] Datta S., Calvo J., Quatrochi J., Hobson J.A. (1991). Long-term enhancement of REM sleep following cholinergic stimulation. Neuroreport.

[357] Steriade M., Contreras D., Amzica F. (1994). Synchronized sleep oscillations and their paroxysmal developments. Trends Neurosci..

[358] Steriade M., Nunez A., Amzica F. (1993). Intracellular analysis of relations between the slow (<1 Hz) neocortical oscillation and other sleep rhythms of the electroencephalogram. J. Neurosci..

[359] Montplaiser J., Laverdière M., Saint-Hilaire J.M., Rouleau I. (1987). Noctural sleep recording in partial epilepsy: A study with depth electrodes. J. Clin. Neurophys..

[360] Chokroverty S., Chokroverty S. (1994). Sleep and Epilepsy. Sleep Disorders Medicine.

[361] Juhasz G., Emri Z., Kekesi K., Pungor K. (1989). Local perfusion of the thalamus with GABA increases sleep and induces long-lasting inhibition of somatosensory event-related potentials in cats. Neurosci. Lett..

[362] Feinberg I., Wauquier A., Dugovic C., Radulovacki M. (1989). Effects of Maturation and Aging on Slow-Wave Sleep in Man: Implications for Neurobiology. Slow Wave Sleep: Physiological, Pathophysiological and Functional Aspects.

[363] Reddy D.S. (2000). Enhanced anticonvulsant activity of ganoxolone after neurosteroid withdrawal in a rat model of catamenial epilepsy. J. Pharmacol. Exp. Ther..

[364] Reddy D.S., Rogawski M.A. (2001). Enhanced anticonvulsant activity of neuroactive steroids in a rat model of catamenial epilepsy. Epilepsia.

[365] Sim J.A., Skynner M.J., Herbison A.E. (2001). Direct regulation of postnatal GnRH neurons by the progesterone derivative allopregnanolone in the mouse. Endocrinology.

[366] Mennerick S., Zeng C.M., Benz A., Shen W., Izumi Y., Evers A.S., Covey D.F., Zorumski C.F. (2001). Effects on gamma aminobutyric acid (GABA) (A) receptors of a neuroactive steroid that negatively modulates glutamate neurotransmission and augments GABA neurotransmission. Mol. Pharmacol..

[367] Puia G., Belelli D. (2001). Neurosteroids on our minds. Trends Pharmacol. Sci..

[368] Kawata M., Yuri K., Morimoto M. (1994). Steroid hormone effects on gene expression, neuronal structure and differentiation. Horm. Behav..

[369] Sar M., Stumpf W.E., Tappaz M.L. (1983). Localization of ^3^H-estradiol in preoptic GABAergic neurons. Fed. Proc..

[370] Wallis C.J., Luttge W.G. (1980). Influence of estrogen and progesterone on glutamic acid decarboxylase activity in discrete regions of rat Brain. J. Neurochem..

[371] Mansky T., Mestres-Ventura P., Wuttke W. (1982). Involvement of GABA in the feedback action of estradiol on gonadotropin and prolactin release: Hypothalamic GABA and catecholamine turnover rates. Brain Res..

[372] Ramirez V.D., Feder H.H., Sawyer C.H., Martini L., Ganong W.F. (1984). The Role of Brain Catecholamines in the Regulation of LH Secretion: A Critical Inquiry. Frontiers in Neuroendocrinology.

[373] Majewska M.D., Mienville J.M., Vicini S. (1988). Neurosteroid pregnenolone sulfate antagonizes electrophysiological responses to GABA in neurons. Neurosci. Lett..

[374] Mienville J.M., Vicini S. (1989). Pregnenolone sulfate antagonizes GABA_A_ receptor-mediated currents via a reduction of channel opening frequency. Brain Res..

[375] Kis Z., Budai D., Imre G., Farkas T., Horvath S., Toldi J. (2001). The modulatory effect of estrogen on the neuronal activity in the barrel cortex of the rat. An electrophysiological study. Neuroreport.

[376] Saleh T.M., Saleh M.C. (2001). Inhibitory effect of 17 beta-estradiol in the parabrachial nucleus is mediated by GABA. Brain Res..

[377] Mitsushima D., Hei D.L., Terasawa E. (1994). gamma-aminobutyric acid is an inhibitory neurotransmitter restricting the release of luteinizing hormone-releasing hormone before the onset of puberty. Proc. Natl. Acad. Sci. USA.

[378] Adler B.A., Crowley W.R. (1986). Evidence for gamma-aminobutyric acid modulation of ovarian hormone effects on luteinizing hormone secretion and hypothalamic catecholamine activity in the female rat. J. Endocrinol..

[379] Celotti F., Apud J.A., Melcangi R.C., Masotto C., Tappaz M., Racagni G. (1986). Endocrine modulation of gamma-aminobutyric acidergic innervation in the rat fallopian tube. Endocrinology.

[380] Terasawa E., Plant T.M., Lee P.A. (1995). Mechanisms Controlling the Onset of Puberty in Primates: The Role of GABAergic Neurons. The Neurobiology of Puberty.

[381] Mayo W., Dellu F., Robel P., Cherkaoui J., le Moal M., Baulieu E.E., Simon H. (1993). Infusion of neurosteroids into the nucleus basalis magnocellularis affects cognitive processes in the rat. Brain Res..

[382] Garcia-Segura L.M., Chowen J.A., Dueñas M., Torres-Aleman I., Naftolin F. (1994). Gonadal steroids as promoters of neuro-glial plasticity. Psychoneuroendocrinology.

[383] Matsumoto A. (2000). Sexual Differentiation of the Brain.

[384] Gould E., Woolley C.S., Frankfurt M., McEwen B.S. (1990). Gonadal steroids regulate dendritic spine density in hippocampal pyramidal cells in adulthood. J. Neurosci..

[385] Frankfurt M., McEwen B.S. (1991). Estrogen increases axodendritic synapses in the VMN of rats after ovariectomy. Neuroreport.

[386] Gur R.C., Gur R.E., Obrist W.D., Skolnick B.E., Reivich M. (1987). Age and regional cerebral blood flow at rest and during cognitive activity. Arch. Gen. Psychiatry.

[387] Thomas D.J., Marshall J., Ross Russell R.W., Wetherly-Mein G., Du Boulay G.H., Pearson T.C., Syman L., Zilkha E. (1977). Effects of haematocrit on cerebral blood-flow in man. Lancet.

[388] Vriens E.M., Kraaier V., Musbach M., Wiencke G.H., van Huffelen A.C. (1989). Transcranial pulse Doppler measurements of blood velocity in the middle cerebral artery: Reference values at rest and during hyperventilation in healthy volunteers in relation to age and sex. Ultrasound Med. Biol..

[389] Kastrup A., Thomas C., Hartmann C., Schabet M. (1997). Sex dependency of cerebrovascular CO_2_ reactivity in normal subjects. Stroke.

[390] Kastrup A., Dichgans J., Niemeier M., Schabet M. (1998). Changes of cerebrovascular CO_2_ reactivity during normal aging. Stroke.

[391] Frankfurt M., Fuchs E., Wuttke W. (1984). Sex differences in gamma-aminobutyric acid and glutamate concentrations in discrete rat brain nuclei. Neurosci. Lett..

[392] Grattan D.R., Selmanoff M. (1997). Sex differences in the activity of gamma-aminobutyric acidergic neurons in the rat hypothalamus. Brain Res..

[393] Herbison A.E., Robinson J.E., Skinner D.C. (1993). Distribution of estrogen receptor- immunoreactive cells in the preoptic area of the ewe: Co-localization with glutamic acid decarboxylase but not luteinizing hormone-releasing hormone. Neuroendocrinology.

[394] Vijayan E., McCann S.M. (1978). The effects of intraventricular injection of gamma-aminobutyric acid (GABA) on prolactin and gonadotropin release in conscious female rats. Brain Res..

[395] Manev H., Pericić D. (1987). Sex difference in the turnover of GABA in the rat substantia nigra. J. Neural. Transm..

[396] McEwen B.S. (1991). Non-genomic and genomic effects of steroids on neural activity. Trends Pharmacol. Sci..

[397] Sagrillo C.A., Selmanoff M. (1994). Castration decreased mRNA levels encoding glutamic acid decarboxylase within that region of the medial preoptic area which coincides with the sexually dimorphic nucleus (SDN-mPOA). Soc. Neurosci. Abst..

[398] Woolley D., Timiras P.S. (1962). The gonad-brain relationship: Effects of female sex hormones on electroshock convulsions in the rat. Endocrinology.

[399] Bäckström T. (1976). Epileptic seizures in women related to plasma estrogen and progesterone during the menstrual cycle. Acta Neurol. Scand..

[400] Logothetis J., Harner R., Morrell F., Torres F. (1959). The role of estrogens in catamenial exacerbation of epilepsy. Neurology.

[401] Wray S., Fueshko S.M., Kusano K., Gainer H. (1996). GABAergic neurons in the embryonic olfactory pit/vomeronasal organ: Maintenance of functional GABAergic synapses in olfactory explants. Dev. Biol..

[402] Tobet S.A., Chickering T.W., King J.C., Stopa E.G., Kim K., Kuo-Leblank V., Schwarting G.A. (1996). Expression of gamma-aminobutyric acid and gonadotropin-releasing hormone during neuronal migration through the olfactory system. Endocrinology.

[403] Fueshko S.M., Key S., Wray S. (1998). GABA inhibits migration of luteinizing hormone-releasing hormone neurons in embryonic olfactory explants. J. Neurosci..

[404] Shepherd G., Greer C., Shepherd G. (1990). Olfactory Bulb. The Synaptic Organization of the Brain.

[405] Trombley P.Q., Shepherd G.M. (1993). Synaptic transmission and modulation in the olfactory bulb. Curr. Opin. Neurobiol..

[406] Nakanishi S. (1994). Metabotropic glutamate receptors: Synaptic transmission, modulation and plasticity. Neuron.

[407] Esfandyari T., Camilleri M., Busciglio I., Burton D., Baxter K., Zinsmeister A.R. (2007). Effects of a cannabinoid receptor agonist on colonic motor and sensory functions in humans: A randomized, placebo-controlled study. Am. J. Physiol. Gastrointest. Liver Physiol..

[408] Ralevic V., Kendall D.A. (2009). Cannabinoid modulation of perivascular sympathetic and sensory neurotransmission. Curr. Vasc. Pharmacol..

[409] Hoffman A.F., Lupica C.R. (2000). Mechanisms of cannabinoid inhibition of GABA_A_ synaptic transmission in the hippocampus. J. Neurosci..

[410] Sullivan J.M. (2000). Cellular and molecular mechanisms underlying learning and memory impairments produced by cannabinoids. Learn. Mem..

[411] Egerton A., Allison C., Brett R.R., Pratt J.A. (2006). Cannabinoids and prefrontal cortical function: Insights from preclinical studies. Neurosci. Biobehav. Rev..

[412] Matsuda L.A., Lolait S.J., Brownstein M.J., Young A.C., Bonner T.I. (1990). Structure of a cannabinoid receptor and functional expression of the cloned cDNA. Nature.

[413] Martin B.R., Welch S.P., Abood M.E. (1994). Progress toward understanding the cannabinoid receptor and its second messenger systems. Adv. Pharmacol..

[414] Martin B.R., Adelman G., Smith B.H. (1999). Marijuana. Encyclopedia of Neuroscience.

[415] Löscher W. (1979). 3-Mercaptopropionic acid: Convulsant properties, effects on enzymes of the gamma-aminobutyrate system in mouse brain and antagonism by certain anticonvulsant drugs, aminooxyacetic acid and gabaculine. Biochem. Pharmacol..

[416] Revuelta A.V., Cheney D.L., Wood P.L., Costa E. (1979). GABAergic mediation in the inhibition of hippocampal acetylcholine turnover rate elicited by delta 9-tetrahydrocannabinol. Neuropharmacology.

[417] Mechoulam R. (1986). Cannabinoids as Therapeutic Agents.

[418] Leader J.P., Koe B.K., Weissman A. (1981). GABA-like actions of levonantradol. J. Clin. Pharmacol..

[419] Costa E., Cheney D.L., Murray T.F. (1981). Levonantradol-induced inhibition of acetylcholine turnover in rat hippocampus and striatum. J. Clin. Pharmacol..

[420] Edery H., Gottesfeld Z. (1975). The gamma-aminobutyric acid system in rat cerebellum during cannabinoid-induced cataleptoid state. Br. J. Pharmacol..

[421] Feeney D.M. (1978). Marihuana and epilepsy: Paradoxical anticonvulsant and convulsant effects. Adv. Biosci..

[422] Pacheco M.A., Ward S.J., Childers S.R. (1993). Identification of cannabinoid receptors in cultures of rat cerebellar granule cells. Brain Res..

[423] Pertwee R.G., Balfour D.J.K. (1990). The Central Neuropharmocology of Psychotropic Cannabinoids. Psychotropic Drugs of Abuse.

[424] Wickens A.P., Pertree R.G. (1995). Effect of delta-9-tetrahydrocannabinol on circling in rats induced by intranigral muscimol administration. Eur. J. Pharmocol..

[425] Pertwee R.G., Greentree S.G., Swift P.A. (1988). Drugs which stimulate or facilitate central GABAergic transmission interact synergistically with delta-9-tetrahydrocannabinol to produce marked catalepsy in mice. Neuropharmacology.

[426] Shen M., Piser T.M., Seybold V.S., Thayer S.A. (1996). Cannabinoid receptor agonists inhibit glutamatergic synaptic transmission in rat hippocampal cultures. J. Neurosci..

[427] Joy J.E., Watson S.J., Benson J.A. (2000). Marijuana and Medicine.

[428] Agurell S., Dewey W.L., Willtette R.D. (1984). The Cannabinoids: Chemical, Pharmacologic, and Therapeutic Aspects.

[429] Pertwee R.G., Browne S.E., Ross T.M., Stretton C.D. (1991). An investigation of the involvement of GABA in certain pharmacological effects of delta-9-tetrahydrocannabinol. Pharmacol. Biochem. Behav..

[430] Martin B.R. (1986). Cellular effects of cannabinoids. Pharmacol. Rev..

[431] Consroe P., Mechoulam R. (1987). Anticonvulsant and neurotoxic effects of tetrahydrocannabinol stereoisomers. NIDA Res. Monogr..

[432] Zygmunt P.M., Andersson D.A., Hogestatt E.D. (2002). Delta 9-tetrahydrocannabinol and cannabinol activate capsaicin-sensitive sensory nerves via a CB_1_ and CB_2_ cannabinoid receptor-independent mechanism. J. Neurosci..

[433] Cronholm B. (1951). Phantom limb in amputees; a study of changes in the integration of centripetal impulses with special reference to referred sensations. Acta Psychiatr. Neurol. Scand. Suppl..

[434] Kew J.J., Halligan P.W., Marshall J.C., Passingham R.E., Rothwell J.C., Ridding M.C., Marsden C.D., Brooks D.J. (1997). Abnormal access of axial vibrotactile input to deafferented somatosensory cortex in human upper limb amputees. J. Neurophysiol..

[435] Knecht S., Henningsen H., Höhling C., Elbert T., Flor H., Pantev C., Taub E. (1998). Plasticity of plasticity? Changes in the pattern of perceptual correlates of reorganization after amputation. Brain.

[436] Spitzer M., Böhler P., Weisbrod M., Kischka U. (1995). A neural network model of phantom limbs. Biol. Cybern..

[437] Ojemann J.G., Silbergeld D.L. (1995). Cortical stimulation mapping of phantom limb rolandic cortex. Case Report. J. Neurosurg..

[438] Jones E.G., Pons T.P. (1998). Thalamic and brain stem contributions to large-scale plasticity of primate somatosensory cortex. Science.

[439] Craig A.D., Reiman E.M., Evans A., Bushnell M.C. (1996). Functional imaging of an illusion of pain. Nature.

[440] Borsook D., Becerra L., Fishman S., Edwards A., Jennings C.L., Stojanovic M., Papinicolas L., Ramachandran V.S., Gonzales R.G., Breiter H. (1998). Acute plasticity in the human somatosensory cortex following amputation. Neuroreport.

[441] Flor H., Elbert T., Knecht S., Wienbruch C., Pantev C., Birbaumers N., Larbig W., Taub E. (1995). Phantom-limb pain as a perceptual correlate of cortical reorganization following arm amputation. Nature.

[442] Zilstorff K. (1966). Parosmia. J. Laryngol. Otol..

[443] Zilstorff K., Herbild O. (1979). Parosmia. Acta Otolaryngol. Suppl..

[444] Korczyn A.D. (2001). Hallucination in Parkinson’s disease. Lancet.

[445] Gsell W., Strein I., Krause U., Riederer P. (1997). Neurochemical abnormalities in Alzheimer’s disease and Parkinson’s disease: A comparative review. J. Neural. Transm..

[446] Cummings J.L. (2000). Cholinesterase inhibitors: Expanding applications. Lancet.

[447] Haussermann P., Ceballos-Baumann A., Reinbold H., Goecker P., Schröder S. (2001). Applications of cholinesterase inhibitors. Lancet.

[448] Creese I., Burt D., Snyder S.H., Iverson L.L., Iverson S.D., Snyder S.H. (1978). Biochemical Actions of Neuroleptic Drugs: Focus on Dopamine Receptor. Handbook of Psycho-Pharmacology.

[449] Goetz C.G., Vogel C., Tanner C.M., Stebbins G.T. (1998). Early dopaminergic drug-induced hallucinations in parkinsonian patients. Neurology.

[450] Feinberg A.P., Snyder S.H. (1975). Phenothiazine drugs: Structure-activity relationships explained by a conformation that mimics dopamine. Proc. Natl. Acad. Sci. USA.

[451] Kamiya Y., Andoh T., Furuya R., Hattori S., Watanabe I., Sasaki T., Ito H., Okumura F. (1999). Comparison of the effects of convulsant and depressant barbiturate stereoisomers on AMPA-type glutamate receptors. Anesthesiology.

[452] Yokoro C.M., Pesquero S.M., Turchetti-Maia R.M., Francischi J.N., Tatsuo M.A. (2001). Acute phenobarbital administration induces hyperalgesia: Pharmacological evidence for the involvement of supraspinal GABA-A receptors. Braz. J. Med. Biol. Res..

[453] Akk G., Steinbach J.H. (2000). Activation and block of recombinant GABA(A) receptors by pentobarbitone: A single channel study. Br. J. Pharmacol..

[454] Ortho-McNeil H. (2002). Thioridazine. Physicians Desk Reference.

[455] Mylan T. (2002). Haloperidol. Physicians Desk Reference.

[456] Fenwick P. (1994). The behavioral treatment of epilepsy generation and inhibition of seizures. Neurol. Clin..

[457] Pritchard P., Holmstrom V., Giacinto J. (1985). Self-abatement of complex partial seizures. Ann. Neurol..

[458] Efron R. (1956). The effect of olfactory stimuli in arresting uncinate fits. Brain.

[459] Iwata B.A., Lorentzson A.M. (1976). Operant control of seizure-like behavior in an institutionalized retarded adult. Behav. Ther..

[460] Gardner J.E. (1967). Behavior therapy treatment approach to a psychogenic seizure case. J. Consult. Psychol..

[461] Sterman M.B., Birk L. (1973). Neurophysiological and Clinical Studies of Sensori-Motor EEG Biofeedback Training: Some Effects of Epilepsy. Biofeedback: Behavior Medicine.

[462] Dahl J., Melin L., Brorson L.O., Schollin J. (1985). Effects of a broad-spectrum behavior modification treatment program on children with refractory epileptic seizures. Epilepsia.

[463] Dahl J., Melin L., Lund L. (1987). Effects of a contingent relaxation treatment program on adults with refractory epileptic seizures. Epilepsia.

[464] Tassinari C.A. (1968). Suppression of focal spikes by somatosensory stimuli. Electroencephalogr. Clin. Neurophysiol..

[465] Gellhorn E. (1947). Effect of afferent impulses on cortical suppressor areas. J. Neurophysiol..

[466] Barker S.H., Gellhorn E. (1947). Influence of suppressor areas on afferent impulses. J. Neurophysiol..

[467] Mostofsky D.I., Balaschak B.A. (1977). Psychobiological control of seizures. Psychol. Bull..

[468] Morrell M.J. (1993). Differential diagnosis of seizures. Neurol. Clin..

[469] Alper K. (1994). Nonepileptic seizures. Neurol. Clin..

[470] Alper K.R., Devinsky O., Perrine K., Vasquez B., Luciano D. (1995). Psychiatric classification of nonconversion nonepileptic seizures. Arch. Neurol..

[471] Altman H., Collins M., Mundy P. (1997). Subclinical hallucinations and delusions in nonpsychotic adolescents. J. Child Psychol. Psychiatry.

[472] Guberman A. (1982). Psychogenic pseudoseizures in non-epileptic patients. Can. J. Psychiatry.

[473] Pakalnis A., Drake M.E., Phillips B. (1991). Neuropsychiatric aspects of psychogenic status epilepticus. Neurology.

[474] Merlis J.K., Vinken J.P., Bruyn G.W. (1974). Reflex Epilepsy. Handbook of Clinical Neurology: The Epilepsies.

[475] Forster F.M., Forster F.M. (1977). Behavioral Therapy of Reflex Epilepsy: Maintenance or Reinforcement of Therapy. Reflex Epilepsy, Behavior Therapy and Conditional Reflexes.

[476] Rotaccio A.L. (1994). Reflex seizures. Neurol. Clin..

[477] Jeavons P.M., Harding G.F. (1975). Photosensitive Epilepsy: A Review of the Literature and a Study of 460 Patients.

[478] Jeavons P.M., Laidlaw J., Richens A. (1982). Photosensitive Epilepsy. A Textbook of Epilepsy.

[479] Behrman S., Wyke B.D. (1958). Vestibulogenic seizures. Brain.

[480] Cantor F.K. (1971). Vestibular-temporal lobe connections demonstrated by induced seizures. Neurology.

[481] Critchley M. (1937). Musicogenic epilepsy. Brain.

[482] Abenson M.H. (1969). Epileptic fits provoked by taste. Br. J. Psychiatry.

[483] Tedrus G.M., Albertin M.C., Odashima N.S., Fonseca C. (1991). Partial motor seizures induced by movement in diabetic patients. Arq. Neuropsiquiatr..

[484] Penfield W., Erickson T.C. (1941). Epilepsy and Cerebral Localization: A Study of the Mechanism, Treatment and Prevention of Epileptic Seizures.

[485] Cirignotta F., Marcacci G., Lugaresi E. (1977). Epileptic seizures precipitated by eating. Epilepsia.

[486] Fiol M.E., Leppik I.E., Pretzel K. (1986). Eating epilepsy. EEG and clinical study. Epilepsia.

[487] Alajouahine T., Gastaut H. (1995). Synkinesis-startle and epilepsy startle triggered by unexpected sensory and sensitive factors. I. Anatomical and clinical data on 15 cases. Rev. Neurol. (Paris).

[488] Bickford R.G., Whelan J.L., Klass D.W., Corbin K.B. (1956). Reading epilepsy: Clinical and electroencephalographic studies of a new syndrome. Trans. Am. Neurol. Assoc..

[489] Ingvar D.H., Nyman G.E. (1962). Epilepsia arithmetices. A new psychological trigger mechanism in a case of epilepsy. Neurology.

[490] Clementi A. (1931). Storicizzazione circoscritta del lobo piriforme del cervello ed epilessia sperimentale da stimoli odoriferi. Arch. Fisiol..

[491] Kasteleijn-Nolst Trenité D.G. (1989). Photosensitivity in epilepsy. Electrophysiological and clinical correlates. Acta Neurol. Scand. Suppl..

[492] Doose H., Gerken H., Hien-Völpel K.F. (1969). Genetics of photosensitive epilepsy. Neuropediatrie.

[493] Lesser R.P., Pedley T.A., Meldrum B.J. (1985). Psychogenic Seizures. Recent Advances in Epilepsy 2.

[494] Trimble M. (1983). Pseudo problems pseudoseizures. Br. J. Hosp. Med..

[495] Ramani V., Gumnit R. (1986). Intensive Monitoring of Psychogenic Seizures, Aggression and Dyscontrol Syndromes. Advances in Neurology.

[496] Betts T. (1990). Pseudoseizures: Seizures that are not epilepsy. Lancet.

[497] Gates J.R., Luciano D., Devinsky O., Devinsky O., Theodore W.H. (1991). The Classification and Treatment of Nonepileptic Events. Epilepsy and Behavior.

[498] Lesser R.P., Lueders H., Dinner D.S. (1983). Evidence for epilepsy is rare in patients with psychogenic seizures. Neurology.

[499] Lelliott P.T., Fenwick P. (1991). Cerebral pathology in pseudoseizures. Acta Neurol. Scand..

[500] Pizzo P.A. (2013). Lessons in pain relief – a personal postgraduate experience. N. Engl. J. Med..

[501] Jaspers K. (1963). General Psychopathology.

[502] Greenberg M.S., Serby M.J., Chobor K.L. (1992). Olfactory Hallucinations. Science of Olfaction.

[503] Tilley H. (1895). hree cases of parosmia; causes, treatment, *etc.*. Lancet.

[504] Allaez J., Dongiers S. (1955). EEG correlations of olfactory hallucinations. Am. Med. Psychol..

[505] Fahn S., Bressman S.B., Marsden C.D. (1998). Classification of dystonia. Adv. Neurol..

[506] Fahn S., Findley L.J., Capildeo R. (1984). Atypical Tremors, Rare Tremors and Unclassified Tremors. Movement Disorders: Tremor.

[507] Jackson J.H. (1881). Epileptiform convulsions from cerebral disease. Trans. Int. Med. Congr..

[508] Florence S.L., Taub H.B., Kaas J.H. (1998). Large-scale sprouting of cortical connections after peripheral injury in adult macaque monkeys. Science.

